# Optimising the Diagnosis of Prostate Cancer in the Era of Multiparametric Magnetic Resonance Imaging: A Cost-effectiveness Analysis Based on the Prostate MR Imaging Study (PROMIS)

**DOI:** 10.1016/j.eururo.2017.08.018

**Published:** 2018-01

**Authors:** Rita Faria, Marta O. Soares, Eldon Spackman, Hashim U. Ahmed, Louise C. Brown, Richard Kaplan, Mark Emberton, Mark J. Sculpher

**Affiliations:** a Centre for Health Economics, University of York, York, UK; b University of Calgary, Alberta, Canada; c Imperial Urology—Division of Surgery, Imperial College London, London, UK; d Medical Research Council Clinical Trials Unit at University College London, London, UK; e Division of Surgery and Interventional Science, Faculty of Medical Sciences, University College London, London, UK; f Department of Urology, University College London Hospitals NHS Foundation Trust, London, UK; g Imperial Urology, Imperial College Healthcare NHS Trust

**Keywords:** Prostate cancer, Cost-effectiveness analysis, Magnetic resonance imaging, Prostate biopsy, Model-based analysis

## Abstract

**Background:**

The current recommendation of using transrectal ultrasound-guided biopsy (TRUSB) to diagnose prostate cancer misses clinically significant (CS) cancers. More sensitive biopsies (eg, template prostate mapping biopsy [TPMB]) are too resource intensive for routine use, and there is little evidence on multiparametric magnetic resonance imaging (MPMRI).

**Objective:**

To identify the most effective and cost-effective way of using these tests to detect CS prostate cancer.

**Design, setting, and participants:**

Cost-effectiveness modelling of health outcomes and costs of men referred to secondary care with a suspicion of prostate cancer prior to any biopsy in the UK National Health Service using information from the diagnostic Prostate MR Imaging Study (PROMIS).

**Intervention:**

Combinations of MPMRI, TRUSB, and TPMB, using different definitions and diagnostic cut-offs for CS cancer.

**Outcome measurements and statistical analysis:**

Strategies that detect the most CS cancers given testing costs, and incremental cost-effectiveness ratios (ICERs) in quality-adjusted life years (QALYs) given long-term costs.

**Results and limitations:**

The use of MPMRI first and then up to two MRI-targeted TRUSBs detects more CS cancers per pound spent than a strategy using TRUSB first (sensitivity = 0.95 [95% confidence interval {CI} 0.92–0.98] vs 0.91 [95% CI 0.86–0.94]) and is cost effective (ICER = £7,076 [€8350/QALY gained]). The limitations stem from the evidence base in the accuracy of MRI-targeted biopsy and the long-term outcomes of men with CS prostate cancer.

**Conclusions:**

An MPMRI-first strategy is effective and cost effective for the diagnosis of CS prostate cancer. These findings are sensitive to the test costs, sensitivity of MRI-targeted TRUSB, and long-term outcomes of men with cancer, which warrant more empirical research. This analysis can inform the development of clinical guidelines.

**Patient summary:**

We found that, under certain assumptions, the use of multiparametric magnetic resonance imaging first and then up to two transrectal ultrasound-guided biopsy is better than the current clinical standard and is good value for money.

## Take Home Message

The use of multiparametric magnetic resonance imaging before transrectal ultrasound-guided biopsy can detect more clinically significant prostate cancer and be cost effective compared with the use of imaging post-biopsy.

## 1 Introduction

Multiparametric magnetic resonance imaging (MPMRI) is increasingly being recommended for the diagnosis of clinically significant (CS) prostate cancer, if the initial biopsy proves negative [1], [2]. An alternative approach is to begin with MPMRI imaging to inform who needs a biopsy and, in those who need it, how it might be best conducted [3]. Recent studies have reported encouraging results on the performance of MPMRI in detecting CS prostate cancer [3], [4], [5]. The Prostate MR Imaging Study (PROMIS) was the largest accuracy study on the use of MPMRI and transrectal ultrasound-guided biopsy (TRUSB) in the diagnosis of prostate cancer [4]. Using template mapping biopsy (TPMB) as the reference standard, it was found that MPMRI had better sensitivity for CS prostate cancer compared with TRUSB but worse specificity [4]. It is therefore necessary to explore how best to combine these tests and the consequences of incorrect diagnosis on health outcomes. This study aims to identify the combinations of tests—diagnostic strategies—that detect the most CS cancers per pound spent in testing and achieve the maximum health given their cost to the healthcare service.

## 2 Patients and methods

The target population was men at risk of prostate cancer referred to secondary care for further investigation [4], [6]. The perspective was the UK National Health Service (NHS). Costs were expressed in pound sterling from a 2015 price base. The time horizon is the population's predicted lifetime. Costs incurred and health outcomes attained in the future were discounted to present values at 3.5% per annum [7].

### 2.1 Diagnostic strategies

The diagnostic strategies consisted of clinically feasible combinations of MPMRI, TRUSB, and TPMB, in addition to the use of TRUSB and TPMB in isolation (Table 1; details in the Supplementary material, section 1.1). These included strategies using MPMRI to decide whether a TRUSB or TPMB is necessary and target the TRUSB, and strategies starting with TRUSB and using MPMRI to decide whether a repeat biopsy is warranted. A diagnosis of CS cancer requires a biopsy, hence strategies were defined to always end with a confirmatory biopsy. Within each test combination, there are alternative ways each test can be used, following the definitions used in PROMIS (see Table 2, Table 3). Each of the 32 test combinations were tested for the alternative classifications and cut-offs, returning a total of 383 strategies.

**Table 1:** Diagnostic strategies

Test	Strategies
MPMRI	
First test	M1–M7; N1–N7
Second test after TRUSB	T5–T9; P5–P9
TRUSB	
First test	T1–T9; P2–P9
Repeat TRUSB in men with no cancer detected	T2, T4
Repeat TRUSB in men with non-CS cancer detected	T3, T4
Second test after MPMRI: MRI-targeted TRUSB, in men with lesions visible at the MPMRI	M1–M7
Repeat MRI-targeted TRUSB in men with no previous cancer or non-CS cancer at first MRI-targeted TRUSB, but with lesions visible at MRI	M3–M7; T5–T9; N3–N7
TPMB	
First test	P1
Second test	P2–P4; N1–N4
Third test	P5–P9; N3–N7

MPMRI = multiparametric magnetic resonance imaging; TRUSB = transrectal ultrasound-guided biopsy; TPMB = template prostate mapping biopsy; CS = clinically significant. MRI-targeted TRUSB is a TRUSB informed by a prior MPMRI. All TRUSB post-MPMRI are assumed to be MRI-targeted TRUSB.

Diagnostic strategies were labelled according to their test combination first (M1–M7, N1–N7, T1–T9, P1–P9), and then their biopsy TRUSB definition (1 or 2), MPMRI definition (1 or 2), and cut-off (2 to 5). T strategies start with TRUSB, M strategies start with MPMRI, P strategies are the same as T strategies, and N strategies are the same as M strategies but have TPMB as the last biopsy. For example, strategy M1 125 refers to test combination M1, in which all men were first assessed using MPMRI definition 2 and cut-off 5 and then followed up with biopsy definition 1 for those with a suspicion of CS cancer. See the Supplementary material, section 1, for full details on the test sequences for each diagnostic strategy.

**Table 2:** Diagnostic performance of TRUSB

	Subgroups	Low-risk cancer			Intermediate-risk cancer			High-risk cancer			Source
Type	Definition	NC	non-CS	CS	NC	non-CS	CS	NC	non-CS	CS	
1	1	0.65	0.35	0.00	0.24	0.42	0.34	0.00	0.00	1.00	PROMIS [4]
	2	0.65	0.35	0.00	0.24	0.17	0.59	0.00	0.00	1.00	
2	1	0.55	0.45	0.00	0.55	0.25	0.20	0.55	0.00	0.45	[22]
	2	0.55	0.45	0.00	0.55	0.10	0.35	0.55	0.00	0.45	
3	1	0.00	1.00	0.00	0.00	0.75	0.25	0.00	0.75	0.25	[23]
	2	0.00	1.00	0.00	0.00	0.75	0.25	0.00	0.75	0.25	
4	1	0.80	0.20	0.00	0.20	0.37	0.43	0.00	0.00	1.00	PROMIS [4] combined with [16]
	2	0.79	0.21	0.00	0.15	0.11	0.74	0.00	0.00	1.00	
5	1 and 2	0.68	0.32	0.00	0.05	0.08	0.87	0.05	0.08	0.87	[16]

CS = clinically significant; MPMRI = multiparametric magnetic resonance imaging; NC = no cancer; PROMIS = Prostate MR Imaging Study; TPMB = template prostate mapping biopsy; TRUSB = transrectal ultrasound-guided biopsy.

Key:

1: TRUSB before MPMRI

2: TRUSB after a TRUSB that did not detect cancer

3: TRUSB after a TRUSB that detected CNS cancer

4: TRUSB after a suspicious MPMRI

5: TRUSB after a TRUSB that did not detect cancer and a suspicious MPMRI

Parameter inputs are presented as point estimates (mean). See the Supplementary material, section 2, for 95% confidence intervals and details on the data sources.

The diagnostic performance of the first TRUSB (i.e. TRUSB type 1) was obtained from the individual patient data of the PROMIS [4]. For TRUSB and TPMB, the histological CS cancer definitions were (1) dominant Gleason pattern ≥4 and/or any Gleason pattern ≥5 and/or cancer core length ≥6 mm (histology definition 1) and (2) any Gleason pattern ≥4 and/or cancer core length ≥4 mm (histology definition 2). Since the PROMIS collected information on blind first TRUSB, external evidence was used on the sensitivity of repeat TRUSB and MRI-targeted TRUSB, as either first or second TRUSB [16], [22], [23].

**Table 3:** Diagnostic performance of MPMRI

	Subgroups	No cancer			Low-risk cancer			Intermediate-risk cancer			High-risk cancer		
Cut-off	Definition	NC	non-CS	CS	NC	non-CS	CS	NC	non-CS	CS	NC	non-CS	CS
≥2	1	0.00	0.23	0.77	0.00	0.20	0.80	0.01	0.06	0.93	0.00	0.00	1.00
	2	0.00	0.07	0.93	0.00	0.08	0.92	0.01	0.01	0.98	0.00	0.00	1.00
≥3	1	0.33	0.41	0.26	0.28	0.40	0.32	0.08	0.18	0.74	0.00	0.00	1.00
	2	0.33	0.17	0.50	0.28	0.16	0.56	0.08	0.05	0.87	0.00	0.00	1.00
≥4	1	0.86	0.08	0.06	0.75	0.14	0.11	0.30	0.24	0.46	0.00	0.06	0.94
	2	0.86	0.03	0.11	0.75	0.04	0.21	0.30	0.04	0.65	0.00	0.00	1.00
=5	1	0.96	0.02	0.02	0.98	0.01	0.01	0.60	0.17	0.23	0.23	0.16	0.61
	2	0.96	0.01	0.03	0.98	0.00	0.02	0.60	0.03	0.38	0.23	0.00	0.77

CS = clinically significant; MPMRI = multiparametric magnetic resonance imaging; NC = no cancer; PROMIS = Prostate MR Imaging Study.

Parameter inputs are presented as point estimates (mean). See the Supplementary material, section 2, for 95% confidence intervals.

The diagnostic performance of MPMRI was obtained from the individual patient data of the PROMIS [4]. For interpretation of MPMRI, the definitions for CS cancer were a radiologist estimation of (1) lesion volume ≥0.5 cc and/or Gleason score ≥4 + 3, and (2) lesion volume ≥0.2 cc and/or Gleason score ≥3 + 4. Suspicion of a lesion meeting these definitions was scored on a Likert scale (1–5, 1 being highly likely benign and 5 being highly likely malignant). This scale was also used to score the image for whether any cancer (whether considered CS or not) is present.

### 2.2 Model structure

**Fig. 1:**
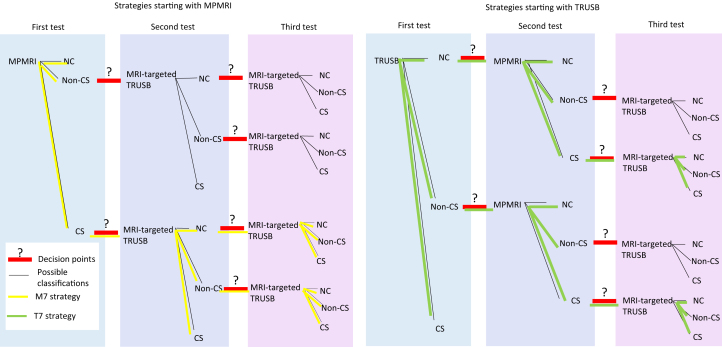
Schematic of decision tree. The diagram represents the decision tree used to predict the outcomes of the diagnostic strategies. The diagram shows only the general structure of the tree for diagnostic strategies composed of MPMRI and TRUSB; a similar tree was used for strategies including TPMB. In the model, men can have a sequence of up to three tests. The black lines represent the possible test classifications. The red lines with a question mark represent decisions. Different decisions constitute different sequences of tests and hence different strategies. The diagram highlights strategies M7 (left side) and T7 (right side). In M7, men receive MPMRI and are classified as having no suspicion of cancer (no cancer; NC), suspicion of non-CS cancer, or suspicion of CS cancer. Men with a suspicion of CS cancer receive an MRI-targeted TRUSB, and are classified as having no cancer (NC), non-CS cancer, and CS cancer. Men in whom CS cancer was not detected, but had a suspicion of CS cancer at the MPMRI, receive a second MRI-targeted biopsy. In T7, men receive a TRUSB, and are classified as having no cancer (NC), non-CS cancer, and CS cancer. Men in whom CS cancer was not detected receive an MPMRI, and are classified as having no suspicion of cancer (NC), suspicion of non-CS cancer, or suspicion of CS cancer. Men classified as having a suspicion of CS cancer based on MPMRI results receive a second TRUSB—this time MRI-targeted TRUSB since there is now information from the MPMRI. CS = clinically significant; MPMRI = multiparametric magnetic resonance imaging; TPMB = template mapping biopsy; TRUSB = transrectal ultrasound-guided biopsy.

The model had a diagnosis and a long-term component (Supplementary Fig. 1). For diagnosis, a decision tree combined the information on diagnostic accuracy of the tests to determine the accuracy of the test combinations (Fig. 1). The long-term outcome component calculated the long-term health outcomes and costs of men with CS cancer, non-CS cancer, and no cancer, by whether they were correctly diagnosed or missed. Their diagnosis determined their clinical management, as either immediate radical treatment if CS cancer is diagnosed or surveillance if not. The long-term outcome component was a cohort Markov, with two health states for men with no cancer (alive and dead) and three states for men with cancer: localised cancer, metastatic cancer, and death. The decision model was developed in Microsoft Excel.

### 2.3 Diagnostic performance

The model explicitly reflects the sensitivity and specificity of TRUSB and MPMRI in detecting prostate cancer. Table 2, Table 3 show the diagnostic performance of the tests, calculated from the individual level data collected in the PROMIS [4] (details in the Supplementary material, section 2). The men's true disease status was classified in four subgroups, according to the TPMB results and their serum prostate-specific antigen (PSA) level [1]:

1. No cancer
2. Low risk: PSA ≤10 ng/ml and Gleason score ≤6, who should be classified as having non-CS cancer
3. Intermediate risk: PSA 10–15 ng/ml or Gleason score 7, who should be classified as having CS cancer
4. High risk: Gleason score ≥8, who should be classified as having CS cancer

### 2.4 Management post diagnosis

The long-term outcomes of men with cancer were based on the Prostate Cancer Intervention Versus Observation Trial (PIVOT) [8], a randomised controlled trial comparing radical prostatectomy and watchful waiting in men with localised prostate cancer, by risk subgroup as defined above. The information from PIVOT was combined with that from the STAMPEDE study (metastatic subgroup) [9] in a calibration model in order to estimate the probability of transition between the Markov model health states. Since the diagnostic strategies are perfectly specific, only men with intermediate- or high-risk cancer are classified as having CS cancer and receive treatment. Details are provided in the Supplementary material, section 3.

### 2.5 Health-related quality of life and costs

For health-related quality of life (HRQoL), the model considers the direct impact of TPMB, obtained from the patient-reported EQ-5D collected in the PROMIS [4]. TRUSB is assumed to have no impact on HRQoL given that no effect was found in a large European screening study [10]. Regarding costs, the model included the direct cost of the tests and the costs associated with managing their related complications [11], [12].

In the long term, the model considers the reduction in HRQoL from any metastatic disease [13] and ageing [14]. The model included the direct cost of radical prostatectomy and surveillance, the costs of their complications, and the costs of metastatic disease [8]. Details are provided in the Supplementary material, sections 4 (HRQoL) and 5 (costs).

### 2.6 Main outcomes and measures

The main outcomes were cost effectiveness of diagnosis, defined as the strategies that detect the most CS cancers for a given pound spent in testing, and long-term cost effectiveness, defined as the strategies that achieve the most health outcomes given their costs, for alternative cost-effectiveness thresholds: £13 000 (€15 398), £20 000 (€23 689), and £30 000 (€35 534)/quality-adjusted life year (QALY) gained [7], [15]. The results are probabilistic in that they are the average of over 1000 Monte Carlo simulations. A number of sensitivity analyses were conducted on the aspects of the short- and long-term components of the model (see the Supplementary material, section 6, for details).

## 3 Results

### 3.1 Base-case analysis

#### 3.1.1 Detection of CS cancers per pound spend in diagnosis

**Fig. 2:**
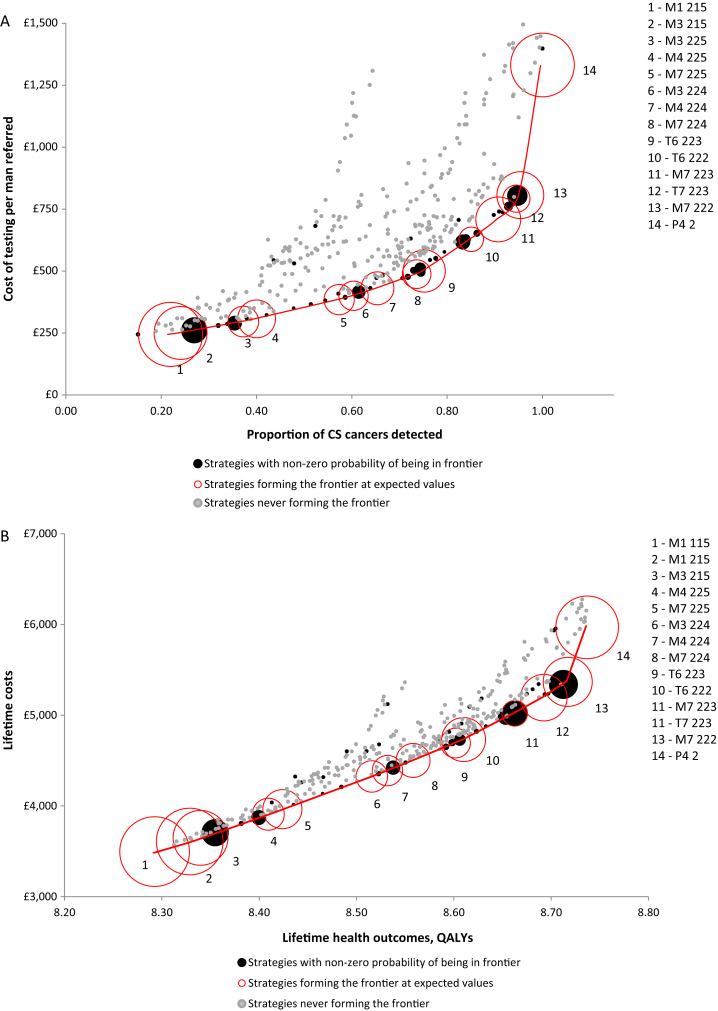
(A) Detection of CS cancers per pound spent in diagnosis. (B) Quality-adjusted life years (QALYs) per NHS spend. Each bubble represents one of the 383 diagnostic strategies evaluated; their size is directly related to the probability that the strategy is cost effective and therefore forms the frontier (ie, forms the red line). The red bubbles represent the 14 diagnostic strategies that form the frontier at expected values. This means that, on average, these are the best strategies per pound spent. The black bubbles represent the strategies that do not form the frontier at expected values, but that have some probability of being in the frontier given their distribution of costs and outcomes. The grey bubbles represent the strategies that do not form the efficiency frontier at any simulation. Given the distribution of parameter inputs, these strategies are never efficient or cost effective. CS = clinically significant; NHS = National Health Service.

Figure 2A plots the detection of CS cancers and cost of testing for each of the 383 strategies defined (see the Supplementary material, section 8, for details, including costs in euro). Out of all the 383 strategies, the figure highlights the 14 strategies that are expected to detect the most CS cancers per pound spent in testing (red circles). These define a frontier of valuable diagnostic options. The remaining strategies are not expected to represent a good value. Owing to the uncertainty around diagnostic accuracy and costs, some of these retain the possibility of being in the frontier, that is, of being valuable (black circles).

Four of 14 red strategies detect at least 80% of the CS cancers: M7 223, T7 223, M7 222, and P4 2 (strategies 10–14 in Fig. 2A). In M7, all men receive MPMRI and men with a suspicion of CS cancer receive an MRI-targeted TRUSB. Men in whom MRI-targeted TRUSB did not detect CS cancer receive a second MRI-targeted TRUSB. M7 223 detects 85% (95% confidence interval [CI] 81–89%) of CS cancers and costs £628 (95% CI £597–660); M7 222 detects 95% (95%CI 92% to 0.98%) and costs £807 (95%CI £777 to £833). This MPMRI definition and cut-off refer to MRI-targeted TRUSB in 96% of men: all men with high-risk CS cancer, 98% with intermediate-risk CS cancer, 92% with low-risk non-CS cancer, and 93% with no cancer. T7 consists of testing all men with TRUSB, followed by MPMRI in men in whom CS cancer was not detected, and a repeat MRI-targeted TRUSB in men with negative TRUSB if there is a suspicion of CS cancer at the MPMRI. T7 223 detects 91% (95% CI 86–94%) CS cancers and costs £709 (95% CI £688–730); P4 2 consists of TRUSB for all men and TPMB for those in whom TRUSB did not detect CS cancer. It has perfect sensitivity but costs £1332 (95% CI £1278–1385).

#### 3.1.2 QALYs per NHS spent

Figure 2B shows the expected lifetime health outcomes and costs achieved by each strategy per man referred for testing (see the Supplementary material, section 9, for details, including costs in euro). The line linking the cost-effective strategies (in red) is the cost-effectiveness frontier, and its slope corresponds to the incremental cost-effectiveness ratio (ICER) of a strategy versus the next best (to its left); the strategies on the frontier and their ICERs are shown in Table 4. The strategy attaining the greatest expected health outcomes was P4 2, and the next best strategy is M7 222. The ICER of P4 2 versus M7 222 was £30 084/QALY. Next best to M7 222 is T7 223, and the ICER of M7 222 versus T7 223 is £7076/QALY gained, making it a cost-effective strategy in the UK setting. These results are consistent with the cost-effectiveness acceptability frontier (Supplementary Fig. 1), in which M7 222 is the strategy most likely to be cost effective for cost-effectiveness thresholds between £7250 and £30 000/QALY.

**Table 4:** Cost-effectiveness results

Strategy	ICER/QALY
M1 115: MPMRI for all men definition 1 cut-off 5; TRUSB in men suspicious of CS cancer definition 1	Reference
M1 215: MPMRI for all men definition 2 cut-off 5; TRUSB in men suspicious of CS cancer definition 1	£3081
M3 215: MPMRI for all men definition 2 cut-off 5; TRUSB in men with suspicion on CS cancer definition 2; men with CNS at first biopsy receive second TRUSB definition 2	£3630
M4 225: MPMRI for all men definition 2 cut-off 5; TRUS-guided in men with suspicion of any cancer definition 2; men with suspicion of CS cancer at MPMRI and in whom CNS cancer was detected at the first biopsy receive second TRUSB definition 2	£3738
M7 225: MPMRI for all men definition 2 cut-off 5; TRUSB definition 2 in men with suspicion of CS cancer; rebiopsy with TRUSB definition 2 in those in whom CS cancer was not detected	£3867
M3 224: MPMRI for all men definition 2 cut-off 4; TRUSB definition 2 in men with suspicion on CS cancer; men with CNS at first biopsy receive second TRUSB definition 2	£3921
M4 224: MPMRI for all men definition 2 cut-off 4; TRUSB definition 2 in men with suspicion of any cancer; men with suspicion of CS cancer at MPMRI and in whom CNS cancer was detected at the first biopsy receive second TRUSB definition 2	£4031
M7 224: MPMRI for all men definition 2 cut-off 4; TRUSB definition 2 in men with suspicion of CS cancer; rebiopsy with TRUSB definition 2 in those in whom CS cancer was not detected but MPMRI had suspicion of CS cancer	£4250
T6 223: TRUSB definition 2 for all men; men classified to have CNS receive an MRI definition 2 cut-off 3; men with suspicion of CS cancer receive a second TRUSB definition 2	£4393
T6 222: TRUSB definition 2 for all men; men classified to have CNS receive an MRI definition 2 cut-off 2; men with suspicion of CS cancer receive a second TRUSB definition 2	£4633
M7 223: MPMRI for all men definition 2 cut-off 3; TRUSB definition 2 in men with suspicion of CS cancer; rebiopsy with TRUSB definition 2 in those in whom CS cancer was not detected	£5501
T7 223: TRUSB definition 2 for all men; men classified to have NC or CNS receive an MPMRI definition 2 cut-off 3; men with suspicion of CS cancer receive a second TRUSB definition 2	£5778
M7 222: MPMRI definition 2 cut-off 2 for all men; TRUSB definition 2 in men with suspicion of CS cancer; rebiopsy with TRUSB definition 2 in those in whom CS cancer was not detected but MPMRI had suspicion of CS cancer	£7076
P4 2: TRUSB definition 2 in all men and TPMB in men in whom CS cancer was not detected	£30 084

CS = clinically significant; ICER = incremental cost-effectiveness ratio; MPMRI = multiparametric magnetic resonance imaging; QALY = quality-adjusted life year. TPMB = template prostate mapping biopsy. TRUSB = transrectal ultrasound-guided biopsy.

The strategies in the cost-effectiveness frontier are shown, together with their ICERs versus the next best strategy. TRUSB after an MPMRI is assumed to be an MRI-targeted TRUSB, as information on the location of the lesion is provided by the MPMRI.

### 3.2 Sensitivity analysis

The cost-effective strategy changed from M7 222 to T7 222, T9 222, or P4 2 in response to a reduction in the sensitivity of MRI-targeted TRUSB and an increase in the sensitivity of the MRI-targeted second TRUSB. The cost-effective strategy changes to P4 2 if the sensitivity of the MRI-targeted second TRUSB reduces, as this does not reduce its the CS cancer detection rates. Increases in the cost of MPMRI coupled with reductions in the cost of TRUSB result in strategies starting with TRUSB becoming cost effective, while reductions in the cost of TPMB favour strategies involving TPMB for all or a large proportion of men. The cost-effective strategy changed to less costly, less sensitive strategies (T7 223 and T6 222) if radical prostatectomy is less cost effective, for example, due to reduced effectiveness, higher HRQoL burden or greater costs. Conversely, the cost-effective strategy changed to more sensitive strategies (P4 2) in men incorrectly classified as no cancer has worse health outcomes. For full results, see the Supplementary material, section 9.

## 4 Discussion

A diagnostic strategy consisting of MPMRI first and up to two MRI-targeted TRUSB at the more sensitive definitions (definition 2) and cut-offs is more likely to be cost effective at cost-effectiveness thresholds at and below £30 000. For MPMRI, this is lesion volume ≥0.2 cc and/or Gleason score ≥3 + 4 (likely benign or above); for TRUSB this is any Gleason pattern ≥4 and/or cancer core length ≥4 mm. The most clinically effective strategy is testing all men with TRUSB at definition 2 and retesting men in whom CS cancer was not detected with TPMB; however, this is not cost effective at current cost-effectiveness thresholds and will not be clinically feasible to deliver across the board in any healthcare setting. These findings can directly inform UK policy, but they can also be generalised to similar, international, settings. The extent to which the cost effectiveness results can be generalised to other jurisdictions depends on the similarities of the population, outcomes, health systems, and pricing.

The sensitivity of MPMRI and TRUSB depends on their definitions and cut-offs. An MPMRI cut-off of 2 and above refers 96% of men to biopsy, but ensures that only 2% of men with intermediate-risk cancer and none of the men with high-risk cancer are missed. Furthermore, it means that most men receive a more sensitive TRUSB, since MRI-targeted TRUSB is thought to be more sensitive than the standard [16]. The recent guidance based on the Prostate Imaging Reporting and Data System (PI-RADS) suggests that men with PI-RADS 1 or 2 should not be referred for biopsy given concerns about overdiagnosis [17]. This may not be equivalent to the cut-off recommended here, since the PROMIS diagnostic study did not use PI-RADS, which is a limitation. Nonetheless, higher MPMRI cut-offs, whilst reducing the proportion of men receiving biopsy, also reduce the proportion of CS cancers detected and treated.

This is the first study comparing all possible ways of using MPMRI, TRUSB, and TPMB to diagnose CS prostate cancer, using data from PROMIS, the largest study on MPMRI and TRUSB [4]. A limitation of PROMIS, and of this study, is that it did not include other tests, such as transperineal biopsies, or the combination of additional clinical and genetic characteristics for diagnosis and risk stratification. This is an area for future research. Another area for future research is the sensitivity of the first and second MRI-targeted TRUSBs, since these parameters were key cost-effectiveness drivers. Previous cost-effectiveness studies compared up to two ways of using MPMRI, either as a first test to determine which men should receive MRI-targeted TRUSB [1], [18], as MRI-targeted TRUSB for all men [1], or for men with previous negative biopsy [19]. For these reasons, this study is the most comprehensive cost-effectiveness analysis to date of alternative diagnostic strategies for prostate cancer.

The appropriate MPMRI cut-off, and ultimately the optimal diagnostic strategy, depends on the cost effectiveness of early diagnosis and treatment. Although this study did not include radiotherapy, it tested the impact of changes on the cost effectiveness of treatment. If cost effectiveness of radiotherapy is similar to or more favourable than that of radical prostatectomy, highly sensitive strategies such as M7 222 are cost effective. Highly sensitive diagnostic strategies may not be cost effective if radical treatment is not as cost effective in the manner modelled here. The cost effectiveness of treatment is less favourable if (1) treatment is less effective, (2) it impacts negatively on HRQoL, or (3) it is costlier than that assumed for this study.

Management of men classified as having no cancer or non-CS cancer also has an impact on the scope for investment in diagnosis. More sensitive monitoring protocols improve the cost effectiveness of less sensitive and less costly diagnostic strategies. There is a dearth of evidence on the effectiveness of repeated testing protocols, which constitutes an important limitation of the current evidence base in support of policy, and meant that these analyses could not formally evaluate the use of such protocols.

In order to evaluate the cost effectiveness of diagnostic tests, evidence is required on the long-term outcomes of patients who are correctly diagnosed and those who are misclassified, given their true disease status. The extensive literature searches conducted for this study did not identify evidence on the outcomes of patients and the effectiveness of treatments when true disease status is known (eg, using TPMB to identify and risk stratify patients). The existing studies used TRUSB to diagnose and risk stratify patients [8], [20], [21]; hence, some individuals may have been underdiagnosed. As a consequence, their long-term quality-adjusted survival may have been overestimated, and the cost effectiveness of treatment may have been underestimated. This issue can only be resolved with better-quality evidence on the outcomes of men with prostate cancer, based on a perfect test such as TPMB for their diagnosis and classification.

## 5 Conclusions

MPMRI is cost effective as the first test for the diagnosis of prostate cancer, when followed by an MRI-targeted TRUSB in men in whom the MPMRI suggests a suspicion for CS cancer, and a second TRUSB if no CS cancer is found, under the most sensitive CS cancer definitions and cut-offs. These findings are sensitive to the cost of each test, sensitivity of MRI-targeted TRUSB, and long-term outcomes of men with cancer, which warrant more empirical research.

  ***Author contributions:*** Rita Faria had full access to all the data in the study and takes responsibility for the integrity of the data and the accuracy of the data analysis.  

*Study concept and design:* All authors.

*Acquisition of data:* All authors.

*Analysis and interpretation of data:* All authors.

*Drafting of the manuscript:* Faria.

*Critical revision of the manuscript for important intellectual content:* All authors.

*Statistical analysis:* Faria, Soares, Spackman.

*Obtaining funding:* Ahmed, Kaplan, Emberton, Sculpher.

*Administrative, technical, or material support:* Faria, Soares.

*Supervision:* Soares, Sculpher.

*Other:* None.  

***Financial disclosures:*** Rita Faria certifies that all conflicts of interest, including specific financial interests and relationships and affiliations relevant to the subject matter or materials discussed in the manuscript (eg, employment/affiliation, grants or funding, consultancies, honoraria, stock ownership or options, expert testimony, royalties, or patents filed, received, or pending), are the following: Rita Faria, Marta O. Soares, Eldon Spackman, Louise Brown, Richard Kaplan, and Mark J. Sculpher have no conflict of interests. Hashim U. Ahmed receives trial funding from Sophiris Biocorp, Trod Medical, and Sonacare Inc., and fees for lectures and proctoring from Sonacare Inc. Mark Emberton receives trial funding from Sophiris Biocorp, Trod Medical, Steba Biotech, Immodulon, and Sonacare Inc.; fees for lectures and proctoring from Sonacare Inc.; and consulting fees from Sonacare Inc., Steba Biotech, and Sophiris Biocorp. Mark Emberton has shares in Nuada Medical Ltd.  

***Funding/Support and role of the sponsor:*** This study was funded by the UK National Institute for Health Research Health Technology Assessment programme. The funders had no role in the design and conduct of the study; collection, management, analysis, and interpretation of the data; preparation, review, or approval of the manuscript; or decision to submit the manuscript for publication. Mark Emberton receives research support from the United Kingdom's National Institute of Health Research (NIHR) UCLH/UCL Biomedical Research Centre. He holds NIHR Senior Investigator status (2015 to date). This work was supported by the Medical Research Council grant reference MC_UU_12023/28.  

***Acknowledgements:*** The authors would like to thank Sarah Willis for useful discussions and sharing of information about the cost-effectiveness modelling in prostate cancer. The authors would also like to thank every man who agreed to take part in the PROMIS.
