# Supplementary material for: Optimising the Diagnosis of Prostate Cancer in the Era of Multiparametric Magnetic Resonance Imaging: A Cost-effectiveness Analysis Based on the Prostate MR Imaging Study (PROMIS)
Source: Eur Urol. 2018 Jan;73(1):23–30. doi: 10.1016/j.eururo.2017.08.018 (PMC5718727; doi:10.1016/j.eururo.2017.08.018)
Supplement: Supplementary file 1 [file mmc1.docx]

**Supplementary material**

**Diagnosing prostate cancer in the era of multi-parametric magnetic resonance imagining: efficiency and cost-effectiveness analysis**

Contents

[1. Model conceptualisation 1](#_Toc492550554)

[1.1 Diagnostic strategies 1](#_Toc492550555)

[1.2 Model 6](#_Toc492550556)

[2. Diagnostic accuracy 6](#_Toc492550557)

[2.1 True disease status 6](#_Toc492550558)

[2.2 TRUSB 7](#_Toc492550559)

[2.3 MPMRI 11](#_Toc492550560)

[3. Calibration for long-term outcomes 12](#_Toc492550561)

[3.1 Step 1: Overall survival curves 12](#_Toc492550562)

[3.2 Step 2: Life years by cancer risk subgroup and management 12](#_Toc492550563)

[3.3 Step 3: Transition probabilities 12](#_Toc492550564)

[4. Health-related quality of life inputs 13](#_Toc492550565)

[5. Resource use and costs 13](#_Toc492550566)

[6. Cost-effectiveness of treatment 15](#_Toc492550567)

[7. Sensitivity analysis 15](#_Toc492550568)

[8. Diagnostic cost-effectiveness results 16](#_Toc492550569)

[9. Cost-effectiveness results 24](#_Toc492550570)

[10. Sensitivity analysis results 32](#_Toc492550571)

[11. References 40](#_Toc492550572)

# Model conceptualisation

# Diagnostic strategies

Supplementary Tables 1–4 represent the 32 tests combinations considered in the study. These test combinations form 383 diagnostic strategies given the two definitions for clinically significant (CS) cancer for TRUSB and MPMRI, and the four cut-offs for MPMRI:

- In M1-M7, all men undergo an MPMRI, which will then inform whether one or two TRUSB are required.
- In N1-N7, all men undergo an MPMRI to inform the need for a TRUSB or TPMB.
- In T1-T9, all men undergo a TRUSB, which can be subsequently followed by an MPMRI and potentially a second TRUSB.
- In P1-P9, all men undergo either a TRUSB or a TPMB, and may have an MPMRI than informs whether to have a second biopsy (TRUSB or TPMB).

Supplementary Table 1 – Diagnostic strategies starting with MPMRI, followed by TRUSB

| **Strategy** | **1^st^ test** | **Results** | **2^nd^ test** | **Results** | **3^rd^ test** | **Results** | **Final** |
| --- | --- | --- | --- | --- | --- | --- | --- |
| M1 | MPMRI | NC |  |  |  |  | NC |
|  |  | NON-CS |  |  |  |  | NC |
|  |  | CS | TRUSB | NC |  |  | NC |
|  |  |  |  | NON-CS |  |  | NON-CS |
|  |  |  |  | CS |  |  | CS |
| M2 | MPMRI | NC |  |  |  |  | NC |
|  |  | NON-CS | TRUSB | NC |  |  | NC |
|  |  |  |  | NON-CS |  |  | NON-CS |
|  |  |  |  | CS |  |  | CS |
|  |  | CS | TRUSB | NC |  |  | NC |
|  |  |  |  | NON-CS |  |  | NON-CS |
|  |  |  |  | CS |  |  | CS |
| M3 | MPMRI | NC |  |  |  |  | NC |
|  |  | NON-CS |  |  |  |  | NON-CS |
|  |  | CS | TRUSB | NC |  |  | NC |
|  |  |  |  | NON-CS | TRUSB | NON-CS | NON-CS |
|  |  |  |  |  |  | CS | CS |
|  |  |  |  | CS |  |  | CS |
| M4 | MPMRI | NC |  |  |  |  | NC |
|  |  | NON-CS | TRUSB | NC |  |  | NC |
|  |  |  |  | NON-CS |  |  | NON-CS |
|  |  |  |  | CS |  |  | CS |
|  |  | CS | TRUSB | NC |  |  | NC |
|  |  |  |  | NON-CS | TRUSB | NON-CS | NON-CS |
|  |  |  |  |  |  | CS | CS |
|  |  |  |  | CS |  |  | CS |
| M5 | MPMRI | NC |  |  |  |  | NC |
|  |  | NON-CS |  |  |  |  | NON-CS |
|  |  | CS | TRUSB | NC | TRUSB | NC | NC |
|  |  |  |  |  |  | NON-CS | NON-CS |
|  |  |  |  |  |  | CS | CS |
|  |  |  |  | NON-CS |  |  | NON-CS |
|  |  |  |  | CS |  |  | CS |
| M6 | MPMRI | NC |  |  |  |  | NC |
|  |  | NON-CS | TRUSB | NC | TRUSB | NC | NC |
|  |  |  |  |  |  | NON-CS | NON-CS |
|  |  |  |  |  |  | CS | CS |
|  |  |  |  | NON-CS |  |  | NON-CS |
|  |  |  |  | CS |  |  | CS |
|  |  | CS | TRUSB | NC | TRUSB | NC | NC |
|  |  |  |  |  |  | NON-CS | NON-CS |
|  |  |  |  |  |  | CS | CS |
|  |  |  |  | NON-CS |  |  | NON-CS |
|  |  |  |  | CS |  |  | CS |
| M7 | MPMRI | NC |  |  |  |  | NC |
|  |  | NON-CS |  |  |  |  | NON-CS |
|  |  | CS | TRUSB | NC | TRUSB | NC | NC |
|  |  |  |  |  |  | NON-CS | NON-CS |
|  |  |  |  |  |  | CS | CS |
|  |  |  |  | NON-CS | TRUSB | NON-CS | NON-CS |
|  |  |  |  |  |  | CS | CS |
|  |  |  |  | CS |  |  | CS |

Supplementary Table 2 – Diagnostic strategies N1-N7: starting with MPMRI, followed by TRUSB and with TPMB as the last biopsy

| **Strategy** | **1^st^ test** | **Results** | **2^nd^ test** | **Results** | **3^rd^ test** | **Results** | **Final** |
| --- | --- | --- | --- | --- | --- | --- | --- |
| N1 | MPMRI | NC |  |  |  |  | NC |
|  |  | NON-CS |  |  |  |  | NON-CS |
|  |  | CS | TPMB | NC |  |  | NC |
|  |  |  |  | NON-CS |  |  | NON-CS |
|  |  |  |  | CS |  |  | CS |
| N2 | MPMRI | NC |  |  |  |  | NC |
|  |  | NON-CS | TPMB | NC |  |  | NC |
|  |  |  |  | NON-CS |  |  | NON-CS |
|  |  |  |  | CS |  |  | CS |
|  |  | CS | TPMB | NC |  |  | NC |
|  |  |  |  | NON-CS |  |  | NON-CS |
|  |  |  |  | CS |  |  | CS |
| N3 | MPMRI | NC |  |  |  |  | NC |
|  |  | NON-CS |  |  |  |  | NON-CS |
|  |  | CS | TRUSB | NC |  |  | NC |
|  |  |  |  | NON-CS | TPMB | NON-CS | NON-CS |
|  |  |  |  |  |  | CS | CS |
|  |  |  |  | CS |  |  | CS |
| N4 | MPMRI | NC |  |  |  |  | NC |
|  |  | NON-CS | TRUSB | NC |  |  | NC |
|  |  |  |  | NON-CS |  |  | NON-CS |
|  |  |  |  | CS |  |  | CS |
|  |  | CS | TRUSB | NC |  |  | NC |
|  |  |  |  | NON-CS | TPMB | NON-CS | NON-CS |
|  |  |  |  |  |  | CS | CS |
|  |  |  |  | CS |  |  | CS |
| N5 | MPMRI | NC |  |  |  |  | NC |
|  |  | NON-CS |  |  |  |  | NON-CS |
|  |  | CS | TRUSB | NC | TPMB | NC | NC |
|  |  |  |  |  |  | NON-CS | NON-CS |
|  |  |  |  |  |  | CS | CS |
|  |  |  |  | NON-CS |  |  | NON-CS |
|  |  |  |  | CS |  |  | CS |
| N6 | MPMRI | NC |  |  |  |  | NC |
|  |  | NON-CS | TRUSB | NC | TPMB | NC | NC |
|  |  |  |  |  |  | NON-CS | NON-CS |
|  |  |  |  |  |  | CS | CS |
|  |  |  |  | NON-CS |  |  | NON-CS |
|  |  |  |  | CS |  |  | CS |
|  |  | CS | TRUSB | NC | TPMB | NC | NC |
|  |  |  |  |  |  | NON-CS | NON-CS |
|  |  |  |  |  |  | CS | CS |
|  |  |  |  | NON-CS |  |  | NON-CS |
|  |  |  |  | CS |  |  | CS |
| N7 | MPMRI | NC |  |  |  |  | NC |
|  |  | NON-CS |  |  |  |  | NON-CS |
|  |  | CS | TRUSB | NC | TPMB | NC | NC |
|  |  |  |  |  |  | NON-CS | NON-CS |
|  |  |  |  |  |  | CS | CS |
|  |  |  |  | NON-CS | TPMB | NON-CS | NON-CS |
|  |  |  |  |  |  | CS | CS |
|  |  |  |  | CS |  |  | CS |

Supplementary Table 3 – Diagnostic strategies T1-T9: starting with TRUSB

| **Strategy** | **1^st^ test** | **Results** | **2^nd^ test** | **Results** | **3^rd^ test** | **Results** | **Final** |
| --- | --- | --- | --- | --- | --- | --- | --- |
| T1 | TRUSB | NC |  |  |  |  | NC |
|  |  | NON-CS |  |  |  |  | NON-CS |
|  |  | CS |  |  |  |  | CS |
| T2 | TRUSB | NC | TRUSB | NC |  |  | NC |
|  |  |  |  | NON-CS |  |  | NON-CS |
|  |  |  |  | CS |  |  | CS |
|  |  | NON-CS |  |  |  |  | NON-CS |
|  |  | CS |  |  |  |  | CS |
| T3 | TRUSB | NC |  |  |  |  | NC |
|  |  | NON-CS | TRUSB | NON-CS |  |  | NON-CS |
|  |  |  |  | CS |  |  | CS |
|  |  | CS |  |  |  |  | CS |
| T4 | TRUSB | NC | TRUSB | NC |  |  | NC |
|  |  |  |  | NON-CS |  |  | NON-CS |
|  |  |  |  | CS |  |  | CS |
|  |  | NON-CS | TRUSB | NON-CS |  |  | NON-CS |
|  |  |  |  | CS |  |  | CS |
|  |  | CS |  |  |  |  | CS |
| T5 | TRUSB | NC | MPMRI | NC |  |  | NC |
|  |  |  |  | NON-CS |  |  | NC |
|  |  |  |  | CS | TRUSB | NC | NC |
|  |  |  |  |  |  | NON-CS | NON-CS |
|  |  |  |  |  |  | CS | CS |
|  |  | NON-CS |  |  |  |  | NON-CS |
|  |  | CS |  |  |  |  | CS |
| T6 | TRUSB | NC |  |  |  |  | NC |
|  |  | NON-CS | MPMRI | NC |  |  | NON-CS |
|  |  |  |  | NON-CS |  |  | NON-CS |
|  |  |  |  | CS | TRUSB | NON-CS | NON-CS |
|  |  |  |  |  |  | CS | CS |
|  |  | CS |  |  |  |  | CS |
| T7 | TRUSB | NC | MPMRI | NC |  |  | NC |
|  |  |  |  | NON-CS |  |  | NC |
|  |  |  |  | CS | TRUSB | NC | NC |
|  |  |  |  |  |  | NON-CS | NON-CS |
|  |  |  |  |  |  | CS | CS |
|  |  | NON-CS | MPMRI | NC |  |  | NC |
|  |  |  |  | NON-CS |  |  | NC |
|  |  |  |  | CS | TRUSB | NON-CS | NON-CS |
|  |  |  |  |  |  | CS | CS |
|  |  | CS |  |  |  |  | CS |
| T8 | TRUSB | NC | MPMRI | NC |  |  | NC |
|  |  |  |  | NON-CS | TRUSB | NC | NC |
|  |  |  |  |  |  | NON-CS | NON-CS |
|  |  |  |  |  |  | CS | CS |
|  |  |  |  | CS | TRUSB | NC | NC |
|  |  |  |  |  |  | NON-CS | NON-CS |
|  |  |  |  |  |  | CS | CS |
|  |  | NON-CS |  |  |  |  | NON-CS |
|  |  | CS |  |  |  |  | CS |
| T9 | TRUSB | NC | MPMRI | NC |  |  | NC |
|  |  |  |  | NON-CS | TRUSB | NC | NC |
|  |  |  |  |  |  | NON-CS | NON-CS |
|  |  |  |  |  |  | CS | CS |
|  |  |  |  | CS | TRUSB | NC | NC |
|  |  |  |  |  |  | NON-CS | NON-CS |
|  |  |  |  |  |  | CS | CS |
|  |  | NON-CS | MPMRI | NC |  |  | NON-CS |
|  |  |  |  | NON-CS |  |  | NON-CS |
|  |  |  |  | CS | TRUSB | NON-CS | NON-CS |
|  |  |  |  |  |  | CS | CS |
|  |  | CS |  |  |  |  | CS |

Supplementary Table 4 – Diagnostic strategies P1-P9: as per T1-T9 but last TRUSB replaced by TPMB

| Strategy | 1st test | Results | 2nd test | Results | 3rd test | Results | Final |
| --- | --- | --- | --- | --- | --- | --- | --- |
| P1 | TPMB | NC |  |  |  |  | NC |
|  |  | NON-CS |  |  |  |  | NON-CS |
|  |  | CS |  |  |  |  | CS |
| P2 | TRUSB | NC | TPMB | NC |  |  | NC |
|  |  |  |  | NON-CS |  |  | NON-CS |
|  |  |  |  | CS |  |  | CS |
|  |  | NON-CS |  |  |  |  | NON-CS |
|  |  | CS |  |  |  |  | CS |
| P3 | TRUSB | NC |  |  |  |  | NC |
|  |  | NON-CS | TPMB | NON-CS |  |  | NON-CS |
|  |  |  |  | CS |  |  | CS |
|  |  | CS |  |  |  |  | CS |
| P4 | TRUSB | NC | TPMB | NC |  |  | NC |
|  |  |  |  | NON-CS |  |  | NON-CS |
|  |  |  |  | CS |  |  | CS |
|  |  | NON-CS | TPMB | NON-CS |  |  | NON-CS |
|  |  |  |  | CS |  |  | CS |
|  |  | CS |  |  |  |  | CS |
| P5 | TRUSB | NC | MPMRI | NC |  |  | NC |
|  |  |  |  | NON-CS |  |  | NC |
|  |  |  |  | CS | TPMB | NC | NC |
|  |  |  |  |  |  | NON-CS | NON-CS |
|  |  |  |  |  |  | CS | CS |
|  |  | NON-CS |  |  |  |  | NON-CS |
|  |  | CS |  |  |  |  | CS |
| P6 | TRUSB | NC |  |  |  |  | NC |
|  |  | NON-CS | MPMRI | NC |  |  | NON-CS |
|  |  |  |  | NON-CS |  |  | NON-CS |
|  |  |  |  | CS | TPMB | NON-CS | NON-CS |
|  |  |  |  |  |  | CS | CS |
|  |  | CS |  |  |  |  | CS |
| P7 | TRUSB | NC | MPMRI | NC |  |  | NC |
|  |  |  |  | NON-CS |  |  | NC |
|  |  |  |  | CS | TPMB | NC | NC |
|  |  |  |  |  |  | NON-CS | NON-CS |
|  |  |  |  |  |  | CS | CS |
|  |  | NON-CS | MPMRI | NC |  |  | NC |
|  |  |  |  | NON-CS |  |  | NC |
|  |  |  |  | CS | TPMB | NON-CS | NON-CS |
|  |  |  |  |  |  | CS | CS |
|  |  | CS |  |  |  |  | CS |
| P8 | TRUSB | NC | MPMRI | NC |  |  | NC |
|  |  |  |  | NON-CS | TPMB | NC | NC |
|  |  |  |  |  |  | NON-CS | NON-CS |
|  |  |  |  |  |  | CS | CS |
|  |  |  |  | CS | TPMB | NC | NC |
|  |  |  |  |  |  | NON-CS | NON-CS |
|  |  |  |  |  |  | CS | CS |
|  |  | NON-CS |  |  |  |  | NON-CS |
|  |  | CS |  |  |  |  | CS |
| P9 | TRUSB | NC | MPMRI | NC |  |  | NC |
|  |  |  |  | NON-CS | TPMB | NC | NC |
|  |  |  |  |  |  | NON-CS | NON-CS |
|  |  |  |  |  |  | CS | CS |
|  |  |  |  | CS | TPMB | NC | NC |
|  |  |  |  |  |  | NON-CS | NON-CS |
|  |  |  |  |  |  | CS | CS |
|  |  | NON-CS | MPMRI | NC |  |  | NON-CS |
|  |  | CS |  |  |  |  | CS |

# Model

Supplementary Fig. 1 – Model schematic


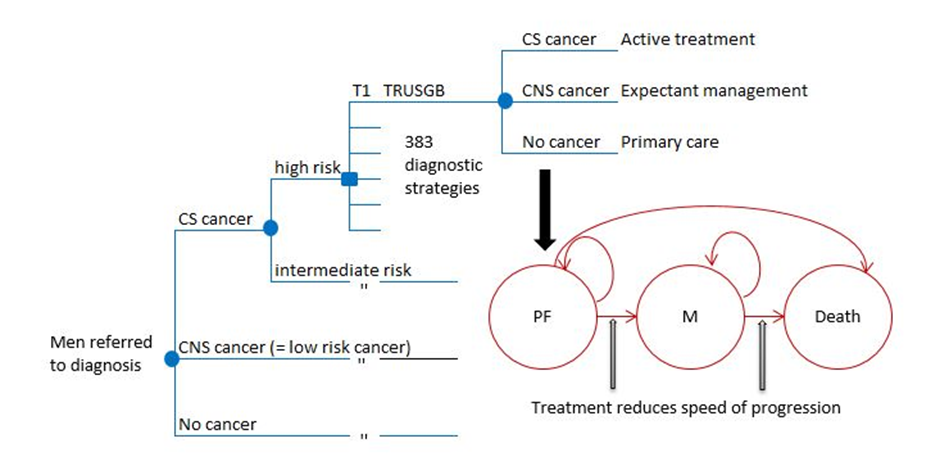


The model included a decision tree for the short-term outcomes of diagnosis and a Markov model for the long-term outcomes of subsequent management decisions. The decision tree is drawn in blue. The circles represent probabilities, and the square a decision. The population has a probability of having clinically significant (CS) cancer, either high risk or intermediate risk cancer, clinically non-significant (non-CS) cancer or no cancer. In order to diagnose disease, men receive one of the 383 diagnostic strategies. The diagram represents T1, in which all men receive transrectal ultrasound guided biopsy (TRUSB) and no more tests. The Markov model is drawn in red. The health states, progression-free (PF) or localised disease, metastatic (M) and dead. The red slim arrows represent the possible transitions.

# Diagnostic accuracy

## True disease status

Firstly, the reference test used here is the combination of TPMB and TRUSB, whatever was most severe. This was required because, in the PROMIS study, TRUSB detected cancer or higher grade cancer in nine men compared with TPMB. In principle, both TPMB and TRUSB are perfectly specific. It is not possible to detect cancer at biopsy if cancer is not present. However, there may be error in TPMB in that a small cancer may have been missed by the sampling grid. Therefore, for the purposes of the economic analysis, we assumed that TPMB and TRUSB were perfectly specific, and that TPMB is perfectly sensitive. As a result, the reference test is the TPMB complemented by TRUSB.

Secondly, seven men were excluded from the analysis since the categories of the PROMIS study would have classified them as CS cancer due to their lesion volume although their Gleason score indicated low risk cancer. This was done in order to match the cancer populations from the PROMIS study with those of the PIVOT study, which used Gleason score to classify patients into levels of risk.

Thirdly, in the PROMIS study, the tests were used to identify CS cancer vs non-CS cancer, which included both men with cancer but not CS cancer and men with no cancer. In clinical practice, men with cancer but not CS cancer and men with no cancer are managed differently. Furthermore, the evidence on long-term outcomes distinguishes between men with CS cancer with intermediate risk or high risk, whom have different outcomes. For these reasons, we divided the cohort into four subgroups: no cancer, non-CS (low risk) cancer, CS (intermediate risk) cancer and CS (high risk) cancer.

Men were classified into four categories as described in Supplementary Table 5.

- No cancer: men with no evidence of cancer at either TPMB or TRUSB.
- Low risk cancer: men with Gleason score < 7 and PSA<10
- Intermediate risk cancer: men with Gleason score=7 or 10≤PSA<20
- High risk cancer: men with Gleason score ≥ 8

Supplementary Table 5 – True disease status

| **Group** | **Definition** | **Number of men** |
| --- | --- | --- |
| No cancer | Men with no evidence of cancer at either TPMB or TRUSB. | 159 |
| Low risk cancer | Men with Gleason score < 7 at either TRUSB or TPMB, and PSA<10. | 91 |
| Intermediate risk cancer | Men with Gleason score=7 either TRUSB or TPMB, or PSA≥10. | 301 |
| High risk cancer | Men with Gleason score ≥ 8 either TRUSB or TPMB. | 18 |

## TRUSB

Supplementary Table 6 summarises the diagnostic performance inputs of each TRUSB and their sources. The model requires information on the diagnostic performance of the six types of TRUSB:

1. TRSUGB 1: first TRUSB without a prior MPMRI. This was obtained from the individual patient data collected in the PROMIS study.
2. TRUSB 2: TRUSB after a TRUSB that did not detect cancer. This was obtained from Roehl et al ^1^ as described below.

Roehl et al report the number of cancers detected in men who underwent series of TRUS-guided biopsies. The total number of cancers detected is used to calculate the prevalence of prostate cancer in the study population as 0.38 (n=963 men with cancer in an initial population N=2,526 men). In the second TRUSB, 143 men were classified as having cancer. Therefore, the probability of detecting cancer given than cancer exists is 0.45. These cancers consist of NON-CS and CS cancers; however, no information is provided on the proportion of NON-CSs compared to CS cancers and how the probability of detection varies by cancer significance. In the absence of this information, it is assumed that the proportion of misclassification is the same as in the firstTRUSB(TRUSB 1) for both intermediate and high risk cancers. In mathematical terms: $\frac{P(\mathrm{TRUSB} 1=NON-CS|int)}{P(\mathrm{TRUSB} 1=CS|int)}=\frac{P(\mathrm{TRUSB} 2=NON-CS|int)}{P(TRSUGB 2=CS|int)}$, where $P(\mathrm{TRUSB} 1=NON-CS|int)$ is the probability of the first TRUSB classifying an individual as having NON-CS given that intermediate risk cancer exists, $P(\mathrm{TRUSB} 1=CS|int)$ is the probability of the first TRUSB classifying an individual as having CS cancer given that intermediate risk cancer exists, $P(\mathrm{TRUSB} 2=NON-CS|int)$ is the probability of second TRUSB classifying an individual as having NON-CS given that intermediate risk cancer exists, $P(\mathrm{TRUSB} 2=CS|int)$ is the probability of the second TRUSB classifying an individual as having CS cancer given that intermediate risk cancer exists.

1. TRUSB 3: TRUSBin men who were classified as non-CS cancer at the first TRUSB. This was obtained from Barzell et al.^2^

Barzell et al compared repeat TRUSB with TPM in men in whom NON-CS had been detected. Five definitions for clinical significance were defined.^3-5^ The University College London (UCL) definitions are the ones that most closely resemble the definitions used in PROMIS and therefore ensure comparability. UCL definition 1 defines CS cancer as localised cancer with a maximum 10mm total core length, maximum 6mm cancer core length and Gleason score at least 4+3 or 3+5. UCL definition 2 defines CS cancer as maximum 6mm total cancer core length, maximum 4mm cancer core length and Gleason score at least 3+4. For definition 1, the probability of TRUSB 3 in detecting CS cancer given that CS cancer is present is 0.24 (10 men tested positive in 41 men with cancer), whilst for definition 2 is 0.25 (17 men tested positive in 69 men with cancer).

1. TRUSB 4: TRUSB after a suspicious MPMRI was obtained by combining the sensitivity of TRUSB 1 in men in whom MPMRI was suspicious from the PROMIS study with the increase in sensitivity reported in Schoots et al. ^6^Schoots et al reports a systematic review and meta-analysis of the sensitivity of MRI-targeted TRUSB compared with blind TRUSB.
2. TRUSB 5: TRUSB after a suspicion MPMRI and no cancer at firstTRUSB was obtained from Schoots et al.^6^

Schoots et al reports the probability of MRI-targeted TRUSB after a previous negative biopsy detecting non-CS and CS cancer ^6^. Since the probability of classifying a CS cancer as non-CS or as NC is not reported, it was assumed that the distribution of intermediate or high risk cancers was the same as in the initial TRUSB. In mathematical terms: $\frac{P(\mathrm{TRUSB} 1=NC|int)}{P(\mathrm{TRUSB} 1=NON-CS|int)}=\frac{P(\mathrm{TRUSB} 5=NC|int)}{P(\mathrm{TRUSB} 5=NON-CS|int)}$, where $P(\mathrm{TRUSB}=NC|int)$ is the probability of the first TRUSB classifying an individual as having no cancer given that intermediate risk cancer exists, $P(\mathrm{TRUSB}=NON-CS|int)$ is the probability of the first TRUSB classifying an individual as having non-CS cancer given that intermediate risk cancer exists, $P(\mathrm{TRUSB} 5=NC|int)$ is the probability of TRUSB 5 classifying an individual as having no cancer given that intermediate risk cancer exists, $P(\mathrm{TRUSB} 5=NON-CS|int)$ is the probability of TRUSB 5 classifying an individual as having non-CS given that intermediate risk cancer exists. In the absence of further evidence, the diagnostic performance of TRUSB 5 in men with intermediate cancer is assumed representative of the performance in men with high risk cancer.

1. TRUSB 6: second TRUSB following a first TRUSB which detected NON-CS and an MPMRI suspicious for cancer. Since no evidence on found on the sensitivity of this TRUSB 6, it was assumed that it was the same as TRUSB 5.

Supplementary Table 6 – Diagnostic performance parameters for TRUSB, mean (95% confidence interval)

| **Test** | **Data source** | **Definition** | **P(NC\|low)** | **P(NON-CS\|low)** | **P(NC\|int)** | **P(NON-CS\|int)** | **P(CS\|int)** | **P(NC\|high)** | **P(NON-CS\|high)** | **P(CS\|high)** |
| --- | --- | --- | --- | --- | --- | --- | --- | --- | --- | --- |
| 1 | PROMIS | 1 | 0.65 (0.55 to 0.75) | 0.35 (0.25 to 0.45) | 0.24 (0.20 to 0.29) | 0.42 (0.36 to 0.47) | 0.34 (0.29 to 0.39) | 0.00 (0.00 to 0.00) | 0.00 (0.00 to 0.00) | 1.00 (1.00 to 1.00) |
|  |  | 2 | 0.65 (0.55 to 0.75) | 0.35 (0.25 to 0.45) | 0.24 (0.20 to 0.29) | 0.17 (0.13 to 0.21) | 0.59 (0.54 to 0.64) | 0.00 (0.00 to 0.00) | 0.00 (0.00 to 0.00) | 1.00 (1.00 to 1.00) |
| 2 | Roehl et al ^1^ | 1 | 0.55 (0.53 to 0.57) | 0.45 (0.43 to 0.47) | 0.55 (0.53 to 0.57) | 0.25 (0.22 to 0.28) | 0.20 (0.17 to 0.23) | 0.55 (0.53 to 0.57) | 0.00 (0.00 to 0.00) | 0.45 (0.43 to 0.47) |
|  |  | 2 | 0.55 (0.53 to 0.57) | 0.45 (0.43 to 0.47) | 0.55 (0.53 to 0.57) | 0.10 (0.07 to 0.13) | 0.35 (0.32 to 0.38) | 0.55 (0.53 to 0.57) | 0.00 (0.00 to 0.00) | 0.45 (0.43 to 0.47) |
| 3 | Batrzell et al ^2^ | 1 | 0.00 (0.00 to 0.00) | 1.00 (1.00 to 1.00) | 0.00 (0.00 to 0.00) | 0.75 (0.55 to 0.88) | 0.25 (0.12 to 0.45) | 0.00 (0.00 to 0.00) | 0.75 (0.55 to 0.88) | 0.25 (0.12 to 0.45) |
|  |  | 2 | 0.00 (0.00 to 0.00) | 1.00 (1.00 to 1.00) | 0.00 (0.00 to 0.00) | 0.75 (0.63 to 0.85) | 0.25 (0.15 to 0.37) | 0.00 (0.00 to 0.00) | 0.75 (0.63 to 0.85) | 0.25 (0.15 to 0.37) |
| 4 | PROMIS and  Schoots et al ^6^ | 1 | 0.80 (0.67 to 0.89) | 0.20 (0.11 to 0.33) | 0.20 (0.15 to 0.24) | 0.37 (0.31 to 0.44) | 0.43 (0.36 to 0.52) | 0.00 (0.00 to 0.00) | 0.00 (0.00 to 0.00) | 1.00 (1.00 to 1.00) |
|  |  | 2 | 0.79 (0.66 to 0.89) | 0.21 (0.11 to 0.34) | 0.15 (0.09 to 0.21) | 0.11 (0.06 to 0.16) | 0.74 (0.65 to 0.84) | 0.00 (0.00 to 0.00) | 0.00 (0.00 to 0.00) | 1.00 (1.00 to 1.00) |
| 5 | Schoots et al ^6^ | Same for both definitions | 0.68 (0.02 to 1.00) | 0.32 (0.02 to 0.91) | 0.05 (0.02 to 0.11) | 0.08 (0.03 to 0.18) | 0.87 (0.71 to 0.95) | 0.05 (0.02 to 0.11) | 0.08 (0.03 to 0.18) | 0.87 (0.71 to 0.95) |

P(NC|NC) - probability of testing no cancer given that no cancer exists - is 1 since TRUSB is a perfectly specific test. It can only detect cancer if cancer exists. Therefore, no cancer can be detected if cancer does not exist.

Key:

1: the first biopsy in all men referred to secondary care.

2: the second biopsy in men whose first TRUSB did not detect cancer.

3: the second biopsy in men whose first TRUSB detected NON-CS.

4: the first biopsy following a suspicious MPMRI for cancer.

5: the second biopsy following a first biopsy which did not detect cancer and an MPMRI suspicious for cancer.

6: the second biopsy following a first biopsy which detected NON-CS and an MPMRI suspicious for cancer.

P(NC|low): probability of testing no cancer given that low risk cancer exists.

P(NON-CS|low): probability of testing non-CS given that low risk cancer exists.

P(NC|int): probability of testing no cancer given that intermediate risk cancer exists.

P(NON-CS|int): probability of testing non-CS given that intermediate risk cancer exists.

P(CS|int): probability of testing CS cancer given that intermediate risk cancer exists.

P(NC|high): probability of testing no cancer given that high risk cancer exists.

P(NON-CS|high): probability of testing non-CS given that high risk cancer exists.

P(CS|high): probability of testing CS cancer given that high risk cancer exists.

## MPMRI

The diagnostic performance of MPMRI is obtained from the data collected in PROMIS. In PROMIS, lesions were classified in degree of suspicion for any cancer and CS cancer using the suspicion scores. Supplementary Table 7 presents the diagnostic performance of MPMRI for different definitions and cut-offs.

Supplementary Table 7 – Diagnostic performance of MPMRI, mean (95% confidence interval)

|  | **MPMRI classification** | | |
| --- | --- | --- | --- |
| **Disease status** | **No suspicion of cancer** | **Suspicion of NON-CS** | **Suspicion of CS cancer** |
| Definition 2 cut-off 2 | | | |
| No cancer | 0.00 (0.00 to 0.00) | 0.07 (0.03 to 0.11) | 0.93 (0.89 to 0.97) |
| Low risk cancer | 0.00 (0.00 to 0.00) | 0.08 (0.02 to 0.14) | 0.92 (0.86 to 0.98) |
| Intermediate risk cancer | 0.01 (0.00 to 0.02) | 0.01 (0.00 to 0.02) | 0.98 (0.97 to 1.00) |
| High risk cancer | 0.00 (0.00 to 0.00) | 0.00 (0.00 to 0.00) | 1.00 (1.00 to 1.00) |
| Definition 2 cut-off 3 |  |  |  |
| No cancer | 0.33 (0.26 to 0.40) | 0.17 (0.11 to 0.23) | 0.50 (0.43 to 0.58) |
| Low risk cancer | 0.28 (0.19 to 0.38) | 0.16 (0.08 to 0.24) | 0.56 (0.46 to 0.67) |
| Intermediate risk cancer | 0.08 (0.05 to 0.11) | 0.05 (0.02 to 0.07) | 0.87 (0.83 to 0.91) |
| High risk cancer | 0.00 (0.00 to 0.00) | 0.00 (0.00 to 0.00) | 1.00 (1.00 to 1.00) |
| Definition 2 cut-off 4 |  |  |  |
| No cancer | 0.86 (0.80 to 0.91) | 0.03 (0.01 to 0.05) | 0.11 (0.06 to 0.17) |
| Low risk cancer | 0.75 (0.66 to 0.84) | 0.04 (0.01 to 0.09) | 0.21 (0.13 to 0.29) |
| Intermediate risk cancer | 0.30 (0.25 to 0.35) | 0.04 (0.02 to 0.07) | 0.65 (0.60 to 0.71) |
| High risk cancer | 0.00 (0.00 to 0.00) | 0.00 (0.00 to 0.00) | 1.00 (1.00 to 1.00) |
| Definition 2 cut-off 5 |  |  |  |
| No cancer | 0.96 (0.93 to 0.99) | 0.01 (0.00 to 0.02) | 0.03 (0.01 to 0.06) |
| Low risk cancer | 0.98 (0.94 to 1.00) | 0.00 (0.00 to 0.00) | 0.02 (0.00 to 0.06) |
| Intermediate risk cancer | 0.60 (0.54 to 0.65) | 0.03 (0.01 to 0.05) | 0.38 (0.32 to 0.44) |
| High risk cancer | 0.23 (0.04 to 0.45) | 0.00 (0.00 to 0.00) | 0.77 (0.55 to 0.96) |
| Definition 1 cut-off 2 |  |  |  |
| No cancer | 0.00 (0.00 to 0.00) | 0.23 (0.17 to 0.29) | 0.77 (0.71 to 0.83) |
| Low risk cancer | 0.00 (0.00 to 0.00) | 0.20 (0.12 to 0.29) | 0.80 (0.71 to 0.88) |
| Intermediate risk cancer | 0.01 (0.00 to 0.02) | 0.06 (0.03 to 0.09) | 0.93 (0.90 to 0.96) |
| High risk cancer | 0.00 (0.00 to 0.00) | 0.00 (0.00 to 0.00) | 1.00 (1.00 to 1.00) |
| Definition 1 cut-off 3 |  |  |  |
| No cancer | 0.33 (0.26 to 0.40) | 0.41 (0.33 to 0.49) | 0.26 (0.19 to 0.33) |
| Low risk cancer | 0.28 (0.19 to 0.38) | 0.40 (0.30 to 0.50) | 0.32 (0.22 to 0.41) |
| Intermediate risk cancer | 0.08 (0.05 to 0.11) | 0.18 (0.13 to 0.22) | 0.74 (0.69 to 0.79) |
| High risk cancer | 0.00 (0.00 to 0.00) | 0.00 (0.00 to 0.00) | 1.00 (1.00 to 1.00) |
| Definition 1 cut-off 4 |  |  |  |
| No cancer | 0.86 (0.80 to 0.91) | 0.08 (0.04 to 0.13) | 0.06 (0.02 to 0.10) |
| Low risk cancer | 0.75 (0.66 to 0.84) | 0.14 (0.08 to 0.22) | 0.11 (0.05 to 0.18) |
| Intermediate risk cancer | 0.30 (0.25 to 0.35) | 0.24 (0.19 to 0.28) | 0.46 (0.41 to 0.52) |
| High risk cancer | 0.00 (0.00 to 0.00) | 0.06 (0.00 to 0.18) | 0.94 (0.82 to 1.00) |
| Definition 1 cut-off 5 |  |  |  |
| No cancer | 0.96 (0.93 to 0.99) | 0.02 (0.00 to 0.04) | 0.02 (0.00 to 0.05) |
| Low risk cancer | 0.98 (0.94 to 1.00) | 0.01 (0.00 to 0.04) | 0.01 (0.00 to 0.04) |
| Intermediate risk cancer | 0.60 (0.54 to 0.65) | 0.17 (0.13 to 0.22) | 0.23 (0.18 to 0.28) |
| High risk cancer | 0.23 (0.04 to 0.45) | 0.16 (0.00 to 0.35) | 0.61 (0.38 to 0.84) |

# Calibration for long-term outcomes

# Step 1: Overall survival curves

The survival curves reported in the Supplementary Material of Wilt et al for each subgroup allocated to watchful waiting were digitised using WebPlotDigitizer, a free online tool. Alternative parametric distributions were assessed for goodness of fit with the available data. The Weibull distribution was selected because it was the parametric model that best fitted the observed data across the three cancer risk subgroups. In the extrapolation period, the Weibull distribution predicted lower hazards of death than those of the general population. Therefore, at relevant times, the hazard of death of the general population was thus used to predict survival.

# Step 2: Life years by cancer risk subgroup and management

The survival predicted by the Weibull model as described in Step 1 was used to calculate life expectancy from diagnosis by cancer risk subgroup and management. Supplementary Table 8 shows the Weibull model parameters and life expectancy calculated from Steps 1 and 2.

Supplementary Table 8 – Weibull model parameters and life expectancy, mean (95% confidence intervals)

| **Subgroup** | **Management** | **Scale** | **Shape** | **Life years** |
| --- | --- | --- | --- | --- |
| Low risk cancer | Watchful waiting | 0.019 (0.015 to 0.023) | 1.278 (1.248 to 1.306) | 16.26 (14.77 to 17.26) |
| Intermediate risk cancer | Watchful waiting | 0.025 (0.022 to 0.029) | 1.358 (1.312 to 1.403) | 13.38 (11.59 to 15.09) |
|  | Radical prostatectomy | 0.018 (0.015 to 0.021) |  | 15.65 (13.95 to 16.93) |
| High risk cancer | Watchful waiting | 0.027 (0.021 to 0.034) | 1.463 (1.436 to 1.489) | 11.15 (9.16 to 13.47) |
|  | Radical prostatectomy | 0.020 (0.015 to 0.025) |  | 13.23 (10.91 to 15.42) |

# Step 3: Transition probabilities

The transition probabilities were obtained with a Markov model with three health states (progression-free, metastatic and death), evaluated by calibration. The model was calibrated to the life expectancyshown in Supplementary Table 8, the proportion of patients metastasised in Wilt,^7^ and a plausible range of values for the probability of dying after metastasis from the standard care arm of STAMPEDE trial^8^ STAMPEDE is an RCT set in the UK comparing treatments for men with locally advanced and metastatic prostate cancer.^8^

The calibration model randomly draws numbers between 0 and 1 from the transitions from progression-free to metastatic and death. For the transition from metastatic to death, the model draws a random number between the 95% confidence interval of the cumulative incidence of all-cause death from the metastatic subgroup allocated to the standard care arm of STAMPEDE trial.^8^. A 10% leeway is given to allow for differences in the severity of patients and their management between STAMPEDE and PIVOT.^7,8^

The calibration model records the transition probabilities that meet three conditions of plausibility: (1) the life years are within the 95% confidence interval of predicted in Step 1; (2) the cumulative incidence of metastases at 12 years is within the 95% confidence interval of the PIVOT trial and (3) the transition probability from progression-free to death is smaller than the transition probability from metastatic to death. The calibration model was run until 1,000 plausible sets of transition probabilities for each subgroup were found. Supplementary Table 9 shows the transition probabilities obtained from this method.

Supplementary Table 9 – Transition probabilities predicted by the calibration model, mean (95% confidence intervals)

| **Subgroup** | **Management** | **Transition probabilities** | | |
| --- | --- | --- | --- | --- |
|  |  | **Progression free to metastases** | **Progression free to death** | **Metastases to death** |
| Low risk cancer | Watchful waiting | 0.008 (0.004 to 0.013) | 0.050 (0.043 to 0.058) | 0.139 (0.058 to 0.226) |
| Intermediate risk cancer | Watchful waiting | 0.018 (0.010 to 0.026) | 0.064 (0.049 to 0.078) | 0.145 (0.071 to 0.223) |
|  | Radical prostatectomy | 0.007 (0.003 to 0.011) | 0.054 (0.045 to 0.063) | 0.142 (0.062 to 0.226) |
| High risk cancer | Watchful waiting | 0.022 (0.011 to 0.034) | 0.080 (0.058 to 0.101) | 0.157 (0.087 to 0.226) |
|  | Radical prostatectomy | 0.008 (0.002 to 0.014) | 0.070 (0.053 to 0.085) | 0.148 (0.071 to 0.225) |

# Health-related quality of life inputs

Supplementary Table 10 shows the health-related quality of life inputs and their sources used in the analysis.

Supplementary Table 10 – Health-related quality of life inputs, average (95% confidence interval)

| **Parameter** | **Value** | **Source or rationale** |
| --- | --- | --- |
| In the short-term | | |
| Change post-MPMRI | Assumed zero | The change post-MPMRI in PROMIS was negligible. |
| Change post-TRUSB | Assumed zero | Essink-Bot et al found no impact of TRUSB on health-related quality of life ^9^. |
| Change post-TPM biopsy | -0.176 (-0.15 to -0.20) | Obtained from the PROMIS IPD.  Duration of decrement assumed to be 2 weeks based on Merrick et al and Tsivian et al ^10^. |
| In the long-term | | |
| Decrement from metastatic disease | -0.137 | Torvinen et al ^11^  Calculated as the difference between the average EQ-5D score reported for localised cancer and the EQ-5D score reported for metastatic cancer. The probabilistic value is sampled from the difference between the distributions (parameterised as beta distributions) |
| Age-related decrement  constant  coefficient on male  coefficient on age  coefficient on age squared | 0.9508566  0.0212126  -0.0002587  -0.0000332 | Ara et al ^12^ |

# Resource use and costs

Supplementary Table 11 shows the resource use inputs and their sources used in the analysis.

Supplementary Table 11 – Resource use in the short-term model

| **Parameter** | **Value** | **Source** |
| --- | --- | --- |
| Associated with TRUSB |  |  |
| Hospital admissions | 1.3% (15/1147) | Rosario et al, 2012 |
| Consultation with general practitioner | 1.2% (14/1147) |  |
| Urology department nurse | 1.2% (14/1147) |  |
| Another source of medical advice | 1.1% (13/1147) |  |
| Associated with TPM biopsy |  |  |
| Hospital admissions | 1.6% (10/630) | Pepe &Aragona |
| Accident & Emergency admission | 14.4% (91/630) |  |
| Incidence of adverse events | | |
| Following radical prostatectomy  Sexual dysfunction  Urinary incontinence  Bowel dysfunction | 231/285  49/287  35/286 | Wilt et al ^7^  Incidence over 2 years, converted to one year probability. |
| Following watchful waiting  Sexual dysfunction  Urinary incontinence  Bowel dysfunction | 124/281  18/284  32/282 | Wilt et al ^7^  Incidence over 2 years, converted to one year probability. |

Supplementary Table 12 shows the unit costs used in the analysis. The unit costs are those relevant to the UK NHS, and were converted to euro using the rate valid on 03/05/2017 (1 GPB=1.18 EUR) and rounded to the nearest unit.

Supplementary Table 12 – Unit costs

| Parameter | Value (€) | Source |
| --- | --- | --- |
| Diagnostic tests | | |
| TRUSB | £403 (477) | LB76Z Transrectal ultrasound guided biopsy of the prostate^13^ |
| TPM biopsy | £1,370 (1,623) | LB77Z Transperineal template biopsy of prostate^13^ |
| MPMRI T2 DW DCE | £182 (216) | RD03Z MRI scan of one area with pre- and post-contrast ^13^ |
| Management of adverse events from testing | | |
| Accident and emergency admission | £132 (156) | Average cost of emergency medicine. ^13^ |
| Hospital admission | £587 (695) | Average cost of non-elective admission, short stay. ^13^ |
| Urology department nurse | £94 (111) | Unit cost of WF01A: non-admitted face to face attendance, follow-up, Urology^13^ |
| Other costs | | |
| General practitioner | £44 (52) | Curtis et al^14^ |
| Other health care advice | £7.9 (9) | Curtis et al^14^ |
| Costs of long-term | | |
| Cost of radical prostatectomy  Surgery cost  Urology follow-up  First surgical consultation  Follow-up surgical consultation | £4,667=  £4,275 (5064)+  £91 (108)+  £210 (249)+  £91 (108)+ | Average between the PbR tariff between LB21Z Bladder Neck Open Procedures - Male and LB22Z Laparoscopic Bladder Neck Procedures - Male ^15^  PbR tariff for WF01A: Follow-up attendance single-professional ^15^  PbR tariff for WD01B: First attendance single-professional ^15^  PbR tariff for WF01A: Follow-up attendance single-professional ^15^ |
| Cost of watchful waiting (per year) | £138=  £91 (108)+  £16 (19) | PbR tariff for WF01A: Follow-up attendance single-professional ^15^  Cost of PSA test in primary care, conducted three times per year^15^ |
| Cost of adverse events (per year)  Sexual dysfunction  Urinary incontinence  Bowel dysfunction | £205=  £282 (334)+  £279 (330)+  £1,707 (2022) | Weighted average of the cost of managing adverse events by the incidence of each adverse event.  PbR tariff for LB43Z: Treatment of erectile dysfunction  Management by containment pads, from NICE Clinical Guideline 2014 inflated from 2008-09 to 2014-15 ^14,16^  Mean weighted cost of sigmoidoscopy, laser therapy, enemas and blood transfusion, from NICE Clinical Guideline 2014, inflated from 2008-09 to 2014-15 ^14,16^ |
| Cost of metastatic cancer per year | £1,876 (2222) | Calculated from Lord et al (605.45 years of life predicted from the end of progression-free to death+39.92 years from 4th line palliative care treatment to chemotherapy; the lifetime cost of treatment is £1,072,554 (price year 2010-11), which inflated to 2014-15 is £1,136,124 ^14,17^ |

# Cost-effectiveness of treatment

Supplementary Table 13 shows the lifetime QALYs and costs, discounted at 3.5%, predicted using the plausible set of transition probabilities. The incremental cost-effectiveness ratio (ICER) of radical prostatectomy vs watchful waiting in men with intermediate risk (CS) cancer is £3,067 per QALY gained. The probability that radical prostatectomy is cost-effective is 0.91 assuming that the health opportunity costs (i.e. cost-effectiveness threshold) is £13,000 per QALY gained; it is 0.93 for health opportunity costs of £20,000 per QALY gained and 0.94 for health opportunity costs of £30,000 per QALY gained.

The ICER of radical prostatectomy vs watchful waiting in men with high risk (CS) cancer is £3,602 per QALY gained. The probability that radical prostatectomy is cost-effective is 0.78 for health opportunity costs of £13,000 per QALY gained; it is 0.81 for health opportunity costs of £20,000 per QALY gained, and 0.82 for health opportunity costs of £30,000 per QALY gained. The implication is that radical prostatectomy is highly likely to be cost-effective compared with watchful waiting.

Supplementary Table 13 – Lifetime health outcomes and costs (95% CI)

| **Subgroups** | **Management** | **Lifetime QALYs** | **Lifetime costs (£)** | **Lifetime costs (€)** |
| --- | --- | --- | --- | --- |
| Low risk cancer | Watchful waiting | 8.45 (7.99 to 8.94) | £3,994 (£3,301 to £4,894) | 4,731 (3,910 to 5,797) |
| Intermediate risk cancer | Watchful waiting | 7.29 (6.65 to 8.03) | £4,130 (£3,215 to £5,351) | 4,891 (3,808 to 6,338) |
|  | Radical prostatectomy | 8.23 (7.69 to 8.79) | £7,041 (£6,353 to £7,959) | 8,340 (7,524 to 9,427) |
| High risk cancer | Watchful waiting | 6.38 (5.59 to 7.36) | £3,764 (£2,804 to £5,001) | 4,458 (3,321 to 5,923) |
|  | Radical prostatectomy | 7.21 (6.42 to 8.18) | £6,796 (£6,112 to £7,746) | 8,050 (7,239 to 9,175) |

# Sensitivity analysis

The sensitivity analysis consisted of:

1. Bivariate sensitivity analysis on the unit cost of tests
2. Scenario using Payment by Results tariff for the unit cost of tests
3. Threshold sensitivity analysis (TSA):
   - TSA1: Relative sensitivity of MRI-targeted TRUSB in detecting CS cancer.
   - TSA2: Relative sensitivity of MRI-targeted secondTRUSB in detecting CS cancer
   - TSA3: Prevalence of intermediate risk vs low risk cancer
   - TSA4: Probability of no cancer
   - TSA5: Risk of death from TRUSB
   - TSA6: Reduced quality-adjusted survival from incorrect classification as no cancer
   - TSA7: Reduced effectiveness of radical prostatectomy
   - TSA8: Impact of repeated testing over time
4. Value of future research^18^

# Diagnostic cost-effectiveness results

Supplementary Tables 13 and 14 show the proportion of CS cancers detected by strategy, and Supplementary Tables 15 and 16 (and 17 and 18 in euro) show the cost of testing. Supplementary Table 19 shows the strategies forming the efficiency frontier at their average values.

Supplementary Table 14 – Proportion of CS cancers detected by strategy, mean (95% confidence intervals), TRUSGG definition 1, MPMRI definition 1 and 2, MPMRI cut-off 2-5

| Strategy | 112 | 113 | 114 | 115 | 122 | 123 | 124 | 125 |
| --- | --- | --- | --- | --- | --- | --- | --- | --- |
| M1 | 0.44 (0.36 to 0.51) | 0.36 (0.30 to 0.42) | 0.24 (0.20 to 0.29) | 0.15 (0.12 to 0.19) | 0.46 (0.38 to 0.53) | 0.41 (0.35 to 0.48) | 0.32 (0.27 to 0.38) | 0.21 (0.17 to 0.25) |
| M2 | 0.46 (0.39 to 0.54) | 0.43 (0.36 to 0.50) | 0.34 (0.29 to 0.40) | 0.22 (0.18 to 0.27) | 0.46 (0.39 to 0.54) | 0.43 (0.36 to 0.50) | 0.34 (0.29 to 0.40) | 0.22 (0.18 to 0.27) |
| M3 | 0.72 (0.66 to 0.78) | 0.58 (0.52 to 0.64) | 0.39 (0.34 to 0.44) | 0.22 (0.18 to 0.27) | 0.76 (0.70 to 0.81) | 0.68 (0.62 to 0.73) | 0.52 (0.47 to 0.58) | 0.33 (0.28 to 0.38) |
| M4 | 0.75 (0.68 to 0.80) | 0.66 (0.59 to 0.71) | 0.48 (0.43 to 0.54) | 0.29 (0.25 to 0.34) | 0.76 (0.70 to 0.82) | 0.70 (0.64 to 0.75) | 0.54 (0.48 to 0.60) | 0.34 (0.29 to 0.39) |
| M5 | 0.59 (0.52 to 0.65) | 0.48 (0.42 to 0.54) | 0.32 (0.27 to 0.37) | 0.19 (0.15 to 0.23) | 0.61 (0.55 to 0.68) | 0.55 (0.49 to 0.61) | 0.43 (0.37 to 0.48) | 0.27 (0.23 to 0.31) |
| M6 | 0.62 (0.55 to 0.68) | 0.58 (0.52 to 0.64) | 0.45 (0.40 to 0.51) | 0.29 (0.24 to 0.33) | 0.62 (0.55 to 0.68) | 0.58 (0.52 to 0.64) | 0.45 (0.40 to 0.51) | 0.29 (0.24 to 0.33) |
| M7 | 0.87 (0.81 to 0.93) | 0.70 (0.64 to 0.76) | 0.46 (0.40 to 0.52) | 0.26 (0.21 to 0.31) | 0.92 (0.85 to 0.97) | 0.82 (0.75 to 0.87) | 0.63 (0.57 to 0.69) | 0.39 (0.33 to 0.44) |
| N1 | 0.94 (0.91 to 0.96) | 0.75 (0.70 to 0.80) | 0.49 (0.44 to 0.55) | 0.28 (0.23 to 0.33) | 0.98 (0.97 to 1.00) | 0.88 (0.84 to 0.91) | 0.67 (0.62 to 0.73) | 0.41 (0.36 to 0.47) |
| N2 | 0.99 (0.98 to 1.00) | 0.92 (0.89 to 0.95) | 0.71 (0.66 to 0.77) | 0.44 (0.38 to 0.49) | 0.99 (0.98 to 1.00) | 0.92 (0.89 to 0.95) | 0.71 (0.66 to 0.77) | 0.44 (0.38 to 0.49) |
| N3 | 0.77 (0.72 to 0.81) | 0.62 (0.56 to 0.67) | 0.41 (0.36 to 0.46) | 0.23 (0.19 to 0.28) | 0.80 (0.76 to 0.85) | 0.72 (0.67 to 0.76) | 0.55 (0.50 to 0.61) | 0.34 (0.29 to 0.39) |
| N4 | 0.79 (0.74 to 0.83) | 0.69 (0.64 to 0.74) | 0.50 (0.45 to 0.56) | 0.30 (0.26 to 0.35) | 0.81 (0.76 to 0.85) | 0.74 (0.69 to 0.78) | 0.57 (0.52 to 0.63) | 0.35 (0.30 to 0.41) |
| N5 | 0.61 (0.55 to 0.67) | 0.49 (0.44 to 0.55) | 0.33 (0.29 to 0.38) | 0.19 (0.16 to 0.23) | 0.64 (0.57 to 0.70) | 0.57 (0.51 to 0.63) | 0.44 (0.39 to 0.50) | 0.28 (0.24 to 0.32) |
| N6 | 0.64 (0.58 to 0.70) | 0.60 (0.54 to 0.66) | 0.47 (0.42 to 0.53) | 0.29 (0.25 to 0.34) | 0.64 (0.58 to 0.70) | 0.60 (0.54 to 0.66) | 0.47 (0.42 to 0.53) | 0.29 (0.25 to 0.34) |
| N7 | 0.94 (0.91 to 0.96) | 0.75 (0.70 to 0.80) | 0.49 (0.44 to 0.55) | 0.28 (0.23 to 0.33) | 0.98 (0.97 to 1.00) | 0.88 (0.84 to 0.91) | 0.67 (0.62 to 0.73) | 0.41 (0.36 to 0.47) |
| T1 | 0.38 (0.33 to 0.43) | (1) | (1) | (1) | (1) | (1) | (1) | (1) |
| T2 | 0.42 (0.37 to 0.48) | (1) | (1) | (1) | (1) | (1) | (1) | (1) |
| T3 | 0.48 (0.41 to 0.56) | (1) | (1) | (1) | (1) | (1) | (1) | (1) |
| T4 | 0.52 (0.45 to 0.61) | (1) | (1) | (1) | (1) | (1) | (1) | (1) |
| T5 | 0.56 (0.51 to 0.62) | 0.52 (0.47 to 0.58) | 0.47 (0.42 to 0.52) | 0.42 (0.38 to 0.48) | 0.57 (0.52 to 0.63) | 0.55 (0.50 to 0.61) | 0.51 (0.46 to 0.56) | 0.45 (0.40 to 0.50) |
| T6 | 0.70 (0.64 to 0.75) | 0.63 (0.58 to 0.69) | 0.54 (0.49 to 0.59) | 0.46 (0.41 to 0.51) | 0.72 (0.66 to 0.77) | 0.68 (0.62 to 0.73) | 0.60 (0.55 to 0.66) | 0.51 (0.46 to 0.56) |
| T7 | 0.89 (0.81 to 0.94) | 0.78 (0.71 to 0.84) | 0.63 (0.57 to 0.68) | 0.51 (0.45 to 0.56) | 0.91 (0.84 to 0.97) | 0.85 (0.78 to 0.91) | 0.73 (0.67 to 0.79) | 0.58 (0.53 to 0.64) |
| T8 | 0.57 (0.52 to 0.63) | 0.56 (0.51 to 0.62) | 0.52 (0.46 to 0.57) | 0.46 (0.41 to 0.51) | 0.57 (0.52 to 0.63) | 0.56 (0.51 to 0.62) | 0.52 (0.46 to 0.57) | 0.46 (0.41 to 0.51) |
| T9 | 0.90 (0.82 to 0.95) | 0.81 (0.75 to 0.87) | 0.68 (0.62 to 0.73) | 0.54 (0.49 to 0.60) | 0.91 (0.84 to 0.97) | 0.86 (0.79 to 0.91) | 0.74 (0.68 to 0.80) | 0.59 (0.53 to 0.64) |
| P1 | 1.00 (1.00 to 1.00) | (1) | (1) | (1) | (1) | (1) | (1) | (1) |
| P2 | 0.60 (0.55 to 0.66) | (1) | (1) | (1) | (1) | (1) | (1) | (1) |
| P3 | 0.77 (0.73 to 0.81) | (1) | (1) | (1) | (1) | (1) | (1) | (1) |
| P4 | 1.00 (1.00 to 1.00) | (1) | (1) | (1) | (1) | (1) | (1) | (1) |
| P5 | 0.59 (0.54 to 0.64) | 0.54 (0.49 to 0.60) | 0.48 (0.43 to 0.53) | 0.43 (0.38 to 0.48) | 0.60 (0.55 to 0.65) | 0.57 (0.53 to 0.63) | 0.53 (0.48 to 0.58) | 0.46 (0.42 to 0.51) |
| P6 | 0.75 (0.70 to 0.79) | 0.67 (0.62 to 0.72) | 0.56 (0.51 to 0.61) | 0.47 (0.42 to 0.52) | 0.77 (0.72 to 0.81) | 0.72 (0.68 to 0.77) | 0.64 (0.59 to 0.68) | 0.53 (0.48 to 0.58) |
| P7 | 0.96 (0.94 to 0.98) | 0.84 (0.80 to 0.87) | 0.67 (0.62 to 0.71) | 0.52 (0.47 to 0.58) | 0.99 (0.98 to 1.00) | 0.92 (0.90 to 0.94) | 0.79 (0.74 to 0.82) | 0.61 (0.56 to 0.66) |
| P8 | 0.60 (0.55 to 0.66) | 0.59 (0.54 to 0.64) | 0.54 (0.49 to 0.59) | 0.47 (0.42 to 0.52) | 0.60 (0.55 to 0.66) | 0.59 (0.54 to 0.64) | 0.54 (0.49 to 0.59) | 0.47 (0.42 to 0.52) |
| P9 | 0.97 (0.96 to 0.98) | 0.88 (0.85 to 0.90) | 0.72 (0.67 to 0.76) | 0.56 (0.51 to 0.61) | 0.99 (0.98 to 1.00) | 0.93 (0.91 to 0.95) | 0.80 (0.75 to 0.83) | 0.62 (0.57 to 0.67) |
| (1) These strategies do not include MPMRI and therefore the results do not vary by MPMRI definition and cut-off | | | | | | | | |

Supplementary Table 15 – Proportion of CS cancers detected by strategy, mean (95% confidence intervals), TRUSB definition 2, MPMRI definition 1 and 2, MPMRI cut-off 2-5

| Strategy | 212 | 213 | 214 | 215 | 222 | 223 | 224 | 225 |
| --- | --- | --- | --- | --- | --- | --- | --- | --- |
| M1 | 0.71 (0.62 to 0.81) | 0.57 (0.50 to 0.66) | 0.38 (0.32 to 0.45) | 0.22 (0.17 to 0.27) | 0.74 (0.66 to 0.84) | 0.66 (0.58 to 0.76) | 0.51 (0.44 to 0.59) | 0.32 (0.27 to 0.38) |
| M2 | 0.75 (0.66 to 0.85) | 0.70 (0.61 to 0.79) | 0.54 (0.47 to 0.63) | 0.34 (0.28 to 0.40) | 0.75 (0.66 to 0.85) | 0.70 (0.61 to 0.79) | 0.54 (0.47 to 0.63) | 0.34 (0.28 to 0.40) |
| M3 | 0.79 (0.73 to 0.86) | 0.64 (0.58 to 0.71) | 0.42 (0.37 to 0.48) | 0.24 (0.20 to 0.29) | 0.83 (0.77 to 0.89) | 0.74 (0.68 to 0.81) | 0.57 (0.51 to 0.64) | 0.35 (0.30 to 0.41) |
| M4 | 0.84 (0.77 to 0.90) | 0.76 (0.70 to 0.83) | 0.59 (0.52 to 0.66) | 0.36 (0.31 to 0.42) | 0.84 (0.78 to 0.90) | 0.78 (0.71 to 0.84) | 0.60 (0.54 to 0.67) | 0.37 (0.32 to 0.43) |
| M5 | 0.82 (0.77 to 0.88) | 0.66 (0.60 to 0.73) | 0.44 (0.38 to 0.49) | 0.25 (0.20 to 0.29) | 0.86 (0.81 to 0.92) | 0.77 (0.72 to 0.83) | 0.59 (0.53 to 0.65) | 0.37 (0.32 to 0.42) |
| M6 | 0.87 (0.82 to 0.93) | 0.81 (0.76 to 0.86) | 0.63 (0.57 to 0.69) | 0.39 (0.33 to 0.45) | 0.87 (0.82 to 0.93) | 0.81 (0.76 to 0.86) | 0.63 (0.57 to 0.69) | 0.39 (0.33 to 0.45) |
| M7 | 0.91 (0.87 to 0.95) | 0.73 (0.68 to 0.78) | 0.48 (0.43 to 0.54) | 0.27 (0.22 to 0.32) | 0.95 (0.92 to 0.98) | 0.85 (0.81 to 0.89) | 0.65 (0.60 to 0.71) | 0.40 (0.35 to 0.46) |
| N1 | (2) | (2) | (2) | (2) | (2) | (2) | (2) | (2) |
| N2 | (2) | (2) | (2) | (2) | (2) | (2) | (2) | (2) |
| N3 | 0.81 (0.75 to 0.87) | 0.65 (0.59 to 0.71) | 0.43 (0.37 to 0.49) | 0.24 (0.20 to 0.29) | 0.85 (0.79 to 0.90) | 0.76 (0.70 to 0.81) | 0.58 (0.52 to 0.64) | 0.36 (0.31 to 0.42) |
| N4 | 0.85 (0.79 to 0.91) | 0.77 (0.71 to 0.84) | 0.59 (0.52 to 0.66) | 0.36 (0.31 to 0.42) | 0.85 (0.80 to 0.91) | 0.79 (0.73 to 0.85) | 0.61 (0.55 to 0.68) | 0.38 (0.32 to 0.44) |
| N5 | 0.84 (0.79 to 0.89) | 0.68 (0.62 to 0.74) | 0.44 (0.39 to 0.50) | 0.25 (0.20 to 0.30) | 0.88 (0.84 to 0.93) | 0.79 (0.74 to 0.84) | 0.61 (0.55 to 0.66) | 0.37 (0.32 to 0.43) |
| N6 | 0.89 (0.84 to 0.94) | 0.83 (0.78 to 0.87) | 0.64 (0.58 to 0.70) | 0.39 (0.34 to 0.45) | 0.89 (0.84 to 0.94) | 0.83 (0.78 to 0.87) | 0.64 (0.58 to 0.70) | 0.39 (0.34 to 0.45) |
| N7 | 0.94 (0.91 to 0.96) | 0.75 (0.70 to 0.80) | 0.49 (0.44 to 0.55) | 0.28 (0.23 to 0.33) | 0.98 (0.97 to 1.00) | 0.88 (0.84 to 0.91) | 0.67 (0.62 to 0.73) | 0.41 (0.36 to 0.47) |
| T1 | 0.61 (0.56 to 0.66) | (1) | (1) | (1) | (1) | (1) | (1) | (1) |
| T2 | 0.69 (0.64 to 0.74) | (1) | (1) | (1) | (1) | (1) | (1) | (1) |
| T3 | 0.65 (0.60 to 0.70) | (1) | (1) | (1) | (1) | (1) | (1) | (1) |
| T4 | 0.73 (0.69 to 0.78) | (1) | (1) | (1) | (1) | (1) | (1) | (1) |
| T5 | 0.80 (0.75 to 0.84) | 0.76 (0.71 to 0.81) | 0.71 (0.66 to 0.75) | 0.66 (0.61 to 0.71) | 0.81 (0.76 to 0.85) | 0.79 (0.74 to 0.83) | 0.74 (0.70 to 0.79) | 0.69 (0.64 to 0.74) |
| T6 | 0.74 (0.70 to 0.79) | 0.72 (0.67 to 0.76) | 0.68 (0.63 to 0.72) | 0.65 (0.60 to 0.69) | 0.75 (0.71 to 0.79) | 0.74 (0.69 to 0.78) | 0.71 (0.66 to 0.75) | 0.67 (0.62 to 0.71) |
| T7 | 0.93 (0.88 to 0.96) | 0.86 (0.82 to 0.90) | 0.77 (0.72 to 0.81) | 0.69 (0.65 to 0.74) | 0.95 (0.90 to 0.98) | 0.91 (0.86 to 0.94) | 0.83 (0.79 to 0.87) | 0.74 (0.69 to 0.78) |
| T8 | 0.81 (0.76 to 0.86) | 0.79 (0.75 to 0.84) | 0.75 (0.70 to 0.80) | 0.69 (0.64 to 0.74) | 0.81 (0.76 to 0.86) | 0.79 (0.75 to 0.84) | 0.75 (0.70 to 0.80) | 0.69 (0.64 to 0.74) |
| T9 | 0.94 (0.89 to 0.97) | 0.90 (0.85 to 0.93) | 0.82 (0.77 to 0.86) | 0.73 (0.68 to 0.77) | 0.95 (0.90 to 0.98) | 0.92 (0.87 to 0.95) | 0.84 (0.80 to 0.88) | 0.75 (0.70 to 0.79) |
| P1 | (2) | (1) | (1) | (1) | (1) | (1) | (1) | (1) |
| P2 | 0.84 (0.80 to 0.88) | (1) | (1) | (1) | (1) | (1) | (1) | (1) |
| P3 | 0.77 (0.73 to 0.81) | (1) | (1) | (1) | (1) | (1) | (1) | (1) |
| P4 | 1.00 (1.00 to 1.00) | (1) | (1) | (1) | (1) | (1) | (1) | (1) |
| P5 | 0.83 (0.78 to 0.87) | 0.78 (0.74 to 0.82) | 0.72 (0.67 to 0.76) | 0.67 (0.62 to 0.71) | 0.84 (0.79 to 0.88) | 0.81 (0.77 to 0.85) | 0.76 (0.72 to 0.80) | 0.70 (0.65 to 0.75) |
| P6 | 0.76 (0.72 to 0.80) | 0.73 (0.69 to 0.77) | 0.69 (0.64 to 0.73) | 0.65 (0.60 to 0.70) | 0.77 (0.73 to 0.81) | 0.75 (0.71 to 0.79) | 0.72 (0.68 to 0.76) | 0.67 (0.63 to 0.72) |
| P7 | 0.97 (0.96 to 0.99) | 0.90 (0.87 to 0.92) | 0.79 (0.75 to 0.83) | 0.70 (0.65 to 0.75) | 0.99 (0.99 to 1.00) | 0.95 (0.93 to 0.97) | 0.87 (0.83 to 0.90) | 0.76 (0.72 to 0.80) |
| P8 | 0.84 (0.80 to 0.88) | 0.82 (0.78 to 0.86) | 0.77 (0.73 to 0.81) | 0.71 (0.66 to 0.75) | 0.84 (0.80 to 0.88) | 0.82 (0.78 to 0.86) | 0.77 (0.73 to 0.81) | 0.71 (0.66 to 0.75) |
| P9 | 0.99 (0.98 to 0.99) | 0.94 (0.92 to 0.96) | 0.85 (0.81 to 0.88) | 0.74 (0.70 to 0.78) | 1.00 (0.99 to 1.00) | 0.96 (0.95 to 0.97) | 0.88 (0.85 to 0.90) | 0.77 (0.72 to 0.81) |
| (1) These strategies do not include MPMRI and therefore the results do not vary by MPMRI definition and cut-off.   (2) These strategies do not include TRUSB and therefore the results do not vary by TRUSB definition. | | | | | | | | |

Supplementary Table 16 – Costs of testing in pound by strategy, average (95% confidence intervals), TRUSB definition 1, MPMRI definition 1 and 2, MPMRI cut-off 2-5

| Strategy | 112 | 113 | 114 | 115 | 122 | 123 | 124 | 125 |
| --- | --- | --- | --- | --- | --- | --- | --- | --- |
| M1 | £544 (£531 to £556) | £409 (£392 to £427) | £310 (£294 to £325) | £244 (£232 to £257) | £581 (£573 to £588) | £482 (£467 to £499) | £366 (£349 to £383) | £280 (£265 to £295) |
| M2 | £596 (£592 to £600) | £522 (£509 to £536) | £381 (£364 to £399) | £287 (£272 to £301) | £596 (£592 to £600) | £522 (£509 to £536) | £381 (£364 to £399) | £287 (£272 to £301) |
| M3 | £631 (£611 to £654) | £474 (£451 to £499) | £349 (£327 to £371) | £264 (£248 to £280) | £674 (£656 to £693) | £562 (£539 to £587) | £423 (£398 to £447) | £311 (£291 to £332) |
| M4 | £684 (£667 to £701) | £587 (£568 to £607) | £421 (£399 to £442) | £306 (£287 to £324) | £689 (£672 to £707) | £602 (£581 to £624) | £438 (£415 to £461) | £318 (£298 to £338) |
| M5 | £716 (£692 to £739) | £488 (£464 to £514) | £342 (£322 to £363) | £257 (£242 to £273) | £780 (£759 to £800) | £608 (£583 to £635) | £418 (£397 to £442) | £301 (£284 to £320) |
| M6 | £807 (£790 to £826) | £678 (£653 to £702) | £441 (£418 to £465) | £310 (£292 to £328) | £807 (£790 to £826) | £678 (£653 to £702) | £441 (£418 to £465) | £310 (£292 to £328) |
| M7 | £804 (£776 to £831) | £553 (£524 to £583) | £382 (£354 to £408) | £277 (£258 to £296) | £873 (£850 to £896) | £687 (£657 to £719) | £475 (£447 to £504) | £333 (£310 to £357) |
| N1 | £1399 (£1357 to £1438) | £946 (£890 to £1004) | £613 (£561 to £664) | £392 (£349 to £435) | £1524 (£1500 to £1547) | £1193 (£1142 to £1245) | £801 (£745 to £857) | £512 (£464 to £560) |
| N2 | £1575 (£1565 to £1585) | £1328 (£1284 to £1371) | £853 (£797 to £912) | £534 (£485 to £581) | £1575 (£1565 to £1585) | £1328 (£1284 to £1371) | £853 (£797 to £912) | £534 (£485 to £581) |
| N3 | £839 (£784 to £897) | £628 (£581 to £677) | £443 (£404 to £483) | £309 (£284 to £339) | £895 (£836 to £953) | £749 (£696 to £805) | £557 (£508 to £605) | £386 (£350 to £422) |
| N4 | £891 (£838 to £946) | £741 (£698 to £788) | £514 (£476 to £554) | £352 (£323 to £380) | £911 (£853 to £967) | £789 (£737 to £844) | £572 (£525 to £621) | £392 (£356 to £428) |
| N5 | £1124 (£1065 to £1186) | £676 (£625 to £728) | £419 (£386 to £457) | £288 (£266 to £313) | £1252 (£1192 to £1311) | £906 (£848 to £967) | £542 (£500 to £588) | £351 (£324 to £381) |
| N6 | £1308 (£1249 to £1366) | £1046 (£987 to £1107) | £582 (£536 to £629) | £364 (£336 to £393) | £1308 (£1249 to £1366) | £1046 (£987 to £1107) | £582 (£536 to £629) | £364 (£336 to £393) |
| N7 | £1420 (£1348 to £1487) | £894 (£831 to £961) | £552 (£497 to £609) | £353 (£318 to £392) | £1566 (£1495 to £1629) | £1173 (£1098 to £1245) | £733 (£672 to £795) | £457 (£413 to £505) |
| T1 | £415 (£412 to £420) | (1) | (1) | (1) | (1) | (1) | (1) | (1) |
| T2 | £627 (£610 to £644) | (1) | (1) | (1) | (1) | (1) | (1) | (1) |
| T3 | £531 (£516 to £547) | (1) | (1) | (1) | (1) | (1) | (1) | (1) |
| T4 | £742 (£727 to £757) | (1) | (1) | (1) | (1) | (1) | (1) | (1) |
| T5 | £681 (£658 to £705) | £591 (£573 to £608) | £544 (£531 to £556) | £523 (£513 to £533) | £707 (£683 to £730) | £636 (£616 to £657) | £564 (£550 to £578) | £532 (£522 to £543) |
| T6 | £571 (£550 to £592) | £541 (£524 to £558) | £511 (£497 to £525) | £488 (£477 to £500) | £578 (£557 to £599) | £559 (£541 to £579) | £531 (£515 to £547) | £501 (£488 to £515) |
| T7 | £837 (£816 to £858) | £717 (£700 to £736) | £639 (£625 to £655) | £595 (£585 to £607) | £870 (£849 to £890) | £780 (£760 to £801) | £680 (£663 to £697) | £618 (£606 to £633) |
| T8 | £719 (£695 to £743) | £665 (£642 to £687) | £571 (£557 to £586) | £534 (£523 to £545) | £719 (£695 to £743) | £665 (£642 to £687) | £571 (£557 to £586) | £534 (£523 to £545) |
| T9 | £875 (£855 to £894) | £791 (£772 to £810) | £667 (£652 to £683) | £607 (£596 to £619) | £882 (£861 to £901) | £809 (£790 to £829) | £687 (£671 to £705) | £620 (£608 to £635) |
| P1 | £1398 (£1392 to £1405) | (1) | (1) | (1) | (1) | (1) | (1) | (1) |
| P2 | £1127 (£1070 to £1179) | (1) | (1) | (1) | (1) | (1) | (1) | (1) |
| P3 | £805 (£754 to £854) | (1) | (1) | (1) | (1) | (1) | (1) | (1) |
| P4 | £1517 (£1472 to £1561) | (1) | (1) | (1) | (1) | (1) | (1) | (1) |
| P5 | £1091 (£1031 to £1150) | £787 (£741 to £833) | £628 (£601 to £655) | £558 (£542 to £577) | £1179 (£1117 to £1240) | £940 (£890 to £995) | £698 (£664 to £733) | £590 (£569 to £614) |
| P6 | £819 (£767 to £871) | £720 (£677 to £762) | £618 (£585 to £653) | £539 (£518 to £563) | £844 (£789 to £899) | £781 (£732 to £829) | £686 (£647 to £727) | £585 (£556 to £614) |
| P7 | £1496 (£1440 to £1550) | £1092 (£1044 to £1143) | £831 (£789 to £872) | £682 (£655 to £713) | £1608 (£1554 to £1658) | £1305 (£1248 to £1363) | £968 (£922 to £1017) | £760 (£726 to £797) |
| P8 | £1219 (£1154 to £1278) | £1037 (£980 to £1093) | £722 (£686 to £759) | £597 (£575 to £622) | £1219 (£1154 to £1278) | £1037 (£980 to £1093) | £722 (£686 to £759) | £597 (£575 to £622) |
| P9 | £1638 (£1589 to £1686) | £1373 (£1319 to £1424) | £936 (£893 to £982) | £722 (£694 to £754) | £1654 (£1604 to £1702) | £1414 (£1361 to £1466) | £996 (£949 to £1045) | £767 (£733 to £803) |
| (1) These strategies do not include MPMRI and therefore the results do not vary by MPMRI definition and cut-off | | | | | | | | |

Supplementary Table 17 – Costs in pound of testing by strategy, mean (95% confidence intervals), TRUGB definition 2, MPMRI definition 1 and 2, MPMRI cut-off 2-5

| Strategy | 212 | 213 | 214 | 215 | 222 | 223 | 224 | 225 |
| --- | --- | --- | --- | --- | --- | --- | --- | --- |
| M1 | £544 (£531 to £556) | £409 (£392 to £427) | £310 (£294 to £325) | £244 (£232 to £257) | £581 (£573 to £588) | £482 (£467 to £499) | £366 (£349 to £383) | £280 (£265 to £295) |
| M2 | £596 (£592 to £600) | £522 (£509 to £536) | £381 (£364 to £399) | £287 (£272 to £301) | £596 (£592 to £600) | £522 (£509 to £536) | £381 (£364 to £399) | £287 (£272 to £301) |
| M3 | £577 (£560 to £595) | £431 (£412 to £451) | £323 (£305 to £341) | £250 (£237 to £264) | £617 (£603 to £633) | £511 (£492 to £532) | £385 (£366 to £404) | £290 (£273 to £306) |
| M4 | £630 (£618 to £643) | £545 (£528 to £561) | £394 (£377 to £413) | £292 (£276 to £308) | £632 (£620 to £646) | £551 (£534 to £570) | £400 (£382 to £420) | £296 (£280 to £312) |
| M5 | £707 (£682 to £731) | £481 (£456 to £507) | £338 (£317 to £358) | £255 (£240 to £271) | £770 (£746 to £791) | £599 (£572 to £625) | £412 (£390 to £435) | £298 (£279 to £316) |
| M6 | £797 (£777 to £818) | £669 (£642 to £694) | £434 (£411 to £458) | £306 (£287 to £323) | £797 (£777 to £818) | £669 (£642 to £694) | £434 (£411 to £458) | £306 (£287 to £323) |
| M7 | £740 (£710 to £769) | £503 (£475 to £532) | £350 (£328 to £374) | £261 (£244 to £278) | £807 (£777 to £833) | £628 (£597 to £660) | £430 (£404 to £458) | £307 (£287 to £328) |
| N1 | (2) | (2) | (2) | (2) | (2) | (2) | (2) | (2) |
| N2 | (2) | (2) | (2) | (2) | (2) | (2) | (2) | (2) |
| N3 | £657 (£620 to £703) | £484 (£450 to £522) | £353 (£328 to £380) | £264 (£246 to £283) | £704 (£663 to £750) | £580 (£543 to £625) | £429 (£399 to £463) | £312 (£289 to £336) |
| N4 | £709 (£672 to £753) | £598 (£565 to £633) | £424 (£400 to £452) | £306 (£286 to £326) | £719 (£678 to £766) | £620 (£583 to £663) | £445 (£414 to £478) | £319 (£296 to £342) |
| N5 | £1092 (£1023 to £1157) | £651 (£597 to £706) | £403 (£369 to £442) | £280 (£258 to £306) | £1218 (£1147 to £1284) | £876 (£812 to £941) | £520 (£474 to £568) | £339 (£310 to £370) |
| N6 | £1275 (£1210 to £1339) | £1015 (£950 to £1080) | £558 (£509 to £608) | £350 (£320 to £383) | £1275 (£1210 to £1339) | £1015 (£950 to £1080) | £558 (£509 to £608) | £350 (£320 to £383) |
| N7 | £1206 (£1118 to £1286) | £726 (£653 to £796) | £446 (£398 to £500) | £300 (£272 to £333) | £1341 (£1251 to £1423) | £973 (£891 to £1051) | £583 (£522 to £645) | £371 (£333 to £411) |
| T1 | £415 (£412 to £420) | (1) | (1) | (1) | (1) | (1) | (1) | (1) |
| T2 | £627 (£610 to £644) | (1) | (1) | (1) | (1) | (1) | (1) | (1) |
| T3 | £476 (£463 to £489) | (1) | (1) | (1) | (1) | (1) | (1) | (1) |
| T4 | £687 (£671 to £705) | (1) | (1) | (1) | (1) | (1) | (1) | (1) |
| T5 | £681 (£658 to £705) | £591 (£573 to £608) | £544 (£531 to £556) | £523 (£513 to £533) | £707 (£683 to £730) | £636 (£616 to £657) | £564 (£550 to £578) | £532 (£522 to £543) |
| T6 | £496 (£480 to £511) | £477 (£464 to £490) | £462 (£452 to £472) | £451 (£443 to £459) | £500 (£484 to £517) | £488 (£473 to £503) | £471 (£460 to £484) | £457 (£448 to £466) |
| T7 | £762 (£738 to £785) | £653 (£635 to £671) | £590 (£577 to £604) | £559 (£549 to £569) | £792 (£769 to £816) | £709 (£688 to £730) | £620 (£605 to £637) | £574 (£562 to £586) |
| T8 | £719 (£695 to £743) | £665 (£642 to £687) | £571 (£557 to £586) | £534 (£523 to £545) | £719 (£695 to £743) | £665 (£642 to £687) | £571 (£557 to £586) | £534 (£523 to £545) |
| T9 | £799 (£776 to £822) | £727 (£705 to £747) | £618 (£603 to £634) | £570 (£559 to £582) | £804 (£781 to £828) | £737 (£715 to £759) | £627 (£611 to £644) | £576 (£564 to £588) |
| P1 | (1)(2) | (1) | (1) | (1) | (1) | (1) | (1) | (1) |
| P2 | £1127 (£1070 to £1179) | (1) | (1) | (1) | (1) | (1) | (1) | (1) |
| P3 | £620 (£580 to £660) | (1) | (1) | (1) | (1) | (1) | (1) | (1) |
| P4 | £1332 (£1278 to £1385) | (1) | (1) | (1) | (1) | (1) | (1) | (1) |
| P5 | £1091 (£1031 to £1150) | £787 (£741 to £833) | £628 (£601 to £655) | £558 (£542 to £577) | £1179 (£1117 to £1240) | £940 (£890 to £995) | £698 (£664 to £733) | £590 (£569 to £614) |
| P6 | £623 (£582 to £663) | £560 (£530 to £591) | £509 (£489 to £531) | £472 (£459 to £486) | £639 (£595 to £683) | £596 (£560 to £633) | £541 (£515 to £569) | £491 (£474 to £509) |
| P7 | £1299 (£1238 to £1358) | £932 (£884 to £979) | £721 (£689 to £757) | £615 (£595 to £637) | £1402 (£1341 to £1462) | £1120 (£1066 to £1181) | £823 (£782 to £867) | £666 (£639 to £695) |
| P8 | £1219 (£1154 to £1278) | £1037 (£980 to £1093) | £722 (£686 to £759) | £597 (£575 to £622) | £1219 (£1154 to £1278) | £1037 (£980 to £1093) | £722 (£686 to £759) | £597 (£575 to £622) |
| P9 | £1442 (£1383 to £1500) | £1213 (£1157 to £1269) | £826 (£786 to £869) | £655 (£631 to £682) | £1448 (£1387 to £1507) | £1229 (£1171 to £1288) | £851 (£808 to £896) | £673 (£645 to £702) |
| (1) These strategies do not include MPMRI and therefore the results do not vary by MPMRI definition and cut-off.   (2) These strategies do not include TRUSB and therefore the results do not vary by TRUGB definition. | | | | | | | | |

Supplementary Table 18 – Costs in euro of testing by strategy, average (95% confidence intervals), TRUSB definition 1, MPMRI definition 1 and 2, MPMRI cut-off 2-5

| Strategy | 112 | 113 | 114 | 115 | 122 | 123 | 124 | 125 |
| --- | --- | --- | --- | --- | --- | --- | --- | --- |
| M1 | 641 (627 to 656) | 483 (463 to 504) | 366 (347 to 384) | 288 (274 to 303) | 685 (676 to 694) | 569 (551 to 588) | 432 (412 to 452) | 331 (313 to 348) |
| M2 | 703 (698 to 709) | 617 (601 to 632) | 450 (430 to 471) | 338 (321 to 355) | 703 (698 to 709) | 617 (601 to 632) | 450 (430 to 471) | 338 (321 to 355) |
| M3 | 745 (721 to 772) | 559 (532 to 589) | 412 (386 to 438) | 311 (292 to 331) | 795 (774 to 818) | 663 (637 to 693) | 499 (470 to 527) | 368 (344 to 392) |
| M4 | 807 (787 to 827) | 693 (670 to 717) | 497 (471 to 522) | 361 (339 to 382) | 813 (793 to 834) | 710 (685 to 737) | 517 (489 to 545) | 375 (351 to 398) |
| M5 | 845 (817 to 872) | 576 (547 to 606) | 404 (380 to 429) | 304 (286 to 323) | 920 (896 to 944) | 718 (687 to 749) | 494 (468 to 522) | 356 (335 to 378) |
| M6 | 953 (932 to 974) | 800 (771 to 829) | 520 (493 to 549) | 365 (344 to 386) | 953 (932 to 974) | 800 (771 to 829) | 520 (493 to 549) | 365 (344 to 386) |
| M7 | 949 (916 to 980) | 653 (619 to 688) | 450 (418 to 481) | 326 (304 to 350) | 1031 (1003 to 1058) | 811 (775 to 848) | 560 (527 to 595) | 392 (366 to 421) |
| N1 | 1651 (1601 to 1697) | 1116 (1050 to 1185) | 723 (662 to 784) | 463 (412 to 513) | 1799 (1770 to 1825) | 1408 (1347 to 1470) | 946 (879 to 1011) | 605 (547 to 661) |
| N2 | 1859 (1847 to 1870) | 1567 (1515 to 1618) | 1007 (941 to 1076) | 631 (572 to 686) | 1859 (1847 to 1870) | 1567 (1515 to 1618) | 1007 (941 to 1076) | 631 (572 to 686) |
| N3 | 990 (925 to 1059) | 741 (685 to 799) | 523 (477 to 570) | 365 (335 to 400) | 1057 (987 to 1124) | 884 (822 to 950) | 657 (599 to 714) | 455 (413 to 498) |
| N4 | 1052 (989 to 1116) | 875 (823 to 930) | 607 (562 to 654) | 415 (382 to 449) | 1074 (1006 to 1141) | 931 (870 to 996) | 675 (619 to 733) | 463 (420 to 505) |
| N5 | 1327 (1256 to 1399) | 797 (738 to 859) | 494 (455 to 539) | 340 (314 to 369) | 1477 (1406 to 1546) | 1069 (1001 to 1141) | 640 (590 to 694) | 415 (382 to 450) |
| N6 | 1544 (1474 to 1612) | 1235 (1165 to 1306) | 686 (632 to 742) | 430 (396 to 464) | 1544 (1474 to 1612) | 1235 (1165 to 1306) | 686 (632 to 742) | 430 (396 to 464) |
| N7 | 1675 (1591 to 1754) | 1055 (981 to 1134) | 651 (587 to 718) | 417 (375 to 462) | 1848 (1764 to 1923) | 1384 (1295 to 1469) | 865 (793 to 938) | 539 (488 to 596) |
| T1 | 490 (486 to 495) | (1) | (1) | (1) | (1) | (1) | (1) | (1) |
| T2 | 740 (720 to 760) | (1) | (1) | (1) | (1) | (1) | (1) | (1) |
| T3 | 627 (608 to 645) | (1) | (1) | (1) | (1) | (1) | (1) | (1) |
| T4 | 876 (858 to 893) | (1) | (1) | (1) | (1) | (1) | (1) | (1) |
| T5 | 804 (777 to 831) | 697 (676 to 718) | 641 (627 to 656) | 617 (606 to 629) | 835 (805 to 862) | 751 (727 to 775) | 666 (649 to 683) | 628 (616 to 641) |
| T6 | 674 (649 to 698) | 639 (618 to 659) | 603 (586 to 620) | 576 (563 to 590) | 682 (658 to 707) | 660 (638 to 683) | 627 (607 to 646) | 591 (576 to 607) |
| T7 | 988 (963 to 1013) | 846 (826 to 868) | 755 (737 to 773) | 703 (690 to 716) | 1027 (1002 to 1050) | 921 (897 to 946) | 803 (783 to 823) | 730 (715 to 747) |
| T8 | 848 (820 to 876) | 785 (758 to 810) | 674 (657 to 692) | 631 (618 to 644) | 848 (820 to 876) | 785 (758 to 810) | 674 (657 to 692) | 631 (618 to 644) |
| T9 | 1032 (1009 to 1055) | 934 (910 to 955) | 787 (769 to 806) | 716 (703 to 731) | 1041 (1016 to 1063) | 955 (932 to 978) | 811 (792 to 831) | 732 (718 to 749) |
| P1 | 1650 (1643 to 1658) | (1) | (1) | (1) | (1) | (1) | (1) | (1) |
| P2 | 1330 (1262 to 1392) | (1) | (1) | (1) | (1) | (1) | (1) | (1) |
| P3 | 950 (890 to 1007) | (1) | (1) | (1) | (1) | (1) | (1) | (1) |
| P4 | 1790 (1737 to 1842) | (1) | (1) | (1) | (1) | (1) | (1) | (1) |
| P5 | 1288 (1216 to 1357) | 929 (874 to 983) | 741 (709 to 773) | 659 (640 to 681) | 1391 (1318 to 1463) | 1109 (1050 to 1174) | 823 (783 to 865) | 697 (671 to 725) |
| P6 | 967 (905 to 1027) | 850 (798 to 900) | 729 (690 to 771) | 636 (611 to 665) | 996 (931 to 1060) | 921 (863 to 979) | 809 (763 to 857) | 690 (656 to 725) |
| P7 | 1765 (1700 to 1829) | 1289 (1232 to 1348) | 980 (932 to 1029) | 805 (773 to 841) | 1897 (1834 to 1956) | 1540 (1473 to 1609) | 1142 (1088 to 1200) | 896 (857 to 941) |
| P8 | 1438 (1362 to 1508) | 1224 (1157 to 1290) | 852 (810 to 895) | 705 (679 to 733) | 1438 (1362 to 1508) | 1224 (1157 to 1290) | 852 (810 to 895) | 705 (679 to 733) |
| P9 | 1933 (1875 to 1990) | 1620 (1556 to 1680) | 1104 (1054 to 1159) | 852 (819 to 889) | 1951 (1893 to 2009) | 1669 (1606 to 1730) | 1175 (1120 to 1233) | 905 (865 to 948) |
| (1) These strategies do not include MPMRI and therefore the results do not vary by MPMRI definition and cut-off | | | | | | | | |

Supplementary Table 19 – Costs in euro of testing by strategy, average (95% confidence intervals), TRUSB definition 1, MPMRI definition 1 and 2, MPMRI cut-off 2-5

| Strategy | 212 | 213 | 214 | 215 | 222 | 223 | 224 | 225 |
| --- | --- | --- | --- | --- | --- | --- | --- | --- |
| M1 | 641 (627 to 656) | 483 (463 to 504) | 366 (347 to 384) | 288 (274 to 303) | 685 (676 to 694) | 569 (551 to 588) | 432 (412 to 452) | 331 (313 to 348) |
| M2 | 703 (698 to 709) | 617 (601 to 632) | 450 (430 to 471) | 338 (321 to 355) | 703 (698 to 709) | 617 (601 to 632) | 450 (430 to 471) | 338 (321 to 355) |
| M3 | 681 (661 to 702) | 509 (486 to 533) | 381 (359 to 402) | 295 (279 to 311) | 728 (712 to 746) | 603 (581 to 627) | 454 (431 to 477) | 342 (322 to 361) |
| M4 | 743 (729 to 758) | 643 (623 to 662) | 465 (445 to 487) | 345 (326 to 363) | 746 (732 to 763) | 651 (630 to 672) | 472 (450 to 496) | 349 (330 to 368) |
| M5 | 834 (805 to 863) | 567 (538 to 599) | 398 (374 to 423) | 301 (283 to 320) | 909 (881 to 934) | 707 (675 to 738) | 486 (460 to 513) | 351 (329 to 373) |
| M6 | 941 (917 to 965) | 789 (758 to 819) | 512 (485 to 541) | 361 (339 to 382) | 941 (917 to 965) | 789 (758 to 819) | 512 (485 to 541) | 361 (339 to 382) |
| M7 | 874 (837 to 907) | 594 (561 to 628) | 413 (387 to 441) | 308 (288 to 328) | 952 (917 to 982) | 741 (704 to 778) | 508 (477 to 540) | 362 (339 to 387) |
| N1 | (2) | (2) | (2) | (2) | (2) | (2) | (2) | (2) |
| N2 | (2) | (2) | (2) | (2) | (2) | (2) | (2) | (2) |
| N3 | 775 (731 to 830) | 571 (531 to 616) | 416 (387 to 448) | 312 (291 to 334) | 831 (783 to 885) | 684 (641 to 737) | 507 (471 to 547) | 368 (342 to 397) |
| N4 | 837 (793 to 889) | 705 (667 to 746) | 501 (472 to 533) | 361 (338 to 385) | 849 (800 to 904) | 731 (688 to 782) | 525 (489 to 565) | 376 (350 to 404) |
| N5 | 1289 (1207 to 1365) | 768 (704 to 833) | 476 (435 to 522) | 330 (305 to 361) | 1437 (1353 to 1515) | 1034 (959 to 1110) | 613 (560 to 671) | 400 (366 to 436) |
| N6 | 1504 (1428 to 1580) | 1198 (1121 to 1275) | 658 (601 to 717) | 413 (378 to 452) | 1504 (1428 to 1580) | 1198 (1121 to 1275) | 658 (601 to 717) | 413 (378 to 452) |
| N7 | 1423 (1319 to 1517) | 856 (771 to 939) | 527 (470 to 590) | 354 (321 to 393) | 1583 (1476 to 1680) | 1149 (1051 to 1240) | 688 (616 to 761) | 437 (393 to 485) |
| T1 | 490 (486 to 495) | (1) | (1) | (1) | (1) | (1) | (1) | (1) |
| T2 | 740 (720 to 760) | (1) | (1) | (1) | (1) | (1) | (1) | (1) |
| T3 | 562 (547 to 577) | (1) | (1) | (1) | (1) | (1) | (1) | (1) |
| T4 | 811 (792 to 832) | (1) | (1) | (1) | (1) | (1) | (1) | (1) |
| T5 | 804 (777 to 831) | 697 (676 to 718) | 641 (627 to 656) | 617 (606 to 629) | 835 (805 to 862) | 751 (727 to 775) | 666 (649 to 683) | 628 (616 to 641) |
| T6 | 585 (566 to 603) | 563 (548 to 579) | 545 (533 to 557) | 532 (523 to 542) | 590 (571 to 611) | 575 (559 to 593) | 556 (542 to 571) | 539 (528 to 550) |
| T7 | 899 (871 to 926) | 770 (749 to 791) | 696 (681 to 712) | 659 (647 to 672) | 935 (907 to 963) | 836 (812 to 861) | 732 (714 to 752) | 677 (663 to 691) |
| T8 | 848 (820 to 876) | 785 (758 to 810) | 674 (657 to 692) | 631 (618 to 644) | 848 (820 to 876) | 785 (758 to 810) | 674 (657 to 692) | 631 (618 to 644) |
| T9 | 943 (915 to 970) | 857 (832 to 882) | 729 (712 to 748) | 673 (660 to 686) | 949 (921 to 977) | 870 (844 to 896) | 740 (721 to 760) | 679 (665 to 694) |
| P1 | (2) | (1) | (1) | (1) | (1) | (1) | (1) | (1) |
| P2 | 1330 (1262 to 1392) | (1) | (1) | (1) | (1) | (1) | (1) | (1) |
| P3 | 732 (685 to 778) | (1) | (1) | (1) | (1) | (1) | (1) | (1) |
| P4 | 1571 (1508 to 1634) | (1) | (1) | (1) | (1) | (1) | (1) | (1) |
| P5 | 1288 (1216 to 1357) | 929 (874 to 983) | 741 (709 to 773) | 659 (640 to 681) | 1391 (1318 to 1463) | 1109 (1050 to 1174) | 823 (783 to 865) | 697 (671 to 725) |
| P6 | 735 (687 to 783) | 660 (626 to 698) | 600 (577 to 626) | 557 (542 to 574) | 753 (702 to 806) | 703 (661 to 746) | 638 (608 to 671) | 579 (559 to 601) |
| P7 | 1533 (1461 to 1602) | 1099 (1043 to 1155) | 851 (813 to 893) | 726 (702 to 752) | 1655 (1582 to 1725) | 1322 (1257 to 1394) | 971 (923 to 1023) | 786 (755 to 820) |
| P8 | 1438 (1362 to 1508) | 1224 (1157 to 1290) | 852 (810 to 895) | 705 (679 to 733) | 1438 (1362 to 1508) | 1224 (1157 to 1290) | 852 (810 to 895) | 705 (679 to 733) |
| P9 | 1701 (1632 to 1770) | 1431 (1365 to 1497) | 975 (928 to 1026) | 773 (744 to 805) | 1708 (1636 to 1779) | 1451 (1382 to 1520) | 1004 (954 to 1057) | 794 (761 to 828) |
| (1) These strategies do not include MPMRI and therefore the results do not vary by MPMRI definition and cut-off.   (2) These strategies do not include TRUSB and therefore the results do not vary by TRUGB definition. | | | | | | | | |

Supplementary Table 20 – Diagnostic strategies in the efficiency frontier mean (95% confidence interval)

| Strategy | TRUSB definition | MPMRI definition | MPMRI cut-off | Detection  CS cancers | Cost  of testing in pound | Cost  of testing in euro |
| --- | --- | --- | --- | --- | --- | --- |
| M1: MPMRI for all men; TRUSB in men suspicious of CS cancer | 2 | 1 | 5 | 0.22 (0.17 to 0.27) | £244 (£232 to £257) | 288 (274 to 303) |
| M3: MPMRI for all men; TRUSB in men with suspicion on CS cancer; Men with NON-CS at 1st biopsy receive 2nd TRUSB | 2 | 1 | 5 | 0.24 (0.20 to 0.29) | £250 (£237 to £264) | 295 (279 to 311) |
|  | 2 | 2 | 5 | 0.35 (0.30 to 0.41) | £290 (£273 to £306) | 342 (322 to 361) |
| M4: MPMRI for all men; TRUS-guided in men with suspicion of any cancer. Men with suspicion of CS cancer at MPMRI and in whom NON-CS cancer was detected at the 1st biopsy receive 2nd TRUSB. | 2 | 2 | 5 | 0.37 (0.32 to 0.43) | £296 (£280 to £312) | 349 (330 to 368) |
| M7: MPMRI for all men; TRUSB in men with suspicion of CS cancer. Re-biopsy with TRUSB those in whom CS cancer was not detected | 2 | 2 | 5 | 0.40 (0.35 to 0.46) | £307 (£287 to £328) | 362 (339 to 387) |
| M3: MPMRI for all men; TRUSB in men with suspicion on CS cancer; Men with NON-CS at 1st biopsy receive 2nd TRUSB | 2 | 2 | 4 | 0.57 (0.51 to 0.64) | £385 (£366 to £404) | 454 (431 to 477) |
| M4: MPMRI for all men; TRUS-guided in men with suspicion of any cancer. Men with suspicion of CS cancer at MPMRI and in whom NON-CS cancer was detected at the 1st biopsy receive 2nd TRUSB. | 2 | 2 | 4 | 0.60 (0.54 to 0.67) | £400 (£382 to £420) | 472 (450 to 496) |
| M7: MPMRI for all men; TRUSB in men with suspicion of CS cancer.  Re-biopsy with TRUSB those in whom CS cancer was not detected | 2 | 2 | 4 | 0.65 (0.60 to 0.71) | £430 (£404 to £458) | 508 (477 to 540) |
| T6: TRUSB for all men; Men classified as NON-CS receive a MRI. Men with suspicion of CS cancer receive a 2nd TRUSB | 2 | 2 | 3 | 0.74 (0.69 to 0.78) | £488 (£473 to £503) | 575 (559 to 593) |
|  | 2 | 2 | 2 | 0.75 (0.71 to 0.79) | £500 (£484 to £517) | 590 (571 to 611) |
| M7: MPMRI for all men; TRUSB in men with suspicion of CS cancer. Re-biopsy with TRUSB those in whom CS cancer was not detected | 2 | 2 | 3 | 0.85 (0.81 to 0.89) | £628 (£597 to £660) | 741 (704 to 778) |
| T7: TRUSB for all men; Men classified as NC or NON-CS receive a MPMRI. Men with suspicion of CS cancer receive a 2nd TRUSB | 2 | 2 | 3 | 0.91 (0.86 to 0.94) | £709 (£688 to £730) | 836 (812 to 861) |
| M7: MPMRI for all men; TRUSB in men with suspicion of CS cancer  Re-biopsy with TRUSB those in whom CS cancer was not detected | 2 | 2 | 2 | 0.95 (0.92 to 0.98) | £807 (£777 to £833) | 952 (917 to 982) |
| P4: TRUSB in all men and TPM-biopsy in men in whom the biopsy did not detect CS cancer. | 2 | Not applicable | | 1.00 (1.00 to 1.00) | £1332 (£1278 to £1385) |  |

# Cost-effectiveness results

Supplementary Tables 18 and 19 show the lifetime health outcomes achieved by strategy, and Supplementary Tables 20 and 21 show the lifetime costs. Supplementary Table 22 shows the strategies forming the cost-effectiveness frontier at their expected values. Supplementary Figure 1 shows the cost-effectiveness acceptability frontier.

Supplementary Fig. 2 – Cost-effectiveness acceptability frontier


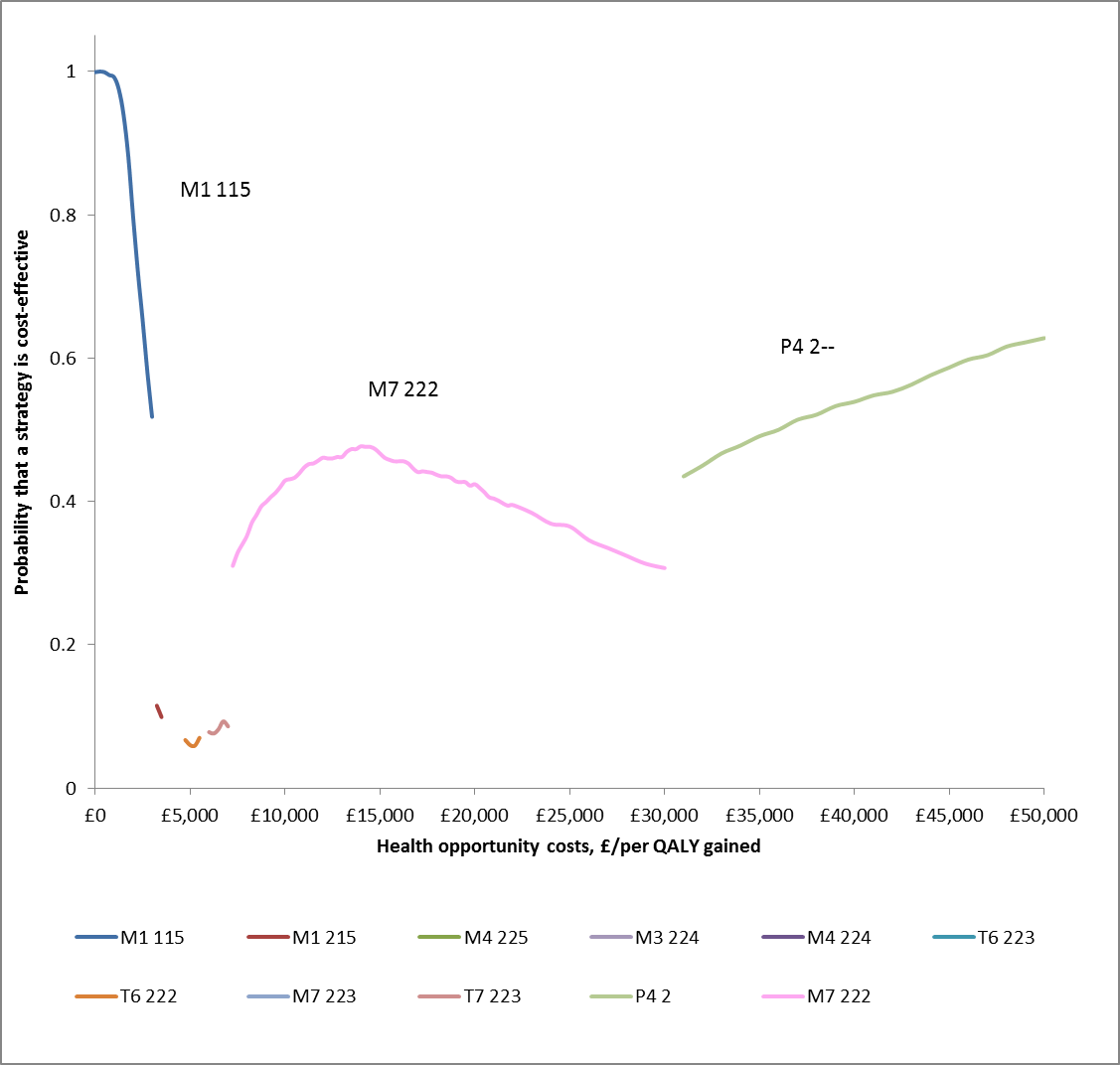


Supplementary Table 21 – Lifetime health outcomes by strategy, mean QALYs (95% confidence intervals), TRUS-guided definition 1, MPMRI definition 1 and 2, MPMRI cut-off 2-5

| Strategy | 112 | 113 | 114 | 115 | 122 | 123 | 124 | 125 |
| --- | --- | --- | --- | --- | --- | --- | --- | --- |
| M1 | 8.44 (8.14 to 8.76) | 8.40 (8.09 to 8.73) | 8.34 (8.01 to 8.70) | 8.29 (7.94 to 8.68) | 8.45 (8.15 to 8.78) | 8.43 (8.13 to 8.75) | 8.38 (8.07 to 8.72) | 8.32 (7.99 to 8.69) |
| M2 | 8.46 (8.16 to 8.78) | 8.44 (8.14 to 8.76) | 8.39 (8.08 to 8.73) | 8.33 (7.99 to 8.69) | 8.46 (8.16 to 8.78) | 8.44 (8.14 to 8.76) | 8.39 (8.08 to 8.73) | 8.33 (7.99 to 8.69) |
| M3 | 8.60 (8.30 to 8.90) | 8.52 (8.23 to 8.82) | 8.42 (8.12 to 8.74) | 8.33 (8.00 to 8.69) | 8.61 (8.32 to 8.91) | 8.57 (8.28 to 8.87) | 8.49 (8.20 to 8.80) | 8.38 (8.07 to 8.72) |
| M4 | 8.61 (8.32 to 8.91) | 8.56 (8.27 to 8.86) | 8.47 (8.18 to 8.78) | 8.37 (8.06 to 8.71) | 8.62 (8.32 to 8.92) | 8.58 (8.29 to 8.88) | 8.50 (8.21 to 8.81) | 8.39 (8.09 to 8.73) |
| M5 | 8.52 (8.24 to 8.82) | 8.46 (8.17 to 8.77) | 8.38 (8.06 to 8.72) | 8.31 (7.97 to 8.68) | 8.54 (8.26 to 8.83) | 8.50 (8.22 to 8.81) | 8.44 (8.14 to 8.75) | 8.36 (8.03 to 8.71) |
| M6 | 8.54 (8.26 to 8.84) | 8.52 (8.23 to 8.82) | 8.45 (8.16 to 8.77) | 8.36 (8.05 to 8.71) | 8.54 (8.26 to 8.84) | 8.52 (8.23 to 8.82) | 8.45 (8.16 to 8.77) | 8.36 (8.05 to 8.71) |
| M7 | 8.68 (8.38 to 8.99) | 8.58 (8.30 to 8.89) | 8.46 (8.17 to 8.77) | 8.35 (8.03 to 8.70) | 8.70 (8.39 to 9.02) | 8.65 (8.35 to 8.95) | 8.55 (8.26 to 8.84) | 8.42 (8.12 to 8.74) |
| N1 | 8.70 (8.39 to 9.02) | 8.61 (8.31 to 8.90) | 8.47 (8.18 to 8.78) | 8.36 (8.04 to 8.70) | 8.73 (8.40 to 9.05) | 8.67 (8.37 to 8.98) | 8.57 (8.28 to 8.86) | 8.43 (8.13 to 8.75) |
| N2 | 8.73 (8.40 to 9.06) | 8.69 (8.38 to 9.01) | 8.59 (8.30 to 8.89) | 8.44 (8.14 to 8.76) | 8.73 (8.40 to 9.06) | 8.69 (8.38 to 9.01) | 8.59 (8.30 to 8.89) | 8.44 (8.14 to 8.76) |
| N3 | 8.62 (8.32 to 8.91) | 8.54 (8.25 to 8.84) | 8.43 (8.13 to 8.75) | 8.34 (8.00 to 8.70) | 8.64 (8.34 to 8.94) | 8.59 (8.30 to 8.88) | 8.50 (8.22 to 8.81) | 8.39 (8.09 to 8.73) |
| N4 | 8.63 (8.34 to 8.93) | 8.58 (8.28 to 8.87) | 8.48 (8.19 to 8.79) | 8.37 (8.06 to 8.71) | 8.64 (8.34 to 8.94) | 8.60 (8.31 to 8.89) | 8.51 (8.22 to 8.82) | 8.40 (8.09 to 8.73) |
| N5 | 8.53 (8.25 to 8.83) | 8.47 (8.18 to 8.78) | 8.39 (8.07 to 8.72) | 8.32 (7.97 to 8.69) | 8.55 (8.26 to 8.84) | 8.51 (8.23 to 8.81) | 8.45 (8.15 to 8.76) | 8.36 (8.04 to 8.71) |
| N6 | 8.55 (8.27 to 8.84) | 8.53 (8.24 to 8.82) | 8.46 (8.17 to 8.77) | 8.37 (8.05 to 8.71) | 8.55 (8.27 to 8.84) | 8.53 (8.24 to 8.82) | 8.46 (8.17 to 8.77) | 8.37 (8.05 to 8.71) |
| N7 | 8.70 (8.39 to 9.02) | 8.61 (8.31 to 8.90) | 8.47 (8.19 to 8.78) | 8.36 (8.04 to 8.70) | 8.73 (8.40 to 9.06) | 8.67 (8.37 to 8.98) | 8.57 (8.28 to 8.86) | 8.43 (8.13 to 8.75) |
| T1 | 8.41 (8.11 to 8.74) | (1) | (1) | (1) | (1) | (1) | (1) | (1) |
| T2 | 8.44 (8.14 to 8.75) | (1) | (1) | (1) | (1) | (1) | (1) | (1) |
| T3 | 8.47 (8.16 to 8.78) | (1) | (1) | (1) | (1) | (1) | (1) | (1) |
| T4 | 8.49 (8.19 to 8.80) | (1) | (1) | (1) | (1) | (1) | (1) | (1) |
| T5 | 8.51 (8.22 to 8.81) | 8.49 (8.20 to 8.80) | 8.46 (8.17 to 8.78) | 8.44 (8.14 to 8.75) | 8.52 (8.23 to 8.82) | 8.50 (8.22 to 8.81) | 8.48 (8.19 to 8.79) | 8.45 (8.15 to 8.77) |
| T6 | 8.58 (8.29 to 8.88) | 8.55 (8.26 to 8.84) | 8.50 (8.21 to 8.81) | 8.46 (8.16 to 8.77) | 8.59 (8.30 to 8.89) | 8.57 (8.28 to 8.87) | 8.53 (8.25 to 8.83) | 8.48 (8.19 to 8.80) |
| T7 | 8.68 (8.38 to 9.00) | 8.62 (8.33 to 8.93) | 8.55 (8.26 to 8.84) | 8.48 (8.19 to 8.79) | 8.69 (8.38 to 9.02) | 8.66 (8.36 to 8.97) | 8.60 (8.31 to 8.91) | 8.52 (8.24 to 8.82) |
| T8 | 8.52 (8.23 to 8.82) | 8.51 (8.22 to 8.81) | 8.49 (8.19 to 8.80) | 8.45 (8.16 to 8.77) | 8.52 (8.23 to 8.82) | 8.51 (8.22 to 8.81) | 8.49 (8.19 to 8.80) | 8.45 (8.16 to 8.77) |
| T9 | 8.69 (8.38 to 9.01) | 8.64 (8.35 to 8.95) | 8.57 (8.29 to 8.87) | 8.50 (8.21 to 8.81) | 8.70 (8.39 to 9.02) | 8.67 (8.37 to 8.98) | 8.61 (8.31 to 8.91) | 8.52 (8.24 to 8.82) |
| P1 | 8.73 (8.40 to 9.06) | (1) | (1) | (1) | (1) | (1) | (1) | (1) |
| P2 | 8.53 (8.25 to 8.83) | (1) | (1) | (1) | (1) | (1) | (1) | (1) |
| P3 | 8.62 (8.33 to 8.92) | (1) | (1) | (1) | (1) | (1) | (1) | (1) |
| P4 | 8.74 (8.41 to 9.06) | (1) | (1) | (1) | (1) | (1) | (1) | (1) |
| P5 | 8.52 (8.24 to 8.82) | 8.50 (8.21 to 8.80) | 8.47 (8.17 to 8.78) | 8.44 (8.14 to 8.75) | 8.53 (8.25 to 8.83) | 8.51 (8.23 to 8.81) | 8.49 (8.20 to 8.80) | 8.46 (8.16 to 8.77) |
| P6 | 8.61 (8.31 to 8.90) | 8.57 (8.27 to 8.86) | 8.51 (8.23 to 8.81) | 8.46 (8.16 to 8.77) | 8.62 (8.33 to 8.91) | 8.59 (8.30 to 8.89) | 8.55 (8.26 to 8.84) | 8.49 (8.21 to 8.80) |
| P7 | 8.72 (8.40 to 9.04) | 8.65 (8.36 to 8.95) | 8.56 (8.29 to 8.86) | 8.49 (8.20 to 8.80) | 8.73 (8.40 to 9.06) | 8.70 (8.38 to 9.00) | 8.63 (8.33 to 8.93) | 8.54 (8.25 to 8.83) |
| P8 | 8.53 (8.25 to 8.83) | 8.52 (8.24 to 8.82) | 8.50 (8.21 to 8.80) | 8.46 (8.17 to 8.77) | 8.53 (8.25 to 8.83) | 8.52 (8.24 to 8.82) | 8.50 (8.21 to 8.80) | 8.46 (8.17 to 8.77) |
| P9 | 8.72 (8.40 to 9.05) | 8.67 (8.38 to 8.98) | 8.59 (8.30 to 8.89) | 8.51 (8.22 to 8.81) | 8.73 (8.40 to 9.06) | 8.70 (8.39 to 9.01) | 8.63 (8.34 to 8.94) | 8.54 (8.26 to 8.83) |
| (1) These strategies do not include MPMRI and therefore the results do not vary by MPMRI definition and cut-off | | | | | | | | |

Supplementary Table 22 – Lifetime health outcomes by strategy, mean QALYs (95% confidence intervals), TRUS-guided definition 2, MPMRI definition 1 and 2, MPMRI cut-off 2-5

| **Strategy** | **212** | **213** | **214** | **215** | **222** | **223** | **224** | **225** |
| --- | --- | --- | --- | --- | --- | --- | --- | --- |
| M1 | 8.59 (8.29 to 8.89) | 8.52 (8.22 to 8.82) | 8.41 (8.11 to 8.74) | 8.33 (7.99 to 8.69) | 8.61 (8.31 to 8.90) | 8.56 (8.27 to 8.86) | 8.48 (8.19 to 8.79) | 8.38 (8.06 to 8.72) |
| M2 | 8.61 (8.31 to 8.91) | 8.58 (8.29 to 8.88) | 8.50 (8.21 to 8.80) | 8.39 (8.08 to 8.73) | 8.61 (8.31 to 8.91) | 8.58 (8.29 to 8.88) | 8.50 (8.21 to 8.80) | 8.39 (8.08 to 8.73) |
| M3 | 8.63 (8.33 to 8.93) | 8.55 (8.26 to 8.85) | 8.44 (8.14 to 8.76) | 8.34 (8.01 to 8.70) | 8.65 (8.35 to 8.95) | 8.61 (8.31 to 8.90) | 8.52 (8.23 to 8.82) | 8.40 (8.09 to 8.73) |
| M4 | 8.65 (8.35 to 8.96) | 8.62 (8.32 to 8.92) | 8.52 (8.24 to 8.82) | 8.40 (8.10 to 8.73) | 8.66 (8.35 to 8.96) | 8.62 (8.33 to 8.92) | 8.53 (8.24 to 8.83) | 8.41 (8.11 to 8.74) |
| M5 | 8.65 (8.35 to 8.95) | 8.56 (8.27 to 8.86) | 8.44 (8.15 to 8.76) | 8.34 (8.01 to 8.70) | 8.67 (8.37 to 8.98) | 8.62 (8.33 to 8.92) | 8.53 (8.24 to 8.82) | 8.41 (8.11 to 8.73) |
| M6 | 8.67 (8.38 to 8.98) | 8.64 (8.35 to 8.94) | 8.55 (8.26 to 8.84) | 8.42 (8.12 to 8.74) | 8.67 (8.38 to 8.98) | 8.64 (8.35 to 8.94) | 8.55 (8.26 to 8.84) | 8.42 (8.12 to 8.74) |
| M7 | 8.69 (8.38 to 9.01) | 8.60 (8.31 to 8.90) | 8.47 (8.18 to 8.77) | 8.35 (8.03 to 8.70) | 8.72 (8.40 to 9.04) | 8.66 (8.37 to 8.97) | 8.56 (8.27 to 8.86) | 8.42 (8.13 to 8.74) |
| N1 | (2) | (2) | (2) | (2) | (2) | (2) | (2) | (2) |
| N2 | (2) | (2) | (2) | (2) | (2) | (2) | (2) | (2) |
| N3 | 8.64 (8.34 to 8.93) | 8.56 (8.26 to 8.85) | 8.44 (8.14 to 8.76) | 8.34 (8.01 to 8.70) | 8.66 (8.36 to 8.96) | 8.61 (8.32 to 8.91) | 8.52 (8.23 to 8.82) | 8.40 (8.10 to 8.73) |
| N4 | 8.66 (8.36 to 8.96) | 8.62 (8.32 to 8.92) | 8.53 (8.24 to 8.82) | 8.40 (8.10 to 8.73) | 8.66 (8.36 to 8.96) | 8.63 (8.33 to 8.93) | 8.54 (8.25 to 8.83) | 8.41 (8.11 to 8.74) |
| N5 | 8.65 (8.35 to 8.95) | 8.57 (8.28 to 8.87) | 8.45 (8.15 to 8.76) | 8.35 (8.02 to 8.70) | 8.68 (8.38 to 8.98) | 8.63 (8.34 to 8.93) | 8.53 (8.25 to 8.83) | 8.41 (8.11 to 8.74) |
| N6 | 8.68 (8.38 to 8.99) | 8.65 (8.35 to 8.95) | 8.55 (8.26 to 8.85) | 8.42 (8.12 to 8.74) | 8.68 (8.38 to 8.99) | 8.65 (8.35 to 8.95) | 8.55 (8.26 to 8.85) | 8.42 (8.12 to 8.74) |
| N7 | 8.71 (8.39 to 9.02) | 8.61 (8.31 to 8.91) | 8.47 (8.19 to 8.78) | 8.36 (8.04 to 8.70) | 8.73 (8.40 to 9.06) | 8.67 (8.38 to 8.98) | 8.57 (8.28 to 8.86) | 8.43 (8.13 to 8.75) |
| T1 | 8.54 (8.25 to 8.83) | (1) | (1) | (1) | (1) | (1) | (1) | (1) |
| T2 | 8.58 (8.29 to 8.88) | (1) | (1) | (1) | (1) | (1) | (1) | (1) |
| T3 | 8.56 (8.27 to 8.85) | (1) | (1) | (1) | (1) | (1) | (1) | (1) |
| T4 | 8.60 (8.31 to 8.90) | (1) | (1) | (1) | (1) | (1) | (1) | (1) |
| T5 | 8.64 (8.34 to 8.94) | 8.61 (8.33 to 8.91) | 8.59 (8.30 to 8.88) | 8.56 (8.27 to 8.86) | 8.64 (8.35 to 8.94) | 8.63 (8.34 to 8.93) | 8.61 (8.32 to 8.91) | 8.58 (8.29 to 8.88) |
| T6 | 8.61 (8.31 to 8.90) | 8.59 (8.30 to 8.89) | 8.57 (8.28 to 8.87) | 8.55 (8.27 to 8.85) | 8.61 (8.32 to 8.91) | 8.60 (8.31 to 8.90) | 8.59 (8.30 to 8.88) | 8.57 (8.27 to 8.86) |
| T7 | 8.70 (8.39 to 9.02) | 8.67 (8.37 to 8.97) | 8.62 (8.33 to 8.92) | 8.58 (8.29 to 8.88) | 8.71 (8.40 to 9.03) | 8.69 (8.38 to 9.00) | 8.65 (8.36 to 8.96) | 8.60 (8.31 to 8.91) |
| T8 | 8.64 (8.35 to 8.95) | 8.63 (8.34 to 8.94) | 8.61 (8.32 to 8.91) | 8.58 (8.29 to 8.88) | 8.64 (8.35 to 8.95) | 8.63 (8.34 to 8.94) | 8.61 (8.32 to 8.91) | 8.58 (8.29 to 8.88) |
| T9 | 8.71 (8.39 to 9.03) | 8.69 (8.38 to 9.00) | 8.64 (8.35 to 8.95) | 8.60 (8.30 to 8.90) | 8.71 (8.40 to 9.04) | 8.70 (8.39 to 9.01) | 8.66 (8.36 to 8.96) | 8.61 (8.31 to 8.91) |
| P1 | (1)(2) | (1) | (1) | (1) | (1) | (1) | (1) | (1) |
| P2 | 8.65 (8.36 to 8.96) | (1) | (1) | (1) | (1) | (1) | (1) | (1) |
| P3 | 8.62 (8.33 to 8.92) | (1) | (1) | (1) | (1) | (1) | (1) | (1) |
| P4 | 8.74 (8.41 to 9.06) | (1) | (1) | (1) | (1) | (1) | (1) | (1) |
| P5 | 8.65 (8.35 to 8.95) | 8.62 (8.34 to 8.92) | 8.59 (8.30 to 8.89) | 8.57 (8.28 to 8.86) | 8.65 (8.36 to 8.96) | 8.64 (8.35 to 8.94) | 8.61 (8.33 to 8.91) | 8.58 (8.29 to 8.88) |
| P6 | 8.62 (8.32 to 8.91) | 8.60 (8.31 to 8.90) | 8.58 (8.29 to 8.87) | 8.56 (8.27 to 8.85) | 8.62 (8.33 to 8.91) | 8.61 (8.32 to 8.90) | 8.59 (8.30 to 8.89) | 8.57 (8.28 to 8.86) |
| P7 | 8.72 (8.40 to 9.05) | 8.69 (8.38 to 8.99) | 8.63 (8.34 to 8.93) | 8.58 (8.30 to 8.88) | 8.73 (8.41 to 9.06) | 8.71 (8.40 to 9.02) | 8.67 (8.37 to 8.98) | 8.61 (8.32 to 8.91) |
| P8 | 8.65 (8.36 to 8.96) | 8.64 (8.35 to 8.95) | 8.62 (8.33 to 8.92) | 8.59 (8.29 to 8.89) | 8.65 (8.36 to 8.96) | 8.64 (8.35 to 8.95) | 8.62 (8.33 to 8.92) | 8.59 (8.29 to 8.89) |
| P9 | 8.73 (8.40 to 9.05) | 8.71 (8.39 to 9.02) | 8.66 (8.36 to 8.96) | 8.60 (8.31 to 8.90) | 8.73 (8.41 to 9.06) | 8.72 (8.40 to 9.03) | 8.67 (8.38 to 8.98) | 8.62 (8.33 to 8.91) |
| (1) These strategies do not include MPMRI and therefore the results do not vary by MPMRI definition and cut-off.   (2) These strategies do not include TRUS-guided and therefore the results do not vary by TRUSB definition. | | | | | | | | |

Supplementary Table 23 – Lifetime costs in pound by strategy, mean (95% confidence intervals), TRUSB definition 1, MPMRI definition 1 and 2, MPMRI cut-off 2-5

| **Strategy** | **112** | **113** | **114** | **115** | **122** | **123** | **124** | **125** |
| --- | --- | --- | --- | --- | --- | --- | --- | --- |
| M1 | £4260 (£3828 to £4755) | £3995 (£3538 to £4502) | £3714 (£3235 to £4291) | £3497 (£3007 to £4115) | £4329 (£3900 to £4814) | £4156 (£3718 to £4659) | £3897 (£3444 to £4439) | £3627 (£3156 to £4217) |
| M2 | £4351 (£3926 to £4834) | £4227 (£3787 to £4724) | £3941 (£3484 to £4466) | £3651 (£3184 to £4239) | £4351 (£3926 to £4834) | £4227 (£3787 to £4724) | £3941 (£3484 to £4466) | £3651 (£3184 to £4239) |
| M3 | £4817 (£4401 to £5258) | £4430 (£4015 to £4863) | £3985 (£3541 to £4491) | £3633 (£3158 to £4211) | £4917 (£4507 to £5359) | £4672 (£4261 to £5104) | £4282 (£3862 to £4744) | £3848 (£3409 to £4368) |
| M4 | £4908 (£4499 to £5351) | £4662 (£4250 to £5088) | £4212 (£3786 to £4702) | £3788 (£3340 to £4328) | £4938 (£4530 to £5384) | £4743 (£4332 to £5171) | £4326 (£3909 to £4779) | £3872 (£3431 to £4383) |
| M5 | £4678 (£4263 to £5118) | £4268 (£3836 to £4743) | £3867 (£3407 to £4403) | £3571 (£3090 to £4172) | £4787 (£4372 to £5227) | £4511 (£4091 to £4953) | £4121 (£3681 to £4619) | £3747 (£3286 to £4299) |
| M6 | £4824 (£4411 to £5267) | £4624 (£4204 to £5060) | £4184 (£3755 to £4667) | £3780 (£3330 to £4332) | £4824 (£4411 to £5267) | £4624 (£4204 to £5060) | £4184 (£3755 to £4667) | £3780 (£3330 to £4332) |
| M7 | £5235 (£4809 to £5728) | £4703 (£4292 to £5146) | £4139 (£3704 to £4633) | £3707 (£3239 to £4269) | £5374 (£4949 to £5879) | £5027 (£4609 to £5512) | £4507 (£4083 to £4941) | £3968 (£3534 to £4454) |
| N1 | £5935 (£5514 to £6457) | £5178 (£4761 to £5640) | £4421 (£3976 to £4920) | £3849 (£3376 to £4412) | £6135 (£5696 to £6659) | £5630 (£5208 to £6129) | £4906 (£4479 to £5357) | £4189 (£3756 to £4689) |
| N2 | £6201 (£5764 to £6740) | £5837 (£5403 to £6349) | £5024 (£4608 to £5489) | £4252 (£3813 to £4749) | £6201 (£5764 to £6740) | £5837 (£5403 to £6349) | £5024 (£4608 to £5489) | £4252 (£3813 to £4749) |
| N3 | £5093 (£4684 to £5542) | £4638 (£4227 to £5071) | £4112 (£3668 to £4629) | £3696 (£3220 to £4264) | £5210 (£4785 to £5670) | £4923 (£4522 to £5357) | £4464 (£4043 to £4925) | £3949 (£3510 to £4475) |
| N4 | £5184 (£4772 to £5639) | £4870 (£4462 to £5293) | £4340 (£3919 to £4820) | £3850 (£3403 to £4389) | £5231 (£4811 to £5694) | £4995 (£4591 to £5434) | £4508 (£4088 to £4956) | £3973 (£3532 to £4490) |
| N5 | £5122 (£4720 to £5565) | £4484 (£4052 to £4949) | £3962 (£3502 to £4488) | £3611 (£3128 to £4208) | £5297 (£4895 to £5736) | £4842 (£4422 to £5277) | £4270 (£3826 to £4759) | £3812 (£3346 to £4361) |
| N6 | £5363 (£4960 to £5807) | £5027 (£4618 to £5469) | £4352 (£3921 to £4842) | £3850 (£3405 to £4393) | £5363 (£4960 to £5807) | £5027 (£4618 to £5469) | £4352 (£3921 to £4842) | £3850 (£3405 to £4393) |
| N7 | £5955 (£5536 to £6477) | £5126 (£4711 to £5583) | £4360 (£3915 to £4850) | £3809 (£3342 to £4360) | £6177 (£5755 to £6692) | £5609 (£5192 to £6108) | £4837 (£4410 to £5281) | £4134 (£3703 to £4630) |
| T1 | £4038 (£3602 to £4537) | (1) | (1) | (1) | (1) | (1) | (1) | (1) |
| T2 | £4324 (£3900 to £4798) | (1) | (1) | (1) | (1) | (1) | (1) | (1) |
| T3 | £4317 (£3884 to £4772) | (1) | (1) | (1) | (1) | (1) | (1) | (1) |
| T4 | £4603 (£4174 to £5044) | (1) | (1) | (1) | (1) | (1) | (1) | (1) |
| T5 | £4604 (£4192 to £5036) | £4451 (£4040 to £4901) | £4315 (£3898 to £4783) | £4221 (£3801 to £4702) | £4646 (£4235 to £5077) | £4539 (£4122 to £4977) | £4397 (£3983 to £4851) | £4276 (£3864 to £4750) |
| T6 | £4721 (£4303 to £5150) | £4580 (£4180 to £5007) | £4394 (£3995 to £4838) | £4242 (£3833 to £4711) | £4755 (£4331 to £5190) | £4673 (£4266 to £5099) | £4523 (£4127 to £4954) | £4336 (£3934 to £4795) |
| T7 | £5286 (£4856 to £5778) | £4992 (£4574 to £5454) | £4671 (£4268 to £5108) | £4424 (£4027 to £4883) | £5363 (£4939 to £5868) | £5173 (£4755 to £5664) | £4882 (£4480 to £5327) | £4574 (£4176 to £5006) |
| T8 | £4661 (£4254 to £5089) | £4582 (£4169 to £5011) | £4418 (£4006 to £4869) | £4287 (£3874 to £4754) | £4661 (£4254 to £5089) | £4582 (£4169 to £5011) | £4418 (£4006 to £4869) | £4287 (£3874 to £4754) |
| T9 | £5343 (£4916 to £5844) | £5124 (£4709 to £5594) | £4774 (£4365 to £5211) | £4490 (£4096 to £4933) | £5378 (£4952 to £5879) | £5217 (£4799 to £5707) | £4903 (£4497 to £5351) | £4585 (£4187 to £5014) |
| P1 | £6035 (£5596 to £6572) | (1) | (1) | (1) | (1) | (1) | (1) | (1) |
| P2 | £5118 (£4715 to £5547) | (1) | (1) | (1) | (1) | (1) | (1) | (1) |
| P3 | £5073 (£4668 to £5531) | (1) | (1) | (1) | (1) | (1) | (1) | (1) |
| P4 | £6153 (£5728 to £6688) | (1) | (1) | (1) | (1) | (1) | (1) | (1) |
| P5 | £5058 (£4650 to £5489) | £4681 (£4257 to £5117) | £4421 (£4006 to £4896) | £4267 (£3846 to £4748) | £5164 (£4761 to £5598) | £4883 (£4472 to £5308) | £4561 (£4148 to £5017) | £4352 (£3936 to £4822) |
| P6 | £5046 (£4643 to £5491) | £4819 (£4418 to £5246) | £4539 (£4133 to £4985) | £4313 (£3902 to £4786) | £5102 (£4693 to £5545) | £4965 (£4567 to £5395) | £4731 (£4329 to £5163) | £4451 (£4056 to £4904) |
| P7 | £6065 (£5647 to £6581) | £5462 (£5049 to £5930) | £4921 (£4515 to £5358) | £4541 (£4139 to £4988) | £6227 (£5804 to £6751) | £5810 (£5398 to £6325) | £5254 (£4854 to £5713) | £4764 (£4364 to £5185) |
| P8 | £5207 (£4807 to £5636) | £4997 (£4585 to £5428) | £4601 (£4185 to £5046) | £4368 (£3951 to £4835) | £5207 (£4807 to £5636) | £4997 (£4585 to £5428) | £4601 (£4185 to £5046) | £4368 (£3951 to £4835) |
| P9 | £6230 (£5814 to £6749) | £5809 (£5404 to £6299) | £5113 (£4719 to £5570) | £4643 (£4245 to £5084) | £6277 (£5858 to £6808) | £5936 (£5528 to £6448) | £5297 (£4902 to £5763) | £4780 (£4380 to £5205) |
| (1) These strategies do not include MPMRI and therefore the results do not vary by MPMRI definition and cut-off | | | | | | | | |

Supplementary Table 24 – Lifetime costs in pound by strategy, mean (95% confidence intervals), TRUSB definition 2, MPMRI definition 1 and 2, MPMRI cut-off 2-5

| **Strategy** | **212** | **213** | **214** | **215** | **222** | **223** | **224** | **225** |
| --- | --- | --- | --- | --- | --- | --- | --- | --- |
| M1 | £4706 (£4282 to £5165) | £4347 (£3918 to £4807) | £3934 (£3482 to £4458) | £3608 (£3137 to £4191) | £4798 (£4370 to £5265) | £4571 (£4144 to £5042) | £4209 (£3776 to £4690) | £3807 (£3367 to £4337) |
| M2 | £4825 (£4395 to £5300) | £4665 (£4249 to £5119) | £4274 (£3836 to £4744) | £3844 (£3400 to £4370) | £4825 (£4395 to £5300) | £4665 (£4249 to £5119) | £4274 (£3836 to £4744) | £3844 (£3400 to £4370) |
| M3 | £4878 (£4469 to £5324) | £4478 (£4071 to £4914) | £4015 (£3572 to £4519) | £3648 (£3180 to £4218) | £4981 (£4562 to £5451) | £4729 (£4321 to £5173) | £4325 (£3915 to £4774) | £3872 (£3438 to £4380) |
| M4 | £4997 (£4582 to £5460) | £4796 (£4382 to £5248) | £4355 (£3939 to £4804) | £3884 (£3448 to £4395) | £5008 (£4590 to £5473) | £4823 (£4404 to £5275) | £4390 (£3979 to £4833) | £3909 (£3477 to £4415) |
| M5 | £5056 (£4649 to £5515) | £4566 (£4148 to £5003) | £4054 (£3622 to £4554) | £3665 (£3202 to £4238) | £5185 (£4769 to £5658) | £4863 (£4449 to £5319) | £4386 (£3966 to £4834) | £3900 (£3465 to £4403) |
| M6 | £5226 (£4811 to £5698) | £4995 (£4589 to £5451) | £4466 (£4051 to £4906) | £3943 (£3511 to £4439) | £5226 (£4811 to £5698) | £4995 (£4589 to £5451) | £4466 (£4051 to £4906) | £3943 (£3511 to £4439) |
| M7 | £5228 (£4823 to £5732) | £4698 (£4291 to £5139) | £4135 (£3713 to £4623) | £3705 (£3242 to £4261) | £5367 (£4947 to £5876) | £5021 (£4612 to £5496) | £4502 (£4094 to £4937) | £3965 (£3539 to £4455) |
| N1 | (2) | (2) | (2) | (2) | (2) | (2) | (2) | (2) |
| N2 | (2) | (2) | (2) | (2) | (2) | (2) | (2) | (2) |
| N3 | £4978 (£4568 to £5438) | £4547 (£4142 to £4983) | £4055 (£3615 to £4554) | £3667 (£3196 to £4232) | £5089 (£4664 to £5565) | £4817 (£4409 to £5266) | £4384 (£3975 to £4829) | £3903 (£3466 to £4410) |
| N4 | £5097 (£4681 to £5572) | £4865 (£4454 to £5317) | £4395 (£3981 to £4840) | £3903 (£3463 to £4411) | £5115 (£4697 to £5596) | £4910 (£4487 to £5365) | £4449 (£4036 to £4886) | £3939 (£3508 to £4442) |
| N5 | £5469 (£5062 to £5944) | £4757 (£4339 to £5202) | £4133 (£3697 to £4637) | £3697 (£3238 to £4267) | £5661 (£5251 to £6132) | £5165 (£4745 to £5626) | £4513 (£4104 to £4960) | £3952 (£3514 to £4457) |
| N6 | £5732 (£5326 to £6204) | £5368 (£4960 to £5826) | £4611 (£4197 to £5055) | £3999 (£3567 to £4497) | £5732 (£5326 to £6204) | £5368 (£4960 to £5826) | £4611 (£4197 to £5055) | £3999 (£3567 to £4497) |
| N7 | £5742 (£5326 to £6247) | £4958 (£4552 to £5408) | £4255 (£3821 to £4730) | £3756 (£3291 to £4304) | £5952 (£5522 to £6464) | £5410 (£5002 to £5889) | £4688 (£4276 to £5114) | £4047 (£3615 to £4534) |
| T1 | £4423 (£4016 to £4852) | (1) | (1) | (1) | (1) | (1) | (1) | (1) |
| T2 | £4762 (£4351 to £5196) | (1) | (1) | (1) | (1) | (1) | (1) | (1) |
| T3 | £4548 (£4142 to £4967) | (1) | (1) | (1) | (1) | (1) | (1) | (1) |
| T4 | £4888 (£4485 to £5306) | (1) | (1) | (1) | (1) | (1) | (1) | (1) |
| T5 | £4989 (£4585 to £5435) | £4835 (£4428 to £5275) | £4699 (£4289 to £5132) | £4605 (£4197 to £5045) | £5030 (£4625 to £5479) | £4923 (£4519 to £5370) | £4782 (£4374 to £5220) | £4661 (£4259 to £5097) |
| T6 | £4716 (£4310 to £5149) | £4652 (£4249 to £5081) | £4574 (£4180 to £5003) | £4511 (£4117 to £4939) | £4732 (£4327 to £5166) | £4693 (£4289 to £5127) | £4628 (£4226 to £5057) | £4550 (£4156 to £4982) |
| T7 | £5282 (£4866 to £5787) | £5064 (£4656 to £5544) | £4851 (£4446 to £5296) | £4694 (£4290 to £5131) | £5339 (£4918 to £5846) | £5194 (£4780 to £5682) | £4987 (£4578 to £5455) | £4787 (£4387 to £5214) |
| T8 | £5045 (£4638 to £5495) | £4967 (£4565 to £5413) | £4803 (£4394 to £5240) | £4671 (£4268 to £5108) | £5045 (£4638 to £5495) | £4967 (£4565 to £5413) | £4803 (£4394 to £5240) | £4671 (£4268 to £5108) |
| T9 | £5339 (£4920 to £5843) | £5196 (£4788 to £5691) | £4954 (£4550 to £5417) | £4760 (£4358 to £5192) | £5354 (£4936 to £5866) | £5237 (£4827 to £5734) | £5008 (£4601 to £5478) | £4798 (£4400 to £5227) |
| P1 | (1)(2) | (1) | (1) | (1) | (1) | (1) | (1) | (1) |
| P2 | £5502 (£5095 to £5962) | (1) | (1) | (1) | (1) | (1) | (1) | (1) |
| P3 | £4888 (£4489 to £5334) | (1) | (1) | (1) | (1) | (1) | (1) | (1) |
| P4 | £5968 (£5550 to £6490) | (1) | (1) | (1) | (1) | (1) | (1) | (1) |
| P5 | £5442 (£5039 to £5900) | £5066 (£4649 to £5507) | £4805 (£4382 to £5244) | £4651 (£4247 to £5092) | £5548 (£5151 to £6011) | £5268 (£4853 to £5718) | £4946 (£4531 to £5388) | £4736 (£4331 to £5181) |
| P6 | £4874 (£4475 to £5316) | £4759 (£4362 to £5193) | £4637 (£4241 to £5059) | £4540 (£4145 to £4967) | £4903 (£4504 to £5345) | £4830 (£4429 to £5273) | £4719 (£4322 to £5150) | £4597 (£4200 to £5024) |
| P7 | £5894 (£5479 to £6409) | £5402 (£4982 to £5899) | £5019 (£4614 to £5470) | £4769 (£4363 to £5203) | £6028 (£5612 to £6555) | £5675 (£5266 to £6207) | £5242 (£4837 to £5708) | £4910 (£4512 to £5343) |
| P8 | £5591 (£5187 to £6053) | £5381 (£4975 to £5832) | £4986 (£4566 to £5428) | £4753 (£4345 to £5192) | £5591 (£5187 to £6053) | £5381 (£4975 to £5832) | £4986 (£4566 to £5428) | £4753 (£4345 to £5192) |
| P9 | £6058 (£5651 to £6575) | £5750 (£5338 to £6261) | £5211 (£4807 to £5671) | £4871 (£4469 to £5306) | £6077 (£5666 to £6599) | £5801 (£5391 to £6320) | £5285 (£4877 to £5758) | £4926 (£4529 to £5358) |
| (1) These strategies do not include MPMRI and therefore the results do not vary by MPMRI definition and cut-off.   (2) These strategies do not include TRUS-guided and therefore the results do not vary by TRUSB definition. | | | | | | | | |

Supplementary Table 25 – Lifetime costs by strategy, mean (95% confidence intervals), TRUSB definition 1, MPMRI definition 1 and 2, MPMRI cut-off 2-5

| **Strategy** | **112** | **113** | **114** | **115** | **122** | **123** | **124** | **125** |
| --- | --- | --- | --- | --- | --- | --- | --- | --- |
| M1 | 5027 (4517 to 5610) | 4714 (4175 to 5312) | 4382 (3817 to 5064) | 4126 (3549 to 4855) | 5109 (4602 to 5681) | 4904 (4387 to 5498) | 4599 (4065 to 5238) | 4280 (3725 to 4975) |
| M2 | 5134 (4633 to 5705) | 4988 (4469 to 5575) | 4651 (4111 to 5270) | 4309 (3757 to 5002) | 5134 (4633 to 5705) | 4988 (4469 to 5575) | 4651 (4111 to 5270) | 4309 (3757 to 5002) |
| M3 | 5684 (5193 to 6205) | 5227 (4738 to 5739) | 4702 (4178 to 5300) | 4287 (3727 to 4969) | 5801 (5318 to 6323) | 5513 (5028 to 6022) | 5053 (4557 to 5598) | 4540 (4022 to 5154) |
| M4 | 5792 (5309 to 6314) | 5501 (5015 to 6004) | 4971 (4468 to 5548) | 4469 (3941 to 5107) | 5827 (5345 to 6353) | 5597 (5111 to 6102) | 5105 (4613 to 5639) | 4568 (4049 to 5172) |
| M5 | 5520 (5030 to 6039) | 5036 (4526 to 5597) | 4564 (4020 to 5195) | 4214 (3646 to 4923) | 5649 (5159 to 6168) | 5323 (4827 to 5844) | 4863 (4343 to 5450) | 4422 (3877 to 5073) |
| M6 | 5692 (5204 to 6215) | 5456 (4961 to 5971) | 4937 (4431 to 5507) | 4461 (3929 to 5112) | 5692 (5204 to 6215) | 5456 (4961 to 5971) | 4937 (4431 to 5507) | 4461 (3929 to 5112) |
| M7 | 6178 (5675 to 6759) | 5550 (5065 to 6072) | 4884 (4371 to 5467) | 4374 (3822 to 5037) | 6342 (5840 to 6938) | 5932 (5438 to 6504) | 5318 (4818 to 5830) | 4682 (4170 to 5256) |
| N1 | 7003 (6507 to 7619) | 6110 (5618 to 6655) | 5217 (4691 to 5806) | 4541 (3983 to 5206) | 7239 (6721 to 7858) | 6643 (6146 to 7232) | 5789 (5285 to 6321) | 4943 (4432 to 5533) |
| N2 | 7317 (6801 to 7953) | 6887 (6375 to 7492) | 5929 (5437 to 6477) | 5017 (4500 to 5604) | 7317 (6801 to 7953) | 6887 (6375 to 7492) | 5929 (5437 to 6477) | 5017 (4500 to 5604) |
| N3 | 6010 (5527 to 6539) | 5473 (4988 to 5984) | 4852 (4328 to 5463) | 4361 (3800 to 5031) | 6147 (5646 to 6691) | 5810 (5336 to 6322) | 5268 (4771 to 5811) | 4660 (4142 to 5280) |
| N4 | 6117 (5631 to 6654) | 5746 (5265 to 6246) | 5121 (4624 to 5687) | 4543 (4015 to 5179) | 6173 (5677 to 6718) | 5894 (5417 to 6412) | 5320 (4824 to 5849) | 4688 (4167 to 5299) |
| N5 | 6044 (5570 to 6566) | 5291 (4781 to 5840) | 4675 (4133 to 5296) | 4260 (3691 to 4965) | 6250 (5776 to 6769) | 5713 (5218 to 6227) | 5039 (4514 to 5616) | 4498 (3949 to 5146) |
| N6 | 6328 (5853 to 6852) | 5932 (5450 to 6453) | 5135 (4627 to 5713) | 4543 (4018 to 5183) | 6328 (5853 to 6852) | 5932 (5450 to 6453) | 5135 (4627 to 5713) | 4543 (4018 to 5183) |
| N7 | 7027 (6532 to 7643) | 6049 (5559 to 6588) | 5145 (4620 to 5723) | 4495 (3943 to 5145) | 7289 (6791 to 7896) | 6619 (6126 to 7208) | 5708 (5203 to 6232) | 4878 (4370 to 5464) |
| T1 | 4765 (4251 to 5354) | (1) | (1) | (1) | (1) | (1) | (1) | (1) |
| T2 | 5102 (4602 to 5661) | (1) | (1) | (1) | (1) | (1) | (1) | (1) |
| T3 | 5094 (4584 to 5631) | (1) | (1) | (1) | (1) | (1) | (1) | (1) |
| T4 | 5431 (4925 to 5951) | (1) | (1) | (1) | (1) | (1) | (1) | (1) |
| T5 | 5433 (4947 to 5942) | 5252 (4767 to 5783) | 5091 (4600 to 5644) | 4980 (4485 to 5549) | 5482 (4997 to 5991) | 5356 (4864 to 5873) | 5189 (4700 to 5724) | 5046 (4559 to 5605) |
| T6 | 5570 (5078 to 6077) | 5404 (4933 to 5908) | 5185 (4714 to 5709) | 5006 (4523 to 5559) | 5611 (5111 to 6124) | 5514 (5034 to 6017) | 5337 (4869 to 5846) | 5117 (4642 to 5658) |
| T7 | 6238 (5730 to 6818) | 5891 (5397 to 6436) | 5511 (5037 to 6027) | 5221 (4752 to 5761) | 6328 (5828 to 6924) | 6104 (5611 to 6683) | 5761 (5286 to 6285) | 5397 (4928 to 5907) |
| T8 | 5500 (5020 to 6005) | 5407 (4919 to 5913) | 5214 (4728 to 5746) | 5058 (4571 to 5609) | 5500 (5020 to 6005) | 5407 (4919 to 5913) | 5214 (4728 to 5746) | 5058 (4571 to 5609) |
| T9 | 6305 (5801 to 6896) | 6046 (5557 to 6601) | 5634 (5151 to 6149) | 5299 (4834 to 5821) | 6346 (5843 to 6938) | 6156 (5663 to 6734) | 5785 (5307 to 6314) | 5410 (4940 to 5916) |
| P1 | 7121 (6604 to 7755) | (1) | (1) | (1) | (1) | (1) | (1) | (1) |
| P2 | 6039 (5564 to 6546) | (1) | (1) | (1) | (1) | (1) | (1) | (1) |
| P3 | 5987 (5509 to 6526) | (1) | (1) | (1) | (1) | (1) | (1) | (1) |
| P4 | 7260 (6759 to 7892) | (1) | (1) | (1) | (1) | (1) | (1) | (1) |
| P5 | 5969 (5487 to 6476) | 5524 (5024 to 6039) | 5216 (4727 to 5777) | 5035 (4538 to 5603) | 6093 (5618 to 6605) | 5762 (5277 to 6264) | 5382 (4895 to 5920) | 5135 (4644 to 5690) |
| P6 | 5954 (5479 to 6479) | 5686 (5213 to 6191) | 5356 (4876 to 5883) | 5089 (4605 to 5647) | 6020 (5538 to 6543) | 5859 (5389 to 6367) | 5583 (5108 to 6092) | 5252 (4786 to 5787) |
| P7 | 7157 (6664 to 7766) | 6445 (5957 to 6997) | 5807 (5327 to 6322) | 5359 (4884 to 5886) | 7348 (6849 to 7967) | 6856 (6370 to 7464) | 6200 (5728 to 6741) | 5621 (5150 to 6118) |
| P8 | 6144 (5673 to 6650) | 5897 (5410 to 6404) | 5430 (4938 to 5954) | 5155 (4662 to 5705) | 6144 (5673 to 6650) | 5897 (5410 to 6404) | 5430 (4938 to 5954) | 5155 (4662 to 5705) |
| P9 | 7351 (6860 to 7964) | 6855 (6376 to 7432) | 6034 (5569 to 6573) | 5479 (5009 to 6000) | 7406 (6913 to 8033) | 7005 (6523 to 7609) | 6251 (5784 to 6800) | 5641 (5169 to 6142) |
| (1) These strategies do not include MPMRI and therefore the results do not vary by MPMRI definition and cut-off | | | | | | | | |

Supplementary Table 26 – Lifetime costs in pound by strategy, mean (95% confidence intervals), TRUSB definition 2, MPMRI definition 1 and 2, MPMRI cut-off 2-5

| **Strategy** | **212** | **213** | **214** | **215** | **222** | **223** | **224** | **225** |
| --- | --- | --- | --- | --- | --- | --- | --- | --- |
| M1 | 5553 (5052 to 6095) | 5129 (4623 to 5672) | 4642 (4109 to 5261) | 4258 (3702 to 4946) | 5662 (5156 to 6213) | 5394 (4890 to 5950) | 4967 (4456 to 5535) | 4492 (3973 to 5118) |
| M2 | 5694 (5186 to 6254) | 5504 (5014 to 6041) | 5043 (4527 to 5598) | 4535 (4012 to 5157) | 5694 (5186 to 6254) | 5504 (5014 to 6041) | 5043 (4527 to 5598) | 4535 (4012 to 5157) |
| M3 | 5756 (5273 to 6283) | 5284 (4804 to 5799) | 4738 (4215 to 5332) | 4305 (3753 to 4977) | 5878 (5384 to 6433) | 5581 (5099 to 6105) | 5104 (4620 to 5633) | 4569 (4056 to 5168) |
| M4 | 5897 (5406 to 6443) | 5660 (5171 to 6192) | 5139 (4649 to 5669) | 4583 (4068 to 5186) | 5909 (5416 to 6458) | 5691 (5197 to 6225) | 5180 (4695 to 5702) | 4612 (4103 to 5210) |
| M5 | 5966 (5485 to 6508) | 5388 (4895 to 5904) | 4784 (4273 to 5374) | 4325 (3779 to 5000) | 6118 (5628 to 6677) | 5738 (5249 to 6276) | 5175 (4680 to 5705) | 4601 (4089 to 5196) |
| M6 | 6166 (5677 to 6723) | 5894 (5415 to 6432) | 5270 (4780 to 5790) | 4653 (4143 to 5238) | 6166 (5677 to 6723) | 5894 (5415 to 6432) | 5270 (4780 to 5790) | 4653 (4143 to 5238) |
| M7 | 6169 (5691 to 6764) | 5543 (5064 to 6064) | 4880 (4381 to 5455) | 4372 (3826 to 5027) | 6333 (5837 to 6933) | 5924 (5442 to 6486) | 5312 (4831 to 5826) | 4678 (4176 to 5257) |
| N1 | (2) | (2) | (2) | (2) | (2) | (2) | (2) | (2) |
| N2 | (2) | (2) | (2) | (2) | (2) | (2) | (2) | (2) |
| N3 | 5874 (5391 to 6417) | 5366 (4887 to 5880) | 4785 (4265 to 5373) | 4327 (3771 to 4994) | 6005 (5503 to 6567) | 5684 (5202 to 6214) | 5173 (4691 to 5699) | 4605 (4090 to 5204) |
| N4 | 6015 (5523 to 6575) | 5741 (5256 to 6273) | 5187 (4698 to 5711) | 4605 (4086 to 5205) | 6036 (5543 to 6603) | 5794 (5295 to 6331) | 5249 (4763 to 5766) | 4649 (4140 to 5241) |
| N5 | 6454 (5973 to 7015) | 5614 (5120 to 6139) | 4877 (4363 to 5472) | 4362 (3821 to 5035) | 6681 (6197 to 7236) | 6095 (5600 to 6639) | 5326 (4843 to 5853) | 4663 (4147 to 5259) |
| N6 | 6763 (6285 to 7321) | 6334 (5853 to 6874) | 5440 (4952 to 5965) | 4719 (4209 to 5307) | 6763 (6285 to 7321) | 6334 (5853 to 6874) | 5440 (4952 to 5965) | 4719 (4209 to 5307) |
| N7 | 6775 (6284 to 7371) | 5850 (5371 to 6382) | 5020 (4508 to 5582) | 4432 (3884 to 5079) | 7023 (6516 to 7628) | 6384 (5902 to 6949) | 5531 (5046 to 6035) | 4776 (4266 to 5350) |
| T1 | 5219 (4739 to 5726) | (1) | (1) | (1) | (1) | (1) | (1) | (1) |
| T2 | 5619 (5134 to 6132) | (1) | (1) | (1) | (1) | (1) | (1) | (1) |
| T3 | 5367 (4887 to 5861) | (1) | (1) | (1) | (1) | (1) | (1) | (1) |
| T4 | 5768 (5292 to 6261) | (1) | (1) | (1) | (1) | (1) | (1) | (1) |
| T5 | 5887 (5410 to 6413) | 5705 (5225 to 6225) | 5545 (5061 to 6056) | 5434 (4953 to 5953) | 5936 (5458 to 6465) | 5809 (5333 to 6336) | 5642 (5161 to 6160) | 5499 (5026 to 6014) |
| T6 | 5565 (5086 to 6075) | 5490 (5014 to 5996) | 5398 (4932 to 5903) | 5323 (4858 to 5828) | 5584 (5105 to 6096) | 5538 (5062 to 6050) | 5461 (4987 to 5967) | 5369 (4904 to 5879) |
| T7 | 6233 (5742 to 6829) | 5976 (5494 to 6542) | 5724 (5246 to 6249) | 5539 (5062 to 6055) | 6301 (5803 to 6899) | 6128 (5640 to 6705) | 5884 (5403 to 6437) | 5649 (5177 to 6153) |
| T8 | 5954 (5473 to 6484) | 5861 (5387 to 6387) | 5667 (5185 to 6183) | 5512 (5037 to 6028) | 5954 (5473 to 6484) | 5861 (5387 to 6387) | 5667 (5185 to 6183) | 5512 (5037 to 6028) |
| T9 | 6299 (5806 to 6894) | 6132 (5650 to 6715) | 5846 (5369 to 6393) | 5616 (5142 to 6127) | 6318 (5824 to 6921) | 6180 (5696 to 6766) | 5909 (5430 to 6464) | 5662 (5192 to 6168) |
| P1 | (1)(2) | (1) | (1) | (1) | (1) | (1) | (1) | (1) |
| P2 | 6493 (6012 to 7035) | (1) | (1) | (1) | (1) | (1) | (1) | (1) |
| P3 | 5768 (5297 to 6294) | (1) | (1) | (1) | (1) | (1) | (1) | (1) |
| P4 | 7042 (6550 to 7658) | (1) | (1) | (1) | (1) | (1) | (1) | (1) |
| P5 | 6422 (5946 to 6962) | 5978 (5486 to 6498) | 5670 (5171 to 6188) | 5489 (5012 to 6009) | 6547 (6078 to 7093) | 6216 (5726 to 6748) | 5836 (5347 to 6358) | 5588 (5111 to 6113) |
| P6 | 5751 (5280 to 6273) | 5616 (5147 to 6128) | 5471 (5004 to 5969) | 5358 (4891 to 5861) | 5785 (5315 to 6308) | 5700 (5226 to 6222) | 5568 (5100 to 6077) | 5424 (4956 to 5928) |
| P7 | 6955 (6465 to 7563) | 6375 (5879 to 6961) | 5922 (5445 to 6455) | 5627 (5148 to 6140) | 7113 (6622 to 7735) | 6696 (6214 to 7324) | 6185 (5707 to 6735) | 5794 (5324 to 6305) |
| P8 | 6598 (6120 to 7142) | 6350 (5871 to 6882) | 5883 (5388 to 6405) | 5608 (5127 to 6127) | 6598 (6120 to 7142) | 6350 (5871 to 6882) | 5883 (5388 to 6405) | 5608 (5127 to 6127) |
| P9 | 7149 (6668 to 7759) | 6785 (6299 to 7388) | 6149 (5673 to 6691) | 5748 (5274 to 6262) | 7171 (6686 to 7786) | 6845 (6362 to 7458) | 6236 (5755 to 6794) | 5813 (5345 to 6322) |
| (1) These strategies do not include MPMRI and therefore the results do not vary by MPMRI definition and cut-off.   (2) These strategies do not include TRUS-guided and therefore the results do not vary by TRUSB definition. | | | | | | | | |

Supplementary Table 27 – Diagnostic strategies in the cost-effectiveness frontier for the base-case (using NHS reference costs), average (95% confidence interval)

| **Strategy** | **TRUS-guided definition** | **MPMRI definition** | **MPMRI cut-off** | **QALYs** | **Costs in pound** | **ICER, in pound** | **Costs in euro** | **ICER, in euro** | **Net health at** | | |
| --- | --- | --- | --- | --- | --- | --- | --- | --- | --- | --- | --- |
|  |  |  |  |  |  |  |  |  | **£13,000 (€15,398)/QALY** | **£20,000(€23,689)/QALY** | **£30,000(€35,534)/**  **QALY** |
| M1: MPMRI for all men; TRUSB in men suspicious of CS cancer | 1 | 1 | 5 | 8.29 (7.94 to 8.68) | £3497 (£3007 to £4115) |  | 4126 (3549 to 4855) |  | 8.024 | 8.118 | 8.176 |
|  | 2 | 1 | 5 | 8.33 (7.99 to 8.69) | £3608 (£3137 to £4191) | £3,081 | 4258 (3702 to 4946) | 3635 | 8.051 | 8.148 | 8.208 |
| M3: MPMRI for all men; TRUSB in men with suspicion on CS cancer; Men with NON-CS at 1st biopsy receive 2nd TRUSB | 2 | 1 | 5 | 8.34 (8.01 to 8.70) | £3648 (£3180 to £4218) | £3,630 | 4305 (3753 to 4977) | 4283 | 8.059 | 8.157 | 8.218 |
| M4: MPMRI for all men; TRUS-guided in men with suspicion of any cancer. Men with suspicion of CS cancer at MPMRI and in whom NON-CS cancer was detected at the 1st biopsy receive 2nd TRUSB. | 2 | 2 | 5 | 8.41 (8.11 to 8.74) | £3909 (£3477 to £4415) | £3,738 | 4612 (4103 to 5210) | 4411 | 8.109 | 8.214 | 8.279 |
| M7: MPMRI for all men; TRUSB in men with suspicion of CS cancer. Re-biopsy with TRUSB those in whom CS cancer was not detected | 2 | 2 | 5 | 8.42 (8.13 to 8.74) | £3965 (£3539 to £4455) | £3,867 | 4678 (4176 to 5257) | 4563 | 8.119 | 8.226 | 8.292 |
| M3: MPMRI for all men; TRUSB in men with suspicion on CS cancer;  Men with NON-CS at 1st biopsy receive 2nd TRUSB | 2 | 2 | 4 | 8.52 (8.23 to 8.82) | £4325 (£3915 to £4774) | £3,921 | 5104 (4620 to 5633) | 4626 | 8.183 | 8.300 | 8.372 |
| M4: MPMRI for all men; TRUS-guided in men with suspicion of any cancer. Men with suspicion of CS cancer at MPMRI and in whom NON-CS cancer was detected at the 1st biopsy receive 2nd TRUSB. | 2 | 2 | 4 | 8.53 (8.24 to 8.83) | £4390 (£3979 to £4833) | £4,031 | 5180 (4695 to 5702) | 4756 | 8.194 | 8.312 | 8.386 |
| M7: MPMRI for all men; TRUSB in men with suspicion of CS cancer. Re-biopsy with TRUSB those in whom CS cancer was not detected | 2 | 2 | 4 | 8.56 (8.27 to 8.86) | £4502 (£4094 to £4937) | £4,250 | 5312 (4831 to 5826) | 5015 | 8.212 | 8.333 | 8.408 |
| T6: TRUSB for all men; Men classified as NON-CS receive a MRI. Men with suspicion of CS cancer receive a 2nd TRUSB | 2 | 2 | 3 | 8.60 (8.31 to 8.90) | £4693 (£4289 to £5127) | £4,393 | 5538 (5062 to 6050) | 5184 | 8.241 | 8.367 | 8.445 |
|  | 2 | 2 | 2 | 8.61 (8.32 to 8.91) | £4732 (£4327 to £5166) | £4,633 | 5584 (5105 to 6096) | 5467 | 8.246 | 8.373 | 8.452 |
| M7: MPMRI for all men; TRUSB in men with suspicion of CS cancer. Re-biopsy with TRUSB those in whom CS cancer was not detected | 2 | 2 | 3 | 8.66 (8.37 to 8.97) | £5021 (£4612 to £5496) | £5,501 | 5924 (5442 to 6486) | 6491 | 8.276 | 8.412 | 8.495 |
| T7: TRUSB for all men; Men classified as NC or NON-CS receive a MPMRI. Men with suspicion of CS cancer receive a 2nd TRUSB | 2 | 2 | 3 | 8.69 (8.38 to 9.00) | £5194 (£4780 to £5682) | £5,778 | 6128 (5640 to 6705) | 6818 | 8.293 | 8.433 | 8.519 |
| M7: MPMRI for all men; TRUSB in men with suspicion of CS cancer. Re-biopsy with TRUSB those in whom CS cancer was not detected | 2 | 2 | 2 | 8.72 (8.40 to 9.04) | £5367 (£4947 to £5876) | £7,076 | 6333 (5837 to 6933) | 8350 | 8.304 | 8.449 | 8.538 |
| P4: TRUSB in all men and TPMB in men in whom CS cancer was not detected | 2 | Not applicable | | 8.74 (8.41 to 9.06) | £5968 (£5550 to £6490) | £30,084 | 7042 (6550 to 7658) | 35499 | 8.278 | 8.439 | 8.538 |

# Sensitivity analysis results

1. The results of the bivariate sensitivity analysis on the unit cost of tests are shown in Supplementary Figures 2-13. The unit costs of MPMRI and TRUSB were varied +/-50%; the unit cost of TRUSB is represented in the vertical axis whilst the unit cost of MPMRI is represented in the horizontal axis. Each figure refers to a unit cost of TPMB: base-case at £1,370, +25% at £1,713, -25% at £1,028 and the cost estimated for the PROMIS clinical study at £1,872 - and a cost-effectiveness thresholds of £13,000, £20,000 and £30,000/QALY. The square delineated in black represents the base-case unit cost of TRUSB and MPMRI. The figures show the cost-effective strategy for a combination of unit costs and are distinguished by colours: M7 222 in blue, T9 222 in purple, T7 222 in green, P1 in orange and P4 2-- in white.

**Supplementary Fig. 3 – Bivariate sensitivity analysis, TPMB cost=£1,370, cost-effectiveness threshold=£13,000(€15,398)/QALY**

Supplementary Fig. 4 – Bivariate sensitivity analysis, TPM-biopsy cost=£1,370, cost-effectiveness threshold=£20,000(€23,689)/QALY

Supplementary Fig. 5 – Bivariate sensitivity analysis, TPM-biopsy cost=£1,370, cost-effectiveness threshold=£30,000(€35,534)/QALY

Supplementary Fig. 6 – Bivariate sensitivity analysis, TPM-biopsy cost=£1,713, cost-effectiveness threshold=£13,000(€15,398)/QALY

Supplementary Fig. 7 – Bivariate sensitivity analysis, TPM-biopsy cost=£1,713, cost-effectiveness threshold=£20,000(€23,689)/QALY

Supplementary Fig. 8 – Bivariate sensitivity analysis, TPM-biopsy cost=£1,713, cost-effectiveness threshold=£30,000 (€35,534)/QALY

Supplementary Fig. 9 – Bivariate sensitivity analysis, TPM-biopsy cost=£1,028, cost-effectiveness threshold=£13,000(€15,398)/QALY

Supplementary Fig. 10 – Bivariate sensitivity analysis, TPM-biopsy cost=£1,028, cost-effectiveness threshold=£20,000 (€23,689)/QALY

Supplementary Fig. 11 – Bivariate sensitivity analysis, TPM-biopsy cost=£1,028, cost-effectiveness threshold=£30,000 (€35,534)/QALY

Supplementary Fig. 12 – Bivariate sensitivity analysis, TPM-biopsy cost=£1,872, cost-effectiveness threshold=£13,000 (€15,398)/QALY

Supplementary Fig. 13 – Bivariate sensitivity analysis, TPM-biopsy cost=£1,872, cost-effectiveness threshold=£20,000 (€23,689)/QALY

Supplementary Fig. 14 – Bivariate sensitivity analysis, TPM-biopsy cost=£1,872, cost-effectiveness threshold=£30,000 (€35,534)/QALY

1. The results of the scenario using Payment by Results (PbR) tariff for the unit cost of tests are represented in Supplementary Figure 14, showing the cost-effectiveness acceptability frontier. The x-axis represents values of cost-effectiveness threshold at £0-£50,000/QALY. The y-axis shows the probability that the cost-effective strategy on average is cost-effective.

Supplementary Fig. 15 – Cost-effectiveness acceptability frontier for the scenario using PbR tariff


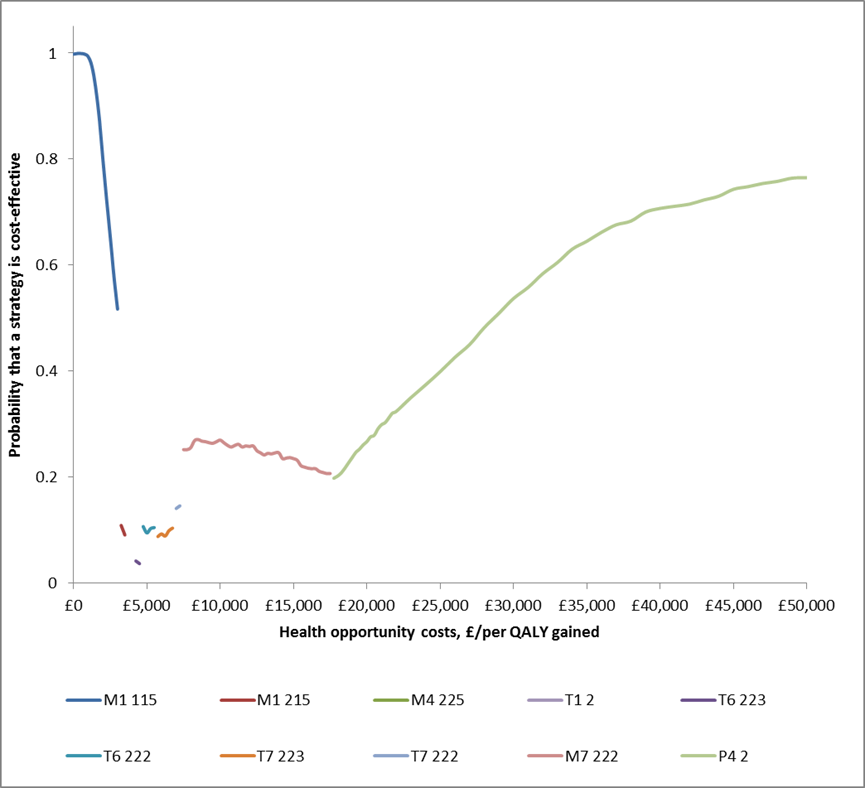


1. The results of the threshold sensitivity analysis are shown in Supplementary Table 28 as the change required to change the cost-effectiveness results.

Supplementary Table 28 – Results of the threshold sensitivity analysis

| **Analysis** | **Cost-effective strategy at the cost-effectiveness threshold** | | |
| --- | --- | --- | --- |
|  | **£13,000 (€15,398)/QALY gained** | **£20,000/ (€23,689)QALY gained** | **£30,000 (€35,534)/QALY gained** |
| Base case | M7 222 | M7 222 | M7 222 |
| TSA1: Changes in relative sensitivity of MRI-targeted TRUSB in detecting CS cancer; base-case= 1.2 | | | |
| between 1-1.1 | T7 222 | T9 222 | P4 2-- |
| between 1.15-1.19 | M7 222 | M7 222 | P4 2-- |
| between 1.20-1.50 | M7 222 | M7 222 | M7 222 |
| TSA2: Changes in the sensitivity of MRI-targeted 2nd TRUSB in detecting CS cancer; base-case = 0.87 | | | |
| between 0.92-1.00 | T7 222 | T9 222 | T9 222 |
| Between 0.87-0.92 | M7 222 | M7 222 | M7 222 |
| Between 0.78 -0.86 | M7 222 | M7 222 | P4 2-- |
| Between 0.67-0.77 | M7 222 | P4 2-- | P4 2-- |
| TSA3: Prevalence of intermediate risk vs low risk cancer; base-case=0.53 | | | |
| between 0.35-0.53 | No changes from base-case | | |
| TSA4: Probability of no cancer; base case=0.28 | | | |
| between 0.28-0.53 | No changes from base-case | | |
| TSA5: Risk of death from biopsy that changes cost-effective strategy; no risk at base case | | | |
| between 0.5-1.0% | M7 222 | P1 | P1 |
| risk=1.5% | N1 123 | P1 | P1 |
| risk=2% | N2 114 | N2 123 | P1 |
| TSA6: Reduced quality-adjusted survival from incorrect classification as no cancer | | | |
| QALY reduction =0.01 | M7 222 | M7 222 | P4 2-- |
| QALY reduction =0.09 | M7 222 | P4 2-- | P4 2-- |
| QALY reduction ≥0.1 | T9 222 | P4 2-- | P4 2-- |
| TSA7: Reduced effectiveness of radical prostatectomy | | | |
| Reduced by 10% | T7 223 | M7 222 | M7 222 |
| Reduced by 15% | M1 215 | T7 223 | M7 222 |
| Reduced by 20% | M1 115 | M1 115 | T6 222 |
| TSA8: Impact of repeated testing over time; base-case-0% of men are reclassified in the future | | | |
| 45%-50% | M7 222 | T9 222 | T9 222 |
| 50%-100% | T7 222 | T9 222 | T9 222 |

1. The results of the analysis on the value of future research are shown in Supplementary Table 29.

Supplementary Table 29 – Value of future research

| Cost-effectiveness threshold, /QALY | £13,000 | £20,000 | £30,000 |
| --- | --- | --- | --- |
| Cost-effective strategy | M7 222 | M7 222 | M7 222 |
| Net health of cost-effective strategy | 8.304 | 8.449 | 8.538 |
| Max net health achievable | 8.320 | 8.461 | 8.551 |
| Health loss due to parameter uncertainty per man referred for testing | 0.015 | 0.012 | 0.013 |
| Health loss due to parameter uncertainty per 39,000 men referred per year | 585 | 468 | 507 |
| Health loss due to parameter uncertainty over 5 years | 2,734 | 2,187 | 2,369 |
| Value of additional research per year | £7,605,000 | £9,360,000 | £10,140,000 |
| Value of additional research over 5 years | £35,538,767 | £43,740,021 | £47,385,023 |

# References

1. Roehl KA, Antenor JAV, Catalona WJ. Serial biopsy results in prostate cancer screening study. *The Journal of urology.* 2002;167(6):2435-2439.

2. Barzell WE, Melamed MR, Cathcart P, Moore CM, Ahmed HU, Emberton M. Identifying candidates for active surveillance: an evaluation of the repeat biopsy strategy for men with favorable risk prostate cancer. *The Journal of urology.* 2012;188(3):762-768.

3. Epstein JI, Pizov G, Walsh PC. Correlation of pathologic findings with progression after radical retropubic prostatectomy. *Cancer.* 1993;71(11):3582-3593.

4. Goto Y, Ohori M, Arakawa A, Kattan MW, Wheeler TM, Scardino PT. Distinguishing clinically important from unimportant prostate cancers before treatment: value of systematic biopsies. *The Journal of urology.* 1996;156(3):1059-1063.

5. Ahmed HU, Hu Y, Carter T, et al. Characterizing clinically significant prostate cancer using template prostate mapping biopsy. *The Journal of urology.* 2011;186(2):458-464.

6. Schoots IG, Roobol MJ, Nieboer D, Bangma CH, Steyerberg EW, Hunink MM. Magnetic resonance imaging–targeted biopsy may enhance the diagnostic accuracy of significant prostate cancer detection compared to standard transrectal ultrasound-guided biopsy: a systematic review and meta-analysis. *European urology.* 2015;68(3):438-450.

7. Wilt TJ, Brawer MK, Jones KM, et al. Radical prostatectomy versus observation for localized prostate cancer. *New England Journal of Medicine.* 2012;367(3):203-213.

8. James ND, Spears MR, Clarke NW, et al. Survival with newly diagnosed metastatic prostate cancer in the “Docetaxel Era”: data from 917 patients in the control arm of the STAMPEDE Trial (MRC PR08, CRUK/06/019). *European urology.* 2015;67(6):1028-1038.

9. Essink-Bot M-L, de Koning HJ, Nijs HG, Kirkels WJ, van der Maas PJ, Schröder FH. Short-term effects of population-based screening for prostate cancer on health-related quality of life. *Journal of the National Cancer Institute.* 1998;90(12):925-931.

10. Tsivian M, Abern MR, Qi P, Polascik TJ. Short-term functional outcomes and complications associated with transperineal template prostate mapping biopsy. *Urology.* 2013;82(1):166-170.

11. Torvinen S, Färkkilä N, Sintonen H, Saarto T, Roine RP, Taari K. Health-related quality of life in prostate cancer. *Acta Oncologica.* 2013;52(6):1094-1101.

12. Ara R, Brazier JE. Populating an economic model with health state utility values: moving toward better practice. *Value in Health.* 2010;13(5):509-518.

13. Department of Health. *Reference costs 2014-15.* 2016.

14. *Unit Costs of Health and Social Care 2015.* Canterbury: University of Kent; 2015.

15. NHS England, Monitor. *2014/15 National Tariff Payment System.* 2014.

16. National Institute for Health and Care Excellence (NICE). *Prostate cancer: diagnosis and management.* London: NICE;2014.

17. Lord J, Willis S, Eatock J, et al. Economic modelling of diagnostic and treatment pathways in National Institute for Health and Care Excellence clinical guidelines: the Modelling Algorithm Pathways in Guidelines (MAPGuide) project. 2013.

18. Briggs A, Sculpher M, Claxton K. *Decision modelling for health economic evaluation.* OUP Oxford; 2006.
